# Proteomic analysis on roots of *Oenothera glazioviana* under copper-stress conditions

**DOI:** 10.1038/s41598-017-10370-6

**Published:** 2017-09-06

**Authors:** Chong Wang, Jie Wang, Xiao Wang, Yan Xia, Chen Chen, Zhenguo Shen, Yahua Chen

**Affiliations:** College of Life Sciences, Jiangsu Collaborative Innovation Center for Solid Organic Waste Resource, National Joint Local Engineering Research Center for Rural Land Resources Use and Consolidation, Nanjing Agricultural University, Nanjing, Jiangsu China

## Abstract

Proteomic studies were performed to identify proteins involved in the response of *Oenothera glazioviana* seedlings under Cu stress. Exposure of 28-d-old seedlings to 50 μM CuSO4 for 3 d led to inhibition of shoot and root growth as well as a considerable increase in the level of lipid peroxidation in the roots. Cu absorbed by *O. glazioviana* accumulated more easily in the root than in the shoot. Label-free proteomic analysis indicated 58 differentially abundant proteins (DAPs) of the total 3,149 proteins in the roots of *O. glazioviana* seedlings, of which 36 were upregulated and 22 were downregulated under Cu stress conditions. Gene Ontology analysis showed that most of the identified proteins could be annotated to signal transduction, detoxification, stress defence, carbohydrate, energy, and protein metabolism, development, and oxidoreduction. We also retrieved 13 proteins from the enriched Kyoto Encyclopaedia of Genes and Genomes and the protein-protein interaction databases related to various pathways, including the citric acid (CA) cycle. Application of exogenous CA to *O. glazioviana* seedlings exposed to Cu alleviated the stress symptoms. Overall, this study provided new insights into the molecular mechanisms of plant response to Cu at the protein level in relation to soil properties.

## Introduction

Soil pollution by heavy metals deteriorates due to anthropogenic activities (e.g., metallurgy industry and sewage water irrigation), and it is a major problem of global concern^1^. Excess heavy metals (e.g. Cd, As, Hg, Se and Mo) severely reduce crop yields and cause health problems in humans, since they enter the food chain due to bioaccumulation in the edible parts of the plants^2^. Copper (Cu), as an essential micronutrient for plants, plays key roles in the citric acid (CA) cycle, pyruvate metabolism, and cell wall metabolism^3, 4^. However, excess Cu induces phytotoxicity, leading to growth inhibition, stunting, leaf chlorosis, necrosis and lipid peroxidation in membrane^5, 6^. The toxic rationales of Cu are due to its combination with nucleic acids and enzyme active sites^7, 8^. In addition, Cu inhibits the absorption of other elements such as Fe^9^. Long-term exposure to Cu results in low vegetation coverage and density^10^, thus, it is necessary to develop new plant varieties to make full use of such soil.

A better understanding of plants responses to heavy metal stress might help to develop effective detoxification measures and identify stress-tolerant genes or proteins^11^. Although the tolerance to Cu stress has been studied extensively at the phenotypic, physiological, and genetic level, and many candidate genes associated with heavy metal detoxification, tolerance, and stress response have been identified^12, 13^, the underlying mechanisms remain unclear, since gene expression is regulated at the transcriptional, translational, and post-translational level^1, 14^. Proteins have direct stress-acclimation functions that lead to changes in plasma membrane, cell cytoplasm, and the intracellular compartment composition^15^. Consequently, the plant response to heavy metal stress at the protein level needs further investigation.

Proteomics is one of the most advanced high-throughput biotechnological approaches that are used to address the biological function of proteins in response to different biotic or abiotic stresses^16, 17^. Previous proteomic studies on plant responses to heavy metal stress have mainly focused on Cd, Hg, and As^18–23^, whereas that fouced on Cu have been carried out in *Arabidopsis thaliana*, *Agrostis capillaris* L., *Cannabis sativa*, *Elsholtzia splendens*, *Triticum aestivum* L., and *Oryza sativa* ^10, 24–28^. The effects of heavy metals on plants vary with metal concentration and type, and also populations within a plant species. Therefore, further proteomic studies are needed in various species to investgate the molecular mechanisms of plants under Cu stress.

*Oenothera glazioviana* is a dominant species in the mine tailings of Tongling City, Anhui Province, China, which can efficiently stabilize Cu in the root and reduce its mobility and bioavailability^29^. Thus, *O*. *glazioviana* has been suggested as a potential candidate for the phytoexclusion of Cu-contaminated soils. However, little is known about the response mechanisms of *O. glazioviana* to Cu stress, especially at the protein level. In this study, a label-free quantitative proteomic approach based on nanoscale ultra-performance liquid chromatography tandem mass spectrometry (nano-UPLC-MS/MS) was conducted to identify Cu-responsive differentially abundant proteins (DAPs) in *O. glazioviana*. Our results in combination with physiological data might enhance our understanding regarding the interactions between *O. glazioviana* and Cu.

## Results

### Effects of Cu Stress on Phenotype and Growth Parameters

**Figure 1:**
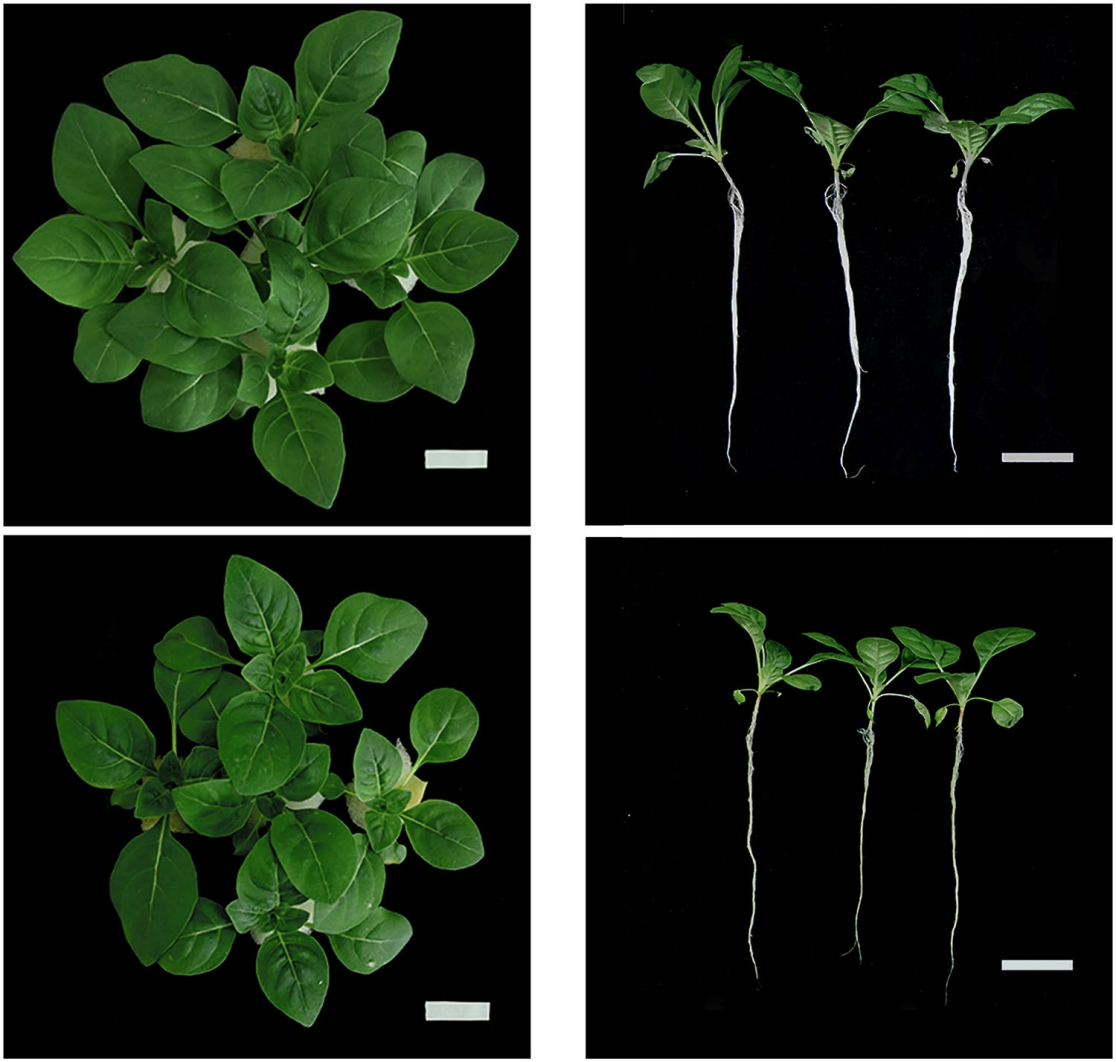
Phenotypic changes in *Oenothera glazioviana* seedlings exposed to 50 μM CuSO4 for 3 d. Upper left, control plants (vertical); upper right, control plants (horizontal); bottom left: plants exposed to copper (Cu; vertical); bottom right, plants exposed to Cu (horizontal).

*Oenothera glazioviana* seedlings exposed to 50 μM CuSO_4_ for 3 d did not show any leaf chlorosis or withering symptoms. However, a considerable reduction in the shoot and root growth was observed compared with the control (Fig. 1). Quantitative analysis showed that the root length, root tip number, root surface area, root volume, and leaf surface area of Cu treated seedlings were lower decreased by 5.9%, 58.3%, 76.2%, 39.1%, and 4.4%, respectively, compared with those of the control (Table 1). In addition, the shoot fresh weight (SFW), root fresh weight (RFW), shoot dry weight (SDW), and root dry weight (RDW) of Cu treated seedlings were significantly reduced by 9.2%, 16.9%, 47.2%, and 14.8%, respectively, compared with those of the control. The magnitude of Cu stress was higher in the roots than in the shoots. As shown in Fig. 1, the root of Cu treated seedlings is normal with only a few slightly brown parts.

**Table 1:** Effect of Cu stress on growth characteristics of *O*. *glazioviana*.

Physiological index	Control	Cu	Change fold (Control/Cu)
Root length (cm)	19.022 ± 1.80	17.970 ± 0.51	1.059
Root tips	382.67 ± 81.00	241.67 ± 27.54*	1.583
Root surface area (cm^2^)	40.67 ± 1.77	23.08 ± 6.47*	1.762
Leaf surface area (cm^2^)	11.25 ± 1.19	8.78 ± 0.57*	1.281
Root volume (cm^3^)	0.32 ± 0.01	0.23 ± 0.02**	1.391
Shoot fresh weight (g·plant^−1^)	2.49 ± 0.08	2.28 ± 0.05*	1.092
Root fresh weight (g·plant^−1^)	0.83 ± 0.05	0.71 ± 0.03*	1.169
Shoot dry weight (g·plant^−1^)	0.25 ± 0.04	0.17 ± 0.01*	1.471
Root dry weight (g·plant^−1^)	0.031 ± 0.001	0.027 ± 0.001**	1.148

Statistically significant differences are indicated with asterisks: (*) *p* < 0.05 or (**) p < 0.01. Data are given as means ± standard deviation (SD).

### Levels of Thiobarbituric Acid Reactive Substances (TBARS) and Cu in Leaves and Roots

**Figure 2:**
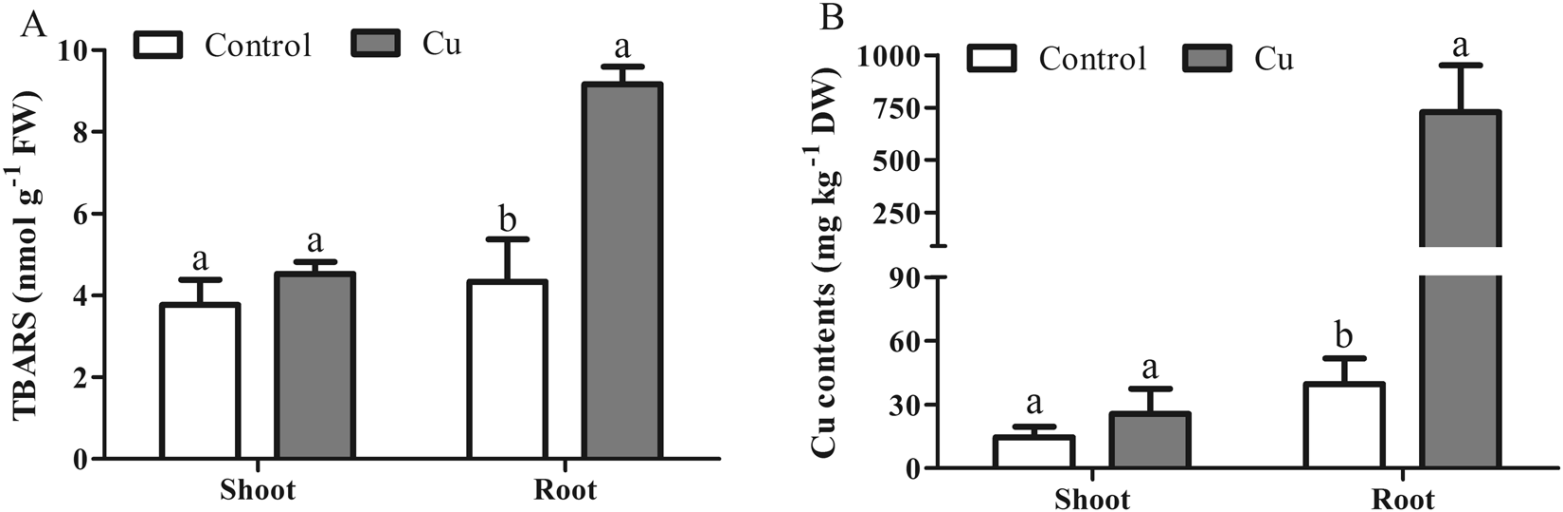
Effects of copper stress on the levels of (**A**) thiobarbituric acid reactive substances (TBARS) and (**B**) copper (Cu) in the leaves and roots of *Oenothera glazioviana* seedlings. Different letters indicate significant differences at *p* < 0.05. Bars represent one standard error. Each experiment was conducted in triplicate.

TBARS concentration in the shoot (4.53 ± 0.30 nmol g^−1^ FW) and the root (9.17 ± 0.43 nmol g^−1^ FW) of Cu treated seedlings was 1.15-fold and 2.03-fold higher, respectively, compared with that in the respective tissues of the control (3.77 ± 0.61 and 4.33 ± 1.04 nmol g-1 FW in the shoot and root, respectively) and also was 2.11-fold higher in the root than in the shoot (Fig. 2A). These results showed that the TBARS content in the root, but not in the shoot, was significantly affected by Cu stress. Similarly, the Cu concentration in the shoot (25.6 ± 11.7 μg g-1 DW) and the root (728.0 ± 223.7 μg∙g-1 DW) of Cu treated seedlings was 1.77-fold and 18.36-fold higher, respectively, compared with that in the respective tissues of the control (14.4 ± 5.1 and 39.6 ± 11.9 μg∙g-1 DW in the shoot and root, respectively) and also was 28.4-fold higher in the root than in the shoot (Fig. 2B).

### Proteome in *O. glazioviana* Roots in Response to Cu Stress

Through label free-based shotgun quantification approach, a total of 3149 proteins was successfully identified in *O. glazioviana* seedlings that treated or not with Cu (Table S1). Of these, 58 proteins (1.8% of the total proteins) were classified as DAPs (Table S2); 36 proteins were upregulated and 22 proteins were downregulated in response to Cu stress (Table 2).

**Table 2:** Identification of Differentially Expressed Protein Species in Roots of *O*. *glazioviana* Seedlings Exposed to Copper Stress for 3 Days.

No.	Accession^a^	Protein descriptions	Organism^b^	Convert UniprotKB^c^	Gene names	Unique peptides	Fold change^d^	*﻿p*﻿ value
**Protein Metabolism**								
1	Q9ZT91	Elongation factor Tu, mitochondrial	Arabidopsis thaliana	Q8W4H7	*TUFA*	2	6.69	9.7E-09
2	Q9SEI3	26 S protease regulatory subunit 10B homolog A	Arabidopsis thaliana	Q9SEI3	*RPT4A*	5	0.66	0.00028
3	P54778	26 S protease regulatory subunit 6B homolog	Solanum tuberosum	Q9SEI4	*RPT3*	3	0.59	0.00084
4	O04308	Probable mitochondrial-processing peptidase subunit alpha-2	Arabidopsis thaliana	O04308	*MPPA2*	2	0.28	1.3E-07
5	S8CE21	Peptidyl-prolyl cis-trans isomerase	Genlisea aurea	P34790	*M569_09669*	2	0.55	0.00018
6	A8MRZ7	Translational initiation factor 4A-1	Arabidopsis thaliana	P41376	*EIF4A1*	3	0.61	0.00129
7	Q9FZ48	Ubiquitin-conjugating enzyme E2 36	Arabidopsis thaliana	Q9FZ48	*UBC36*	2	0.62	3.8E-05
8	G7IRR6	Protein disulfide-isomerase	Medicago truncatula	Q9FF55	*MTR_2g094180*	2	0.64	0.00474
9	J7KE88	Heat shock protein 90	Lactuca sativa	O03986	*HSP90*	2	1.55	0.00108
10	Q9LTX9	Heat shock 70 kDa protein 7, chloroplastic	Arabidopsis thaliana	Q9LTX9	*HSP70-7*	2	2.43	3E-09
11	P30707	60 S ribosomal protein L9	Pisum sativum	P49209	*RPL9*	2	3.52	0.00052
12	P51430	40 S ribosomal protein S6-2	Arabidopsis thaliana	P51430	*RPS6B*	2	2.38	0.00021
13	O81361	40 S ribosomal protein S8	Prunus armeniaca	Q9FIF3	*RPS8*	2	2.38	9E-07
14	Q9SXU1	Proteasome subunit alpha type-7	Cicer arietinum	O24616	*PAD1*	4	1.50	0.00726
15	Q9MTJ8	ATP-dependent Clp protease proteolytic subunit	Oenothera hookeri	P56772	*clpP*	3	0.50	0.00263
16	P68173	Adenosylhomocysteinase	Nicotiana tabacum	O23255	*SAHH*	2	2.13	2.7E-06
17	Q949 × 7	Diaminopimelate decarboxylase 1, chloroplastic	Arabidopsis thaliana	Q949 × 7	*LYSA1*	4	0.52	3.9E-05
18	Q940P8	T-complex protein 1 subunit beta	Arabidopsis thaliana	Q940P8	*CCT2*	2	0.21	1.7E-07
**Carbohydrate and Energy Metabolism**								
19	Q9LXS7	Citrate synthase 1	Arabidopsis thaliana	Q9LXS7	*CSY1*	2	3.17	8.9E-08
20	S8E148	Pyruvate dehydrogenase E1 component subunit alpha	Genlisea aurea	P52901	*M569_08768*	1	2.68	0.00052
21	P93819	Malate dehydrogenase, cytoplasmic 1	Arabidopsis thaliana	P93819	*MDH1*	4	2.28	0.00057
22	M0TRQ8	Succinyl-CoA ligase subunit beta	Musa malaccensis	O82662	*N/A*	2	1.55	0.01987
23	T1E156	ATP synthase subunit gamma	Silene latifolia	Q96250	*ATP3*	2	1.81	0.0081
24	Q7M2G6	ATP synthase subunit alpha	Oenothera villaricae	P92549	*ATP1*	3	1.58	0.00195
25	Q9FKK7	Xylose isomerase	Arabidopsis thaliana	Q9FKK7	*XYLA*	2	1.84	4.7E-05
26	O49845	Sucrose synthase 4	Daucus carota	Q9LXL5	*SUS4*	2	2.32	0.00039
27	P54243	Glucose-6-phosphate isomerase, cytosolic	Oenothera mexicana	Q8H103	*PGIC*	7	1.57	0.00054
28	F4JLP5	Dihydrolipoyl dehydrogenase 2, chloroplastic precursor	Arabidopsis thaliana	F4JLP5	*LPD2*	2	1.56	0.00644
29	Q94KU2	6-phosphogluconate dehydrogenase, decarboxylating 2, chloroplastic	Spinacia oleracea	Q9FFR3	*pgdP*	3	1.79	1.8E-05
30	Q9SJB3	ATPase 5, plasma membrane-type	Arabidopsis thaliana	Q9SJB3	*AHA5*	2	2.15	8.2E-07
31	Q9LU41	Calcium-transporting ATPase 9, plasma membrane-type	Arabidopsis thaliana	Q9LU41	*ACA9*	2	3.38	0.02257
32	P37829	Fructokinase	Solanum tuberosum	Q9M1B9	*N/A*	3	3.73	1.3E-08
33	B9T118	NADH-ubiquinone oxidoreductase, putative	Ricinus communis	Q9FGI6	*RCOM_0458390*	3	0.60	0.00043
**Signal Transduction**								
34	P40392	Ras-related protein RIC1	Oryza sativa	P28188	*RIC1*	1	0.35	6E-07
35	O80501	Ras-related protein RABH1b	Arabidopsis thaliana	O80501	*RABH1B*	4	0.38	2.1E-06
36	P11574	V-type proton ATPase subunit B1	Arabidopsis thaliana	P11574	*VHA-B1*	15	1.83	3.5E-06
37	B7SDI4	Aquaporin	Oryza sativa	Q39196	*N/A*	2	0.61	4.6E-06
38	B6T451	Importin subunit alpha	Zea mays	Q96321	*N/A*	3	0.57	0.00233
39	P30184	Leucine aminopeptidase 1	Arabidopsis thaliana	P30184	*LAP1*	2	2.38	1.2E-08
40	Q9LXC0	GDP dissociation inhibitor	Arabidopsis thaliana	Q9LXC0	*At5g09550*	3	2.40	0.00036
41	A7PZL3	Probable polygalacturonase	Vitis vinifera	Q9SMT3	*GSVIVT00026920001*	2	1.70	0.00197
**Detoxification and Stress Defense**								
42	V7BP31	Lactoylglutathione lyase	Phaseolus vulgaris	F4IAH9	*PHAVU_006G149400g*	3	1.92	3.5E-05
43	P85929	Nucleoside diphosphate kinase 1	Pseudotsuga menziesii	P39207	*NDK1*	2	1.98	0.00015
44	C6TBN2	Probable aldo-keto reductase 1	Glycine max	O22707	*AKR1*	3	1.97	8.7E-06
45	Q9SU63	Aldehyde dehydrogenase family 2 member B4, mitochondrial	Arabidopsis thaliana	Q9SU63	*ALDH2B4*	2	1.71	1.8E-05
46	P31426	Phenylalanine ammonia-lyase 2	Solanum tuberosum	P35510	*PAL-2*	2	1.64	0.00323
47	Q39471	Isopentenyl-diphosphate Delta-isomerase II	Clarkia breweri	Q42553	*IPI2*	6	2.73	1.2E-05
48	Q9S7A0	Probable glutamate dehydrogenase 3	Arabidopsis thaliana	Q9S7A0	*GSH3*	2	1.55	0.00674
**Development**								
49	B9RT61	Translationally-controlled tumor protein homolog	Ricinus communis	P31265	*RCOM_0681260*	2	0.38	8.5E-06
50	O04331	Prohibitin-3, mitochondrial	Arabidopsis thaliana	O04331	*PHB3*	3	0.59	0.00012
51	Q0WM29	Methylmalonate-semialdehyde dehydrogenase [acylating], mitochondrial	Arabidopsis thaliana	Q0WM29	*ALDH6B2*	2	0.64	0.0009
52	Q76H85	Histone H4	Silene latifolia	Q9MAU3	*SlH4*	3	0.14	1.1E-07
53	D7LSV8	ADP-ribosylation factor	Lyre-leaved rock-cress	Q9M1P5	*ARALYDRAFT_486735*	9	0.50	1E-06
**Oxidoreduction**								
54	D7UC38	Phosphomannomutase	Vitis vinifera	O80840	*VIT_15s0046g03520*	1	2.10	2.4E-06
55	Q43873	Peroxidase 73	Arabidopsis thaliana	Q43873	*PER73*	2	1.86	2E-08
56	Q93VR3	GDP-mannose 3,5-epimerase	Arabidopsis thaliana	Q93VR3	*At5g28840*	5	1.90	2.8E-09
**Unknown**								
57	A5B8T3	Putative uncharacterized protein	Vitis vinifera	None	*VIT_05s0102g00710*	1	0.63	0.01125
58	D7SW76	Putative uncharacterized protein	Vitis vinifera	Q8RWN9	*VIT_07s0031g01740*	2	0.61	0.00054

^a^Accession, accession number according to the UniProtKB database; ^b^Organism, plant species; ^c^Convert UniprotKB, versus the Arabidopsis thaliana Omicsbean database; ^d^Fold change, the ratio between proteins content of identified protein in treated vs control.

**Figure 3:**
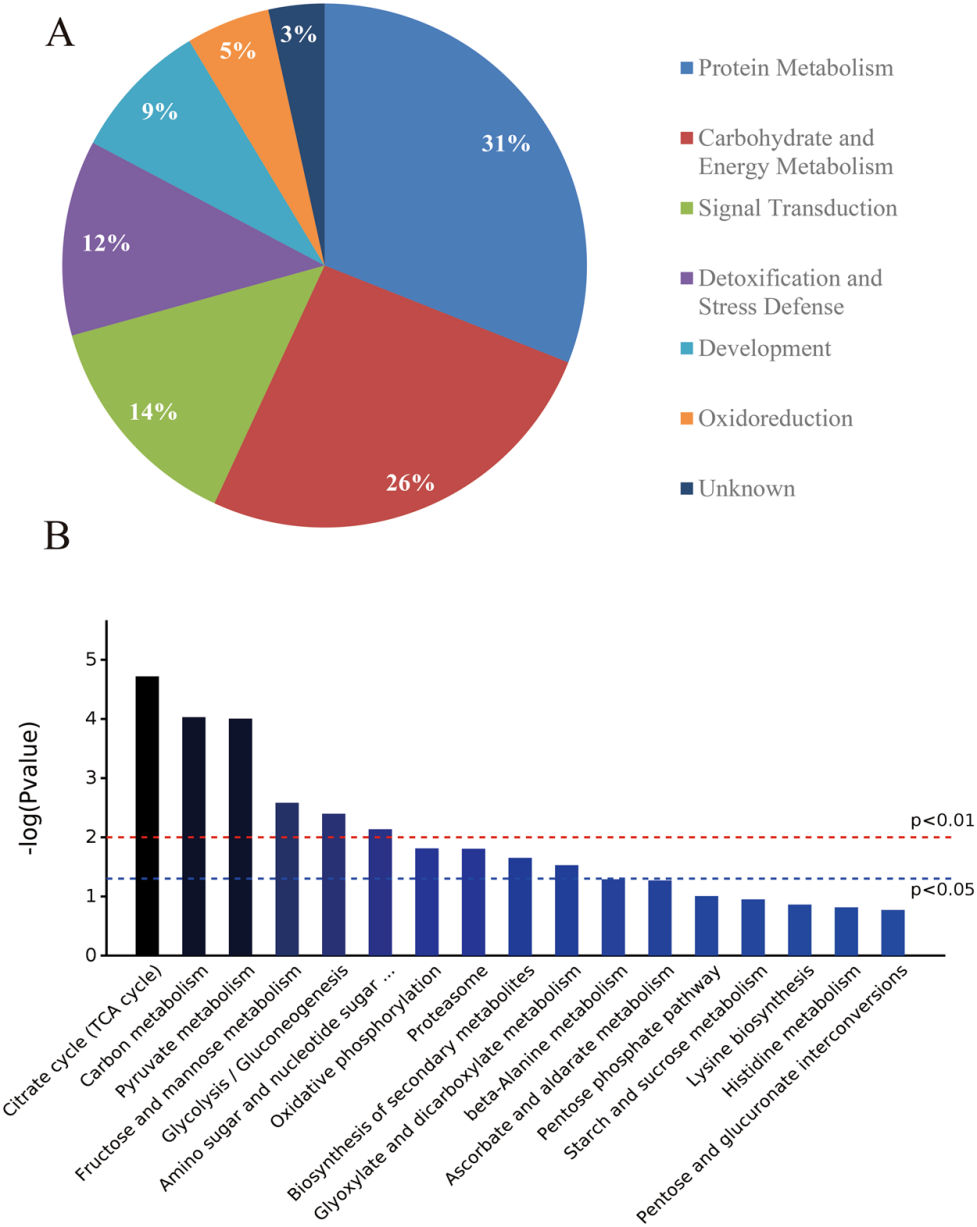
(**A**) Gene Ontology (GO) and (**B**) Kyoto Encyclopaedia of Genes and Genomes (KEGG) analysis of 58 differentially expressed proteins (DAPs) in the roots of *Oenothera glazioviana* seedlings. Pie charts show the distribution of 58 DAPs on of the Cu-responsive proteins into their functional classes in percentage. Pathways are coloured from blue (lowest *p* value) to black (highest *p* value).

To gain a better understanding of the molecular functions and biological processes involved in *O*. *glazioviana* response to Cu stress, Gene Ontology (GO) analysis was performed and showed that DAPs were annotated to protein metabolism (18 DAPs), carbohydrate and energy metabolism (15 DAPs), signal transduction (eight DAPs), detoxification and stress defence (seven DAPs), development (five DAPs), oxidoreduction (three DAPs), and other unknown functions (two DAPs) (Fig. 3A).

**Figure 4:**
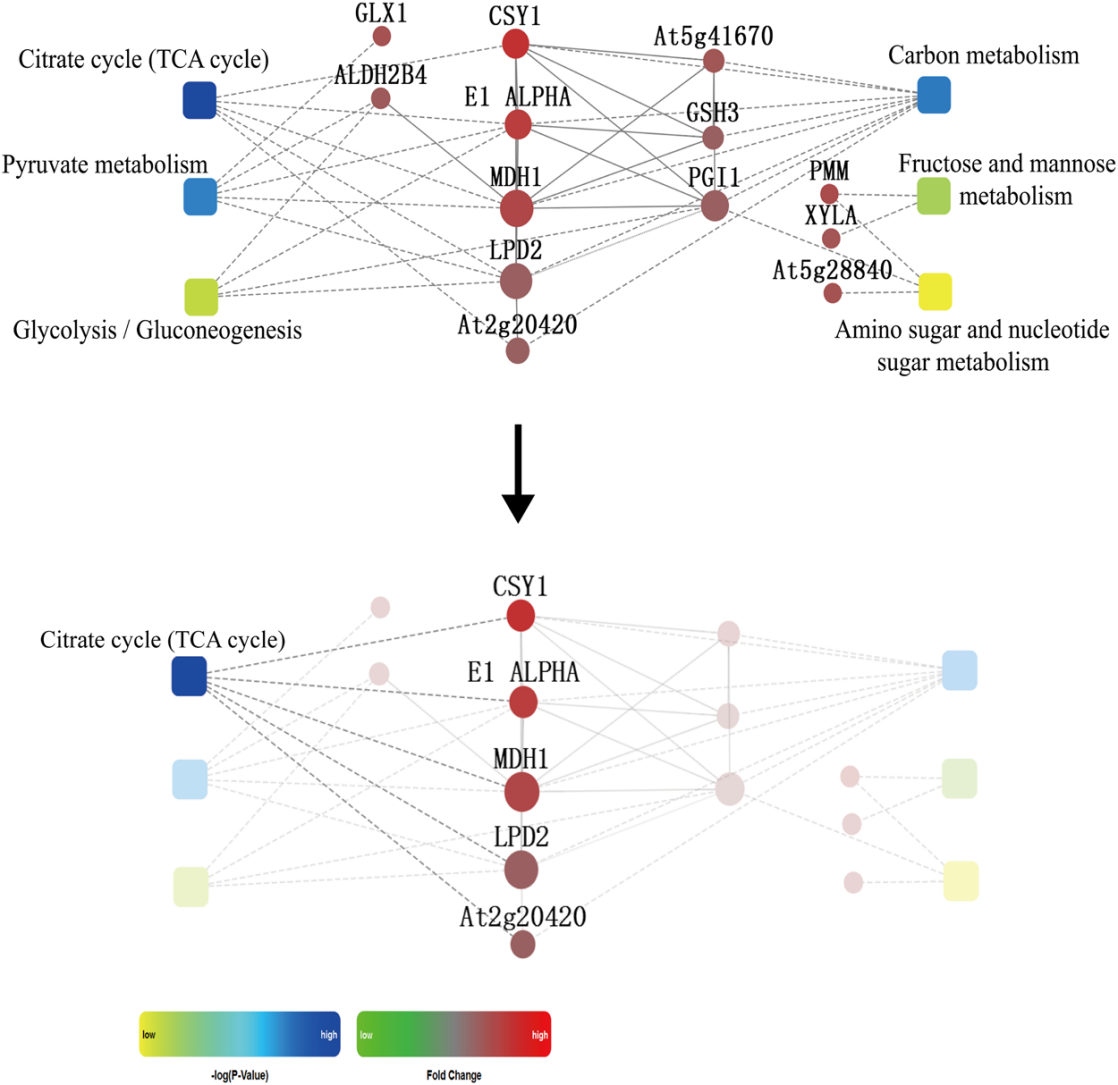
Interaction network of the Kyoto Encyclopaedia of Genes and Genomes (KEGG) pathway and biological processes based on protein fold change at *p* < 0.01. Circle nodes refer to proteins (red, up-regulation; green, down-regulation). Rectangles refers to KEGG pathway or biological process (yellow, lowest *p* value; blue, highest *p* value).

The Kyoto Encyclopaedia of Genes and Genomes (KEGG) analysis indicated that six pathways (involved 13 DAPs), including the CA cycle, carbon metabolism, pyruvate metabolism, fructose and mannose metabolism, glycolysis/gluconeogenesis, and amino sugar and nucleotide sugar metabolism, were significantly enriched (*p* < 0.01) (Fig. 3B; Table S3). The CA cycle was the most significantly enriched (*p* = 1.91e-05; Fig. 4; Table S4), and the citrate synthase was the most up-regulated among these 13 DAPs.

### Effect of Exogenous CA Application on Cu Tolerance

**Figure 5:**
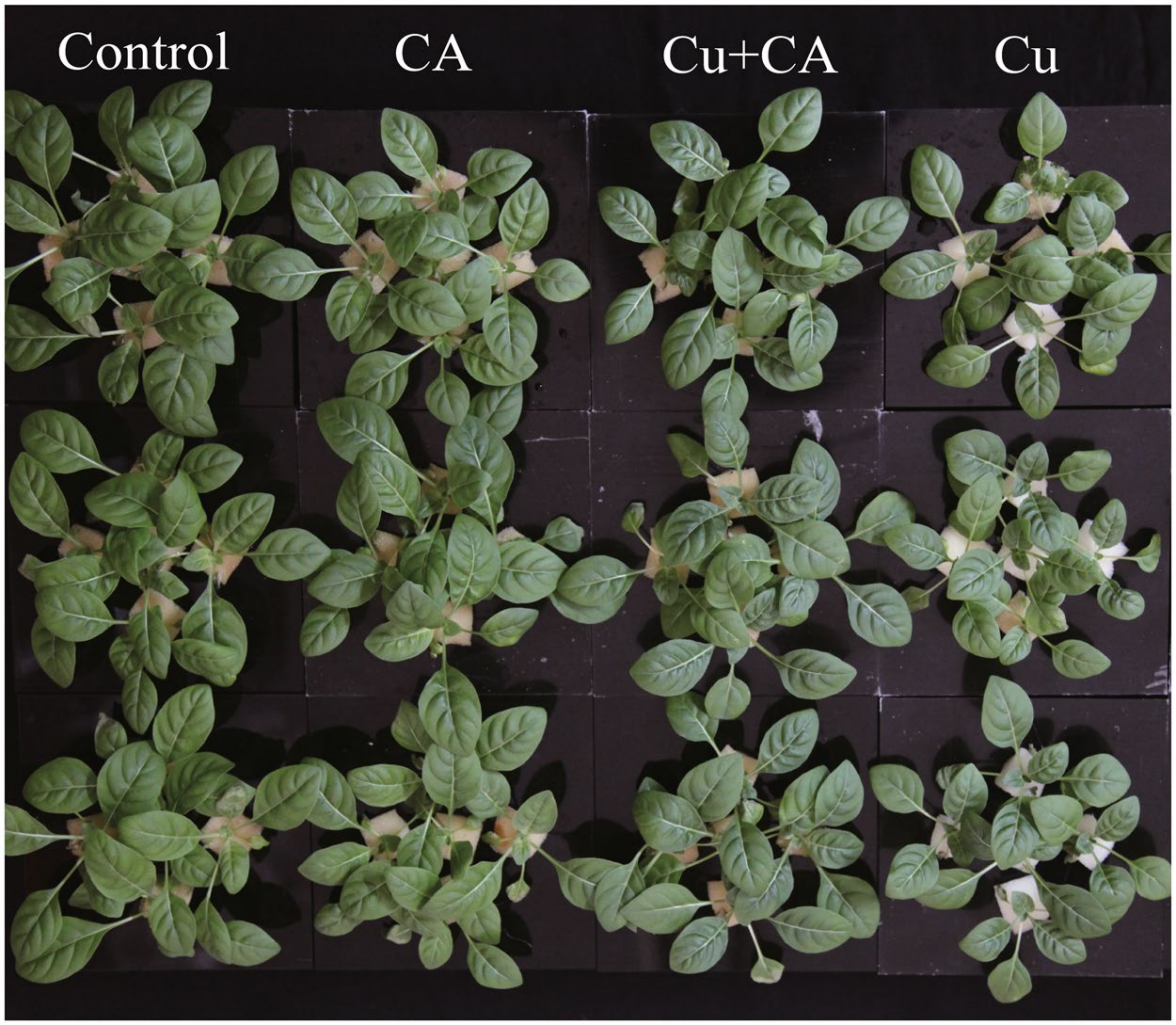
Phenotypic changes in *Oenothera glazioviana* seedlings exposed to 50 μM CuSO_4_ for 3 d with exogenous application of 50 µM citric acid.

The application of exogenous CA to *O*. *glazioviana* seedlings exposed to 50 μM CuSO_4_ for 3 d greatly alleviated stress symptoms (Fig. 5). Quantitative analysis showed that the fresh and dry weights of Cu + CA treated seedlings were significantly higher than those of the Cu treated seedlings. However, the TBARS content in the root of Cu + CA treated seedlings was significantly lower than that in the root of the Cu treated seedlings (Table 3).

**Table 3:** Effect of exogenous CA application on growth characteristics and TBARS contents of *O*. *glazioviana* seedlings under Cu stress for 3 d.

Treatment	Fresh wegiht (g/plant)				Dry wegiht (g/plant)				TBARS contents (nmol/L FW)			
	Leaf		Root		Leaf		Root		Leaf		Root	
	M ± SD	%	M ± SD	%	M ± SD	%	M ± SD	%	M ± SD	%	M ± SD	%
Control	2.55 ± 0.10^a^	100	0.80 ± 0.04^ab^	100	0.32 ± 0.05^a^	100	0.031 ± 0.002^a^	100	3.87 ± 0.61^a^	100	4.53 ± 1.03^c^	100
CA	2.43 ± 0.03^a^	95.3	0.83 ± 0.03^a^	103.8	0.31 ± 0.03^a^	96.9	0.032 ± 0.002	103.2	3.93 ± 0.22^a^	101.6	4.58 ± 0.64	101.1
Cu	2.22 ± 0.06^c^	87.1	0.67 ± 0.03^c^	83.8	0.20 ± 0.02^c^	62.5	0.025 ± 0.001^c^	80.6	4.55 ± 0.29^a^	117.6	9.26 ± 0.43^a^	204.4
Cu + CA	2.34 ± 0.02^b^	91.8	0.75 ± 0.03^b^	93.8	0.26 ± 0.01^b^	81.3	0.028 ± 0.002^b^	90.3	4.43 ± 0.87^a^	102.7	6.48 ± 0.56^b^	143

Different letters in the same column indicate a significant difference at P < 0.05. Data are given as means (M) ± standard deviation (SD).

## Discussion

Cu is an essential trace element in plants; however, in excess concentrations, it induces a wide range of biochemical effects and metabolic disturbances, which are responsible for a strong growth inhibition. The root growth is more susceptible to Cu toxicity than the shoot growth either the plant grows in the soil^30^ or in a culture solution^31^. In our study, *O*. *glazioviana* seedlings showed visible damage when exposed to 50 μM CuSO_4_ for 3 d. The roots became slightly brown (Fig. 1), and their growth was markedly inhibited. The root tip number, root surface area, root volume, and leaf surface area of Cu treated seedlings were significantly lower compared with those of the control (Table 1). The inhibitory effect of Cu on the root growth may be due to the reduced cell root meristem division and proliferation, damaged cell integrity in the root transition zone and retarded normal root cell growth^32^. These results were in agreement with those reported in findings in maize, hemp, and tobacco^25, 33, 34^.

Plants accumulate readily more Cu in the root than in other tissues such as leaves^26^. In *Brassica napus*, Cu accumulation increases significantly with Cu exposure and is higher in the root, followed by that in the stem and leaf^35^. In our study, the Cu content in the root and the shoot of Cu treated seedlings was 18.36-fold and 1.77-fold higher, respectively, compared with that in the respective tissues of the control (Fig. 2B), revealing the low translocation coefficient of Cu; thus, the shoot was less stressed. Excessive metal(loid) exposure, especially to Cu, generates reactive oxygen species (ROS) that damage the plant cells and inhibit plant growth^36^. TBARS, as a product of lipid peroxidation, is a sensitive biomaker of oxidative damage^37^. Here, the level of TBARS did not change significantly in the shoot, but significantly increased in the root of Cu treated seedlings (Fig. 2A), confirming previous studies in *O*. *glazioviana* ^29^ and suggesting that the tolerance/accumulation mechanism in the roots might restrict the root-to-shoot transfer of Cu.

The morphological and physiological changes exhibited in *O*. *glazioviana* seedlings exposed to 50 μM CuSO_4_ for 3 d suggested that the metabolic and biological processes are regulated by Cu application. Using the label free-based shotgun quantification method, we found that the abundance of 58 proteins significantly changed in Cu treated seedlings compared with the control. The Cu-responsive proteins were related to a wide range of molecular functions, including protein metabolism (31%), carbohydrate and energy metabolism (26%), signal transduction (14%), detoxification and stress defence (12%), development (9%), oxidoreduction (5%), and other unknown functions (3%). The observed diversity in the biological functions of DAPs suggested that the response of *O*. *glazioviana* to Cu stress might be a complex process, and some physiological and biochemical changes were altered to counteract the adverse conditions.

### Protein Metabolism

Previous study in graph shown that Cu exposure markedly affects the protein metabolism and leads to protein reduction^38^. Here, 19 DAPs were identified in the roots of *O*. *glazioviana* seedlings exposed to Cu. Among these, the elongation factor Tu (No. 1; Fc = 6.69) catalyses the extension of the amino acid chain on the ribosome that further controls protein synthesis; heat shock proteins (No. 9, Fc = 1.55; No. 10, Fc = 2.43) increase in abundance under various abiotic stresses, since they prevent the aggregation of non-native proteins under normal and stress conditions^39^. Peptidyl-prolyl cis-trans isomerase and protein disulfide isomerase (No. 5, Fc = 0.55; No. 8, Fc = 0.64) play an important role in the maturation of newly synthesized proteins by correcting improper fold^40^; and ubiquitin-conjugating enzymes (UBCs, No. 7, Fc = 0.62) catalyse the second step in the ubiquitin-dependent proteolytic pathway that is one of the major protein degradation pathways in eukaryote. UBCs are induced under stress conditions and are responsible for the selective degradation of proteins with incorrect folding^41^. Under Cu stress conditions, we observed the up-regulation of No. 1, 9, and 10 that suggested the accumulation of damaged or misfolded proteins under Cu stress. Whereas the down-regulation of No. 5, 7, and 8 that indicated the synthesis of inappropriate proteins that led to the abnormal growth of *O*. *glazioviana* seedlings.

### Carbohydrate and Energy Metabolism

The CA cycle is an important pathway in energy metabolism, responsible for the oxidation of respiratory substrates that lead to ATP synthesis and the adaptation to unfavourable environments^42, 43^. Here, we identified five proteins, citrate synthase 1 (No. 19, Fc = 3.17), succinyl-CA ligase subunit beta (No. 22, Fc = 1.55), pyruvate dehydrogenase E1 component subunit alpha (No. 20, Fc = 2.68), malate dehydrogenase, cytoplasmic 1 (No. 21, Fc = 2.28), dihydrolipoyl dehydrogenase 2, chloroplastic precursor (No. 28, Fc = 1.56), and 6-phosphogluconate dehydrogenase, decarboxylating 2, chloroplastic (No. 29, Fc = 1.79) that were involved in the CA cycle. Pyruvate dehydrogenase catalyzes the conversion of pyruvate to acetyl-CoA, and links the glycolysis pathway to the TCA cycle^44^. In previous studies, the expression of citrate synthase gene increased the citrate synthase activity and the citric acid content^45^. In the present study, we identified citrate synthase and used exogenous CA to experimentally verify its role in the alleviation of Cu stress symptoms. We also identified glucose-6-phosphate isomerase, cytosolic (No. 27, Fc = 1.57), which suggested that the glycolytic pathway might be involved in plant response to Cu stress. Shu *et al*. showed that enhanced glycolysis leads to the accumulation of acetyl-CoA in the CA cycle and the increased production of ATP to support stress resistance^46^. Here, most of the identified glycolysis-related proteins were up-regulated, indicating that *O*. *glazioviana* seedlings could maintain their essential respiration and provide more glycolytically generated ATP by reinforcing the CA cycle and glycolytic pathway under Cu stress conditions.

### Signal Transduction

Many transporters, such as V-type proton ATPase subunit B1 (No. 36, Fc = 1.83), aquaporin (No. 37, Fc = 0.61), importin subunit alpha (No. 38, Fc = 0.57), and GDP dissociation inhibitor (No. 40, Fc = 2.40) were identified in the present study. V-type proton ATPase changes the H^+^ electrochemical gradient in the vacuole membrane^47^. Fukuda *et al*. shown that salt stress increases the transcription level of V-ATPase in the root of barley seedlings, which is beneficial for the ion accumulation in the vacuole^48^. Aquaporins are major water transporters that participate in the detoxification and compartmentalization of heavy metals^49^. The activity and expression of aquaporins and V-type proton ATPase can be affected by many external stimuli such as salinity^50^ and heavy metals^51^. Two small GTP-binding proteins, Ras-related protein RIC1 (No. 34, Fc = 0.34) and Ras-related protein RABH1b (No. 35, Fc = 0.38), play vital roles in signaling, the nuclear transportation of proteins and RNAs, and the regulation of cell cycle progression^52^. In the present, we found that Cu stress induced the up-regulation of Aquaporin that might influence the intracellular transport of Cu, as well as the up-regulation and activation of V-type proton ATPase that led to the excessive accumulation of Cu in the vacuole.

### Detoxification and Stress Defence

We found several proteins related to cell detoxification, including Aldo-keto reductase (No. 44, Fc = 1.97) that is known to be effective in the detoxification of lipid peroxidation-derived reactive aldehydes^53, 54^. Transgenic tobacco plants overexpressing *alfalfa* AKR (*MsALR*) showed increased tolerance against a variety of oxidative stresses induced by methylviologen, heavy metals, and long-term drought^53–55^. Aldehyde dehydrogenase (No. 45, Fc = 1.71) is considered as a general detoxifying enzyme that eliminate toxic biogenic and xenobiotic aldehydes^56^. *Cp-ALDH* and *Ath-ALDH3* from *Craterostigma plantagineum* and *A*. *thaliana*, respectively, respond to a variety of stress treatments^57^. Two ALDHs from barley were also shown to be up-regulated by drought stress^58^. Our proteomic analysis indicated ALDH might be associated with the removal of harmful substances under Cu stress in *O*. *glazioviana* seedlings.

### Development

Translationally controlled tumour protein (No. 49, Fc = 0.38) is considered as a major regulator of cell growth in plants. Prohibitins (No. 50, Fc = 0.59) play an important role in root hair elongation, cell division, and development^59^. Methylmalonate-semialdehyde dehydrogenase (No. 51, Fc = 0.64) is a mitochondrial enzyme involved in the distal part of the valine and pyrimidine catabolic pathways. MMSDH was decreased in the seminal roots of *slr1* mutants that were thinner compared with those of the wild type, supporting that MMSDH is a key factor in root development^60^. ADP-ribosylation factor (No. 53, Fc = 0.50) participates in membrane traffic, since it regulates the normal auxin efflux to exert a positive function in the cell polar localization^61–63^. The down-regulation of the ADP-ribosylation factor in *A*. *thaliana* results in severe growth inhibition^64^. In the present study, No. 49, 50, 51, and 53 were down-regulated in the roots of Cu treated seedlings, revealing that these proteins might be involved in growth inhibition.

### Oxidoreduction

Cu, as a redox-active metal, can catalyse the formation of hydroxyl radicals to generate ROS that create oxidative stress and damage cellular macromolecules, resulting in cell death^65^. Plants have developed a vigorous antioxidant mechanism that is associated with enzymatic (peroxidase) and non-enzymatic components (glutathione). Here, phosphomannomutase (No. 54, Fc = 2.10), GDP-mannose 3,5-epimerase (No. 56, Fc = 1.90), and peroxidase 73 (No. 55, Fc = 1.86) that play crucial roles in ROS scavenging were accumulated in the roots of *O*. *glazioviana* seedlings exposed to Cu, suggesting that they might be associated with oxidative stress response.

Proteins do not perform their functions as single entities, but together in networks^14^. Meanwhile, signal molecules, usually help plants to recognize environmental factors, and regulate the expression of related genes in the signal pathways. When exogenous CA was applied to *O*. *glazioviana* seedlings exposed to 50 mM CuSO_4_, the stress symptoms were alleviated (Fig. S1, Table 3), indicating that CA might act as a signal molecule and regulate the expression of several proteins through a direct or indirect mechanism under Cu stress conditions.

Overall, our study showed that Cu stress inhibited the growth of *O*. *glazioviana* seedlings and increased the root Cu concentration. Our proteomic analysis identified 58 DAPs in the roots of *O*. *glazioviana* seedlings involved in protein metabolism, carbohydrate metabolism, signal transduction, detoxification and stress defence, development, and oxidoreduction. Using KEGG and PPI analysis, we identified 13 DAPs that were involved in different pathways. The CA cycle was the most significantly enriched, and then the citrate synthase exhibited most up-regulated among these 13 DAPs. These results suggested that CA might play a critical role in the overall plant response process to Cu. Subsequently, we applied exogenous CA to Cu treated seedlings in order to verify our assumption. We found that exogenous CA alleviated Cu stress symptoms, probably because it regulates the expression of proteins related to plant response to Cu stress. These results provided new insights into the molecular mechanisms of plant response to Cu.

## Methods

### Ethic Statement

No permissions were required for collecting *O*. *glazioviana* seeds from the Cu mine tailings in Tongling City, Anhui Province, China. *O*. *glazioviana* is not an endangered or protected plant species. The authors maintained the population at sustainable levels. The study was conducted following the national and international guidelines.

### Plant Growth Conditions and Cu Treatments

**Figure 6:**
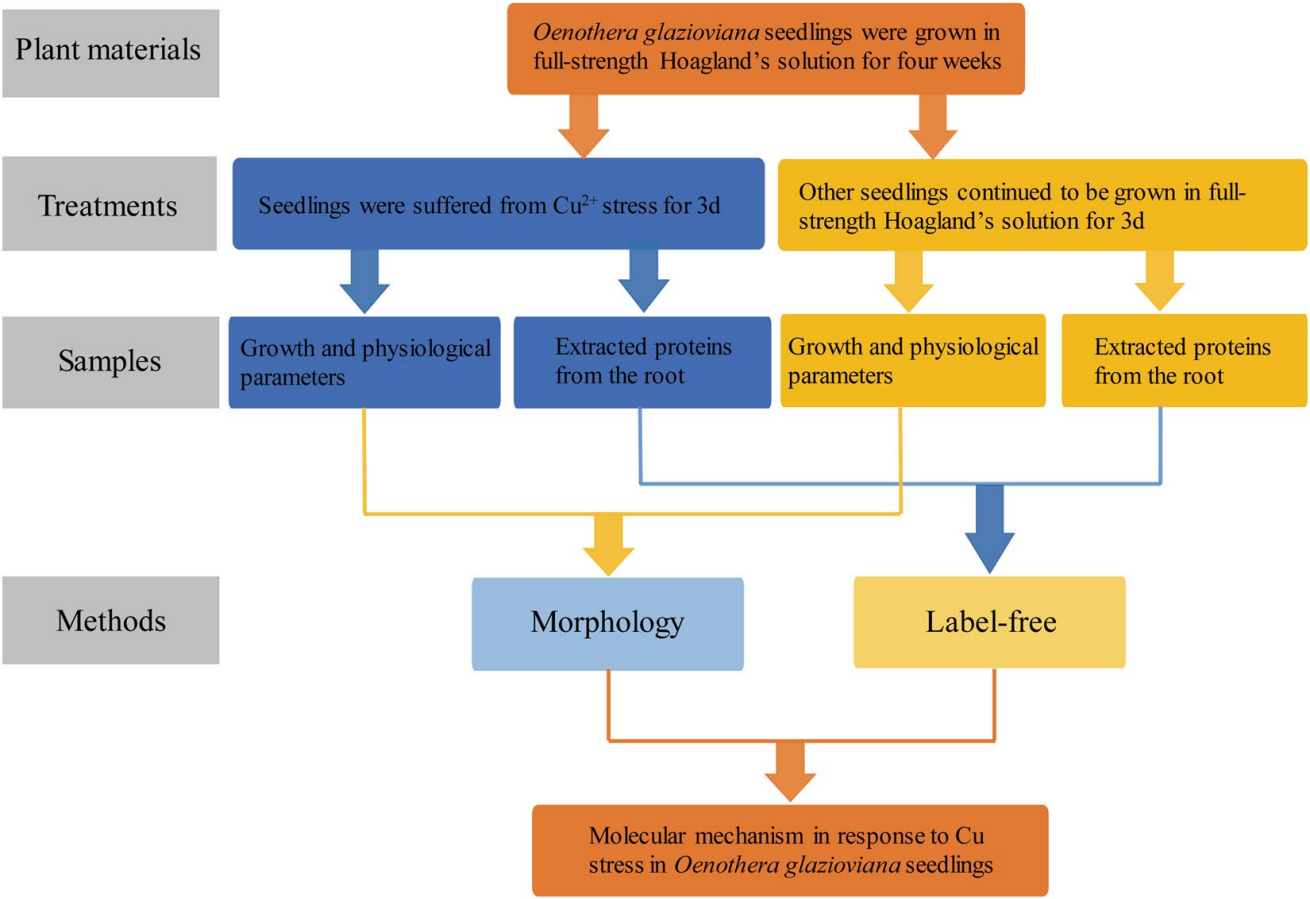
Scheme about the experimental setup to compare Cu-stressed *Oenothera glazioviana* seedlings with unstressed (control) using proteomic analysis.

The study design is shown in Fig. 6. *O*. *glazioviana* seeds were soaked in distilled water for 24 h and then, sown in plastic pots filled with vermiculite. The pots were placed in a growth chamber at a 12 h day/12 h night photoperiod, 20 °C day/25 °C night temperature, and light intensity of 250 μmol m^−2^ s^−1^. The cotyledons opened at approximately 7 d after sowing. The seedlings were fixed in cystose and transferred to vessels with 1 L of Hoagland’s nutrient solution, consisted of 5 mM Ca(NO_3_)_2_, 5 mM KNO_3_, 1 mM KH_2_PO_4_, 50 μM H_3_BO_3_, 1 mM MgSO_4_, 4.5 μM MnCl_2_, 3.8 μM ZnSO_4_, 0.32 μM CuSO_4_, 0.1 mM (NH_4_)_6_Mo_7_O_24_, and 10 μM Fe-ethylenediaminetetraacetic acid (EDTA). The nutrient solution was renewed every 3 d. After 21 d, the seedlings were exposed to 50 μM CuSO_4_ for 3 d. Each treatment (10 plants) was conducted in five replicates, and the control plants were grown in Hoagland’s nutrient solution without the addition of Cu. Plant roots and shoots were cut, pooled together, rinsed in deionized water, flash frozen in liquid nitrogen, and stored at −80 °C until analysis.

### Growth Parameters

The maximum shoot length, SFW, and RFW were measured after 3 d of Cu exposure. Root and leaf samples were dried at 80 °C to constant weight for determining SDW and RDW. Root length, root tip number, root surface area, root volume, and leaf surface area were measured using a scanner-based image analysis system (WinRHIZO; Regent Instruments, Quebec, Canada)^66^. Prior to analysis, roots were preserved in 70% ethanol.

### Determination of Cu Concentration

Root samples were collected and immerged in 25 mM EDTA-Na solution for 15 min to desorb metal ions on root surfaces. Next, root and leaf samples were washed thoroughly with tap water, rinsed with deionized water, cleaned with tissue paper, dried in an oven at 120 °C for 0.5 h to deactivate enzymes, and stored at 80 °C for 24 h. Next, these samples were ground to a fine powder, and 0.2 g was separately digested using an acid mixture of HNO_3_/HClO_4_ (87:13, v:v)^67^. The digests were dissolved in 5% HNO_3_ for Cu analysis using a NOVA 300 atomic absorption spectrophotometer (Analytik, Jena, Germany).

### Determination of TBARS Levels

Lipid peroxidation was determined by estimating the levels of TBARS as described by Jin *et al*.^37^. Briefly, 0.5 g of fresh root tissues was homogenized in a mortar with 5 mL of 0.25% 2-thiobarbituric acid and 10% trichloroacetic acid. The mixture was heated at 95 °C for 30 min, quickly cooled in an ice bath, and centrifuged at 10,000 × *g* for 10 min. The absorbance of the supernatant was measured at 532 nm and corrected for unspecific absorbance at 600 nm.

### Protein Extraction and Digestion

Root total proteins was extracted using a total protein extraction kit (Sigma-Aldrich, St. Louis, MO, USA), following the manufacturer’s instructions. Briefly, 250 mg of root tissue (10 plants pooled) was homogenized in liquid nitrogen. The homogenate was washed with methanol and acetone, and then, pelleted and dried with a SpeedVac (Thermo-Fisher Scientific, Waltham, MA, USA). The root tissue pellet was extracted with Type 4 Working Solution, containing 7 M urea, 2 M thiourea, 40 mM Trizma base, and 1% sodium dodecyl sulphate. After incubation for 15 min, the suspension was centrifuged at 14,000 × *g* for 30 min to remove the insoluble materials. The protein content in the supernatant was quantified using the Bradford assay (Bio-Rad, Hercules, CA, USA).

Protein samples (200 µg of bovine serum albumin equivalent) were digested using the filter-aided sample preparation method^68^. Briefly, the protein extracts were reduced by 10 mM dithiothreitol for 1 h at 56 °C, alkylated by 55 mM of iodoacetamide for 45 min at 25 °C in the dark, and buffer-exchanged with 100 mM NH_4_HCO_3_ (pH 8.5) using 10 KDa molecular weight cut-off Amicon Spin Tube (Millipore, Billerica, MA, USA). Subsequently, 4 µg of sequencing-grade modified trypsin (Promega, Madison, WA, USA) was added to each sample for protein digestion at 37 °C overnight (trypsin: protein, 1: 50). The digested peptides were desalted by Sep-Pak C18 cartridges (Waters, Milford, MA, USA) and quantified using a NanoDrop spectrophotometer (Thermo-Fisher Scientific).

### Conditions of Nano-UPLC-MS

For label-free relative quantification analysis, five biological replicates of each treatment group were analysed by an on-line nano-LC system (Thermo-Fisher Scientific) coupled with a linear trap quadrupole mass spectrometer (LTQ-Orbitrap; Thermo Scientific). The resulting peptides (1.5 μg) were acidified with 0.1% formic acid and subsequently loaded into the nano trap column (Acclaim PepMap100 C18; 75 μm × 2 cm, 3 μm, 100 Å; Thermo-Fisher Scientific) at a flow rate of 4 μL min^−1^ in a loading buffer, containing 2% acetonitrile and 0.1% formic acid in high performance liquid chromatography-grade water. Chromatographic separation was carried out using an analytical column (Acclaim PepMap RSLC C18; 75 μm × 15 cm, 3 μm, 100 Å; Thermo-Fisher Scientific) with a linear gradient of 3–55% Buffer B (80% acetonitrile and 0.1% FA) at a flow rate of 0.25 μl min^−1^ over 112 min. Due to loading and washing steps, the total time for an LC-MS/MS run was approximately 160 min.

One scanning cycle included an MS1 scan (m/z 300–1800) at a resolution of 60,000, followed by 10 MS2 scans by LTQ. The 10 most abundant precursor ions were fragmented at 35%. The lock mass calibration was activated, and the dynamic exclusion time was 30 s.

### Label-free Data Analysis

Raw MS files were processed by MaxQuant 1.5.2.5 employing the Andromeda algorithm and searched against the UniprotKB reference database for Viridiplantae (green plants) kingdom. In Andromeda search, the precursor and fragment ions mass tolerance was 6 ppm and 20 ppm, respectively. The maximum number of missed cleavages was two. The carbamidomethylation of cysteine was set as a fixed modification, with protein N-terminal oxidation of methionine as a variable modification. The false discovery rate (FDR) was set at 0.01. Protein abundances were calculated using the label-free quantitation algorithm^69^. Quantification was achieved using the label-free quantification (LFQ) with unique peptides. The match between runs option was enabled, allowing a time window of 2 min to search for already identified peptides in all obtained chromatograms. Protein abundance was calculated on the basis of the normalized spectral protein intensity (LFQ intensity), and proteins were quantified with a minimum of two ratio counts. The generated ‘proteingroups.txt’ table was filtered for contaminants, reverse hits, and number of unique peptides (≥1) using Perseus 1.5.3.2.

### Bioinformatics Studies of DAPs

DAPs were characterized proteins with an average fold change in abundance (Cu/Control) more than 1.5 and a *p* value less than 0.05. GO annotations were retrieved from a large number of references, whereas KEGG^70^ and PPI analysis were performed using Omicsbean (http://www.omicsbean.cn). The strengths of the PPI network relationships were visualized by assigning line weights to the compiled scores. PPI analysis was done with minimum required interaction score set to medium confidence 0.400^71^.

### Effect of Exogenous CA Application on *O. glazioviana* Seedlings Exposed to Cu

After 21 d in Hoagland’s nutrient solution, *O*. *glazioviana* seedlings were exposed to 50 μM Cu SO_4_ or 50 μM Cu SO_4_ and 50 μM CA for 3 d. Control plants were grown in Hoagland’s nutrient solution without Cu. FW, DW, and TBARS were determined as described above. Experiments were conducted in triplicate.

### Statistical Analysis

One-way analysis of variance (ANOVA) in conjunction with Duncan’s test was performed to identify significant differences (*p* < 0.05) between the groups using SPSS 19.0 (IBM, Armonk, NY, USA). All data were expressed as mean ± standard deviation.

## Electronic supplementary material

Supplementary data
