# Supplementary material for: Proteomic analysis on roots of Oenothera glazioviana under copper-stress conditions
Source: Sci Rep. 2017 Sep 6;7:10589. doi: 10.1038/s41598-017-10370-6 (PMC5587583; doi:10.1038/s41598-017-10370-6)
Supplement: Supplementary file 1 — Supplementary data [file 41598_2017_10370_MOESM1_ESM.pdf]

Proteomic analysis on roots of *Oenothera glazioviana* under copper-stress conditions

Chong Wang, Jie Wang, Xiao Wang, Yan Xia, Chen Chen, Zhenguo Shen, Yahua Chen*

Table S1. The information of total proteins by MaxQuant

Majority protein IDs	Peptide counts (unique)	Protein names	Gene names	Number of proteins	Unique peptides	Unique sequence coverage [%]	Mol. weight [kDa]	Sequence length	Score	LFQ intensity 50_R1	LFQ intensity 50_R2	LFQ intensity 50_R3	LFQ intensity 50_R4	LFQ intensity 50_R5	LFQ intensity CK_R1	LFQ intensity CK_R2	LFQ intensity CK_R3	LFQ intensity CK_R4	LFQ intensity CK_R5
A0MH68	1			1	1	2	54.742	493	6.9195	0	0	0	0	0	0	0	0	0	2222000
A2RVS6	1	Serine/arginine-rich splicing factor SR34A	SR34A	1	1	4	33.599	300	6.0638	0	0	0	0	0	0	0	0	1237900	0
A2XD35;Q10R17	1;1			2	1	4.1	52.008	487	35.21	847280	977790	842520	919560	802200	1591400	1341700	1612100	1649100	1512600
A5A7I8;Q9SZM3;Q38872;Q38871	1;1;1;1	Calcium-dependent protein kinase 26;Calcium-dependent protein kinase 6;Calcium-dependent protein kinase 5	CPK26;CPK6;CPK5	4	1	3.6	59.994	535	6.2146	0	0	0	0	0	0	0	0	0	1419100
A5B8T3	2	Putative uncharacterized protein	VIT_05s0102g00710	1	1	20	35.199	330	24.65	1336700	1486600	1331500	1311100	1407300	3150200	2022700	1915000	1946100	1867800
A6YGB8;P07753;Q1KVU8;P09752	1;1;1;1			4	1	3.2	38.149	344	7.7895	0	0	0	0	2838300	0	0	0	0	0
A7PZL3	2			1	2	6.3	53.489	491	15.442	2291000	2080700	2082000	1918100	3084000	1456200	1348000	1287100	1305400	1346700
A8MRZ7	1	Eukaryotic initiation factor 4A-1	EIF4A1	1	3	17.3	41.85	369	34.59	2041100	2660800	1381900	1857400	1855700	3513600	3496000	3501600	2866400	2802100
A8SEG5	1			1	1	0.9	225.21	1899	5.9088	0	0	0	0	0	0	0	0	0	4980600
B2XWJ8	1			1	1	12	9.9195	83	6.3083	0	0	0	0	9512100	0	0	0	0	0
B6T451	1	Importin subunit alpha	N/A	1	3	19.9	57.879	527	38.58	914240	826100	926750	893010	860130	1341100	2134900	1343000	1516600	1403200
B7EA73	1			1	1	1.2	99.523	887	6.0351	0	0	0	0	0	0	0	0	0	208870
B7SDI4	1	Aquaporin	N/A	1	2	21.5	8.8289	79	18.668	2318400	2113200	2359900	2467900	2261400	3598100	3606400	3865500	4270200	3689300

B8B9K6;<br>B7F845	1;1			2	1	6.5	24.483	216	7.2574	0	0	0	0	0	0	0	0	0	181850
B8BBN7;<br>Q9SA73;<br>Q6Z1J6	2;1;1	Obg-like ATPase 1	T5I8.3	3	2	5.8	44.346	394	11.553	7262600	6085200	6558500	6412400	7434600	3894900	3709800	4088400	3549800	0
B9DFG5	1	PTI1-like<br>tyrosine-protein<br>kinase 3	PTI13	1	1	2.7	45.658	408	7.7524	0	0	0	0	0	1855100	0	0	0	0
B9DGD6	1	Acetyl-coenzyme A<br>synthetase,<br>chloroplastic/glyox<br>ysomal	ACS	1	1	2	81.888	743	7.3552	0	0	0	0	0	0	0	0	0	998010
B9HBA8	1;0;0			3	1	1.7	57.707	528	51.258	0	0	0	0	0	0	0	2422600	0	0
B9N843	1			1	1	4.6	57.496	526	6.0534	0	0	0	0	0	0	0	0	0	1137800
B9RT61	1	Translationally-con<br>trolled tumor<br>protein homolog	TCTP	1	2	27.4	18.962	168	98.63	1595200	2243300	1705700	1487400	1768700	5331400	5017100	4639900	4285200	3887400
B9SL58;<br>Q96529	1;1	Adenylosuccinate<br>synthetase,<br>chloroplastic	PURA	2	1	2.9	53.303	488	6.8291	0	0	0	0	0	0	0	0	0	620810
B9T118	1	NADH-ubiquinone<br>oxidoreductase,<br>putative	RCO<br>M_04<br>58390	1	3	5.5	80.768	744	89.76	778470	695050	707620	756700	705380	1218000	1438000	995690	1075600	1363000
C5WWY<br>0;A2Z7C<br>4;A1L4T<br>4	1;1;1			3	1	5.5	21.489	181	6.9195	0	0	0	0	0	0	0	0	1713600	0
C6TBN2	3			1	3	8.1	38.252	346	20.461	1034400<br>0	1037400<br>0	8581600	1061300<br>0	9305500	4656400	4600700	6089900	5141500	4524800
C6TEX6	1			1	1	4.2	42.913	382	6.0056	0	0	0	0	0	0	0	0	0	302510
D7LSV8	1	ADP-ribosylation<br>factor	ARAL<br>YDRA<br>FT_48<br>6735	1	9	78	21.585	189	22.526	2097300<br>0	2449300<br>0	2353900<br>0	2330200<br>0	2181600<br>0	4224800<br>0	4149400<br>0	4958900<br>0	4820900<br>0	4603400<br>0
D7SW76	1	Uncharacterized<br>protein	VIT_0<br>7s003<br>1g017<br>40	1	2	12.5	27.837	240	182.70<br>4	1389800	1024000	1209800	1249700	991190	1712500	1602200	2206900	2026700	2001800
D7UC38;<br>A5BPL1	2	Phosphomannomut<br>ase	VIT_1<br>5s004<br>6g035<br>20	2	1	5.6	28.114	249	36.63	1501000	1587400	1334300	1300900	1389300	664990	745090	577410	642260	757130

E0CSI1	1			1	1	2.3	57.093	517	7.0319	0	0	0	0	0	0	0	0	0	570700
E1U332	1			1	1	3.9	34.068	308	5.9066	0	0	0	0	0	0	0	0	0	1973900
E9L7A5; Q5F4K8	1;1			2	1	3.5	51.743	479	10.649	0	0	0	0	0	0	0	0	0	728110
F4IGA5	1	Nuclear pore complex protein NUP133	NUP133	1	1	1.9	144.6	1285	5.8798	0	0	0	0	0	0	0	0	0	3459100
F4IUG9	1	Myosin-13	XI-G	1	1	0.5	169.07	1493	5.8392	0	0	0	0	590590	0	0	0	0	0
F4IUX6	2	Regulator of nonsense transcripts UPF2	UPF2	1	2	2.6	133.96	1181	10.937	3451700	0	0	0	0	0	3693000	0	0	0
F4JFN3	1;0		Hsp89.1	2	1	1.8	90.566	799	13.191	0	0	0	0	0	0	0	0	0	755870
F4JLM5	1		At4g10320	1	1	0.8	135.48	1190	6.8543	0	0	0	0	0	0	0	0	1194800	0
F4JLP5; A8MS68	2;1	Dihydrolipoyl dehydrogenase 2, chloroplastic;Dihydrolipoyl dehydrogenase 1, chloroplastic	LPD2; LPD1	2	2	5.3	60.144	567	13.704	3392900	2559700	2421300	2770600	2781500	1936200	1678200	1734400	0	0
F6H7K5; Q38814; P84548; Q38709; O23787	2;2;1;1;1	Thiamine thiazole synthase, chloroplastic	THI1	5	2	7.3	38.104	355	18.086	0	0	0	0	0	2032900	2233800	2009100	2246000	2156000
G7IRR6	1	Protein disulfide-isomerase	MTR_2g094180	1	2	4.6	63.018	564	102.49	1846200	1914000	1931400	1734000	1754500	2555700	2669800	2632600	2536400	3898500
J7KE88	1	Heat shock protein 90	HSP90	1	2	19.3	80.271	699	175.2	6862900	5518300	6082200	6132900	4478900	3510000	4144600	3687500	3455600	3908800
M0TRQ8	1	Succinyl-CoA ligase subunit beta	N/A	1	2	8.6	45.009	421	13.45	613720	583000	592160	555610	592130	820130	1351800	784120	806490	785880
M1CZC0	1			1	1	2.3	42.74	387	9.0876	0	0	4866300	0	0	0	0	0	0	0
O03986	2	Heat shock protein 90-4	HSP90-4	1	2	3	80.14	699	47.385	0	0	0	0	0	1981600	2992400	2087600	2237700	1443100
O04121	1			1	1	3.2	49.214	439	6.0343	0	0	0	0	0	0	0	0	0	146870
O04151; Q40401; O81919;	3;1;1;1;									2339700	2489200	2057600	1947600	2308600	2783100	2711300	2711800	2593400	2903700
Q9XF98	0;0;0	Calreticulin-1	CRT1	7	3	13.4	48.527	425	121.8	0	0	0	0	0	0	0	0	0	0
O04153	1	Calreticulin-3	CRT3	1	1	3.1	49.843	424	6.911	0	0	0	0	0	0	0	0	0	305910

O04226; O04015; O65361; P54887;P54888;P32296;Q96480	2;2;2;2;2;1;1	Delta-1-pyrroline-5-carboxylate synthase A;Glutamate 5-kinase;Gamma-glutamyl phosphate reductase;Delta-1-pyrroline-5-carboxylate synthase B;Glutamate 5-kinase;Gamma-glutamyl phosphate reductase	P5CSA;P5CSB	7	2	3.2	77.744	716	13.263	1065100	1165100	1170500	1202000	1044300	1307600	1286500	1300100	1161100	1464200
O04267; O04266; Q01474; O04834	2;1;1;1;0	GTP-binding protein SAR1B;GTP-binding protein SAR1A	SAR1B;SAR1A	5	2	19	22.077	195	77.73	3653000	5502700	3148900	3379400	5292200	4681800	4489400	4385600	3419000	0
O04308; P29677; Q9ZU25	2;1;1	Probable mitochondrial-processing peptidase subunit alpha-2;Probable mitochondrial-processing peptidase subunit alpha-1	MPPA2	3	2	5	54.053	499	15.034	3779300	4206400	4547200	4817200	5600900	1785300<br>0	1761400<br>0	1505200<br>0	1538300<br>0	1545300<br>0
O04397; Q41014; P41345; O23877; Q9M0V6;Q9S9P8	1;1;1;1;1;1	Ferredoxin--NADP reductase, root isozyme 1, chloroplastic;Ferredoxin--NADP reductase, root isozyme 2, chloroplastic	RFNR1;RFNR2	6	1	2.7	41.957	375	6.0795	0	0	0	0	0	0	0	0	979120	0
O04499; O24246; P30792	1;0;1;0	2,3-bisphosphoglycerate-independent phosphoglycerate mutase 1	PGM1	4	1	1.8	60.579	557	6.7368	0	0	0	0	0	0	0	0	0	7754500
O04885; Q8H0V3	1;1	Lactoylglutathione lyase		2	1	7	20.783	185	11.608	0	0	0	0	0	0	0	0	0	1481400
O04887	2			1	2	4.1	56.327	510	12.167	7607700	8319900	8141800	7754100	7187300	0	0	0	0	0
O04899; Q9SWB8	1;1;1;1;0;0;0;0;	Caffeoyl-CoA O-methyltransferas	CCOAOMT1	9	1	9.2	27.142	240	43.567	0	0	0	0	0	0	0	0	0	1582400

;O49499	0	e 1																	
O04937	1			1	1	2.9	44.796	411	6.5943	0	0	0	0	748270	0	0	0	0	0
O04973;O04974	1;1			2	1	2.4	64.36	589	5.9997	0	0	0	0	0	0	0	0	0	444610
O22862;Q9FLV5	1;1	Probable peroxidase 26;Probable peroxidase 61	PER26;PER61	2	1	4.8	37.568	335	7.9605	0	0	0	0	0	0	0	0	0	440590
O23140	1	AP-2 complex subunit mu	AP2M	1	1	2.3	49.322	438	6.7728	0	0	0	0	0	0	0	0	641640	0
O23254	2	Serine hydroxymethyltransferase 4	SHM4	1	2	6.4	51.717	471	12.947	16618000	16134000	14455000	16772000	0	13540000	13269000	14054000	0	12819000
O23627	1	Glycine--tRNA ligase 1, mitochondrial	GLYRS-1	1	1	2.1	81.943	729	6.7698	0	0	0	0	0	2261500	0	0	0	0
O23654;Q39291;Q39442	1;0;0	V-type proton ATPase catalytic subunit A	VHA-A	3	1	1.4	68.812	623	125.5	0	0	0	0	0	0	0	0	0	6204000
O23657	4	Ras-related protein RABC1	RABC1	1	4	22.6	23.53	212	30.046	4342700	4794000	4528100	4626800	4539600	3914700	3804300	3533400	3776100	4063000
O23712;P34066	1;1	Proteasome subunit alpha type-1-B;Proteasome subunit alpha type-1-A	PAF2;PAF1	2	1	6.1	30.41	277	7.3101	0	0	0	0	0	0	0	0	0	3983000
O23755	12			1	12	20.8	93.798	843	216.29	39741000	35029000	34341000	33576000	32423000	46900000	51376000	52350000	47740000	49121000
O23760	1			1	1	4.6	40.261	370	12.677	0	0	0	0	0	0	0	0	0	1210000
O24111;Q9ZUT9;P51427	1;1;1	40S ribosomal protein S5-1;40S ribosomal protein S5-2	RPS5A;RPS5B	3	1	9.7	17.101	154	51.451	22729000	23651000	19397000	19184000	20018000	27683000	25550000	23297000	27105000	23068000
O24144;O24151	2;2			2	2	10.9	26.965	239	36.655	0	0	0	0	0	0	0	0	3953000	0
O24310	1			1	1	3.3	52.938	488	7.8912	0	0	0	0	0	0	0	0	0	875590
O24364;Q96291	2;2	2-Cys peroxiredoxin BAS1, chloroplastic	BAS1	2	2	6.8	28.895	265	23.18	0	3968600	3457200	3549100	3895000	5454900	6161900	5621200	5698400	4709700
O24456	1	Receptor for activated C kinase	RACK1A	1	1	5.2	35.747	327	15.629	0	0	0	0	0	0	0	0	0	10409000

		1A																	
O24466;<br>Q39433;<br>P28186	2;1;1	Ras-related protein RABE1a;Ras-related protein RABE1c	RABE1A;RABE1C	3	2	10.2	23.939	216	55.922	6199300	5144900	6848200	8556400	6534200	0	0	0	4493800	7071200
O48660;<br>O48658;<br>Q96557;<br>Q9ZS45;<br>Q96556;<br>O48659;<br>O82147;<br>Q9SMB1;Q9ZTR0;Q9ZUB3;O48661	1;1;1;1;0;0;0;0;0;0;0	Spermidine synthase 1;Spermidine synthase 2	SPDSYN1;SPDSYN2	11	1	4.1	34.512	314	16.93	1879200	2048400	1639000	1842700	0	4030400	3864700	0	3835900	3506500
O48788	1	Probable inactive receptor kinase At2g26730		1	1	1.8	71.751	658	7.2413	0	0	0	0	0	0	0	0	242480	0
O48905;<br>Q7XDC8;Q08062;<br>O24047	1;0;0;0			4	1	9.3	35.546	332	22.52	5593100	0	5545900	4604000	4485700	2326800<br>0	3218100<br>0	3408800<br>0	3412900<br>0	1933700<br>0
O49046	1			1	1	2.6	38.61	350	12.08	0	0	0	0	0	0	0	0	0	602280
O49377	1	Vesicle-associated membrane protein 711	VAMP711	1	1	5.5	25.039	219	7.1192	0	0	0	0	0	0	0	0	0	520530
O49484	1	SKP1-like protein 11	ASK11	1	1	9.9	17.288	152	41.463	0	0	0	0	0	0	0	0	0	7295300
O49485	3;1	D-3-phosphoglycerate dehydrogenase 1, chloroplastic	PGDH1	2	3	4.3	63.324	603	24.901	1068700	1129000	1129800	1095400	1185100	2227000	5370500	2122800	2173700	2107300
O49818	1			1	1	7.5	21.087	186	5.9273	0	0	0	0	0	0	0	0	0	455440
O49845;<br>P49035;P49037;P49039;P10691;Q9LXL5	2;2;1;1;0;1;0;0;0;0	Sucrose synthase 4	SUS4	10	2	2.6	91.649	801	29.252	5574700	4736700	5926000	6621600	4542700	2323200	2410800	2367000	0	0
O49886	1			1	1	7	18.287	172	7.041	0	0	0	0	0	0	0	0	2128700	0
O64459	1			1	1	3.8	51.845	471	16.725	4225000	4302400	4311000	4205300	3537700	6155400	5784000	6207300	8851300	5722700
O64530	1	Thiosulfate/3-mercaptopyruvate	STR1	1	1	2.6	41.893	379	6.7951	0	0	0	0	0	0	0	0	0	2978500

		sulfurtransferase 1, mitochondrial																	
O64650;Q9M2F1;Q96564	2;2;1	40S ribosomal protein S27-1;40S ribosomal protein S27-2	RPS27A;RPS27B	3	2	21.4	9.405	84	16.264	1738000	1923600	1653500	1797400	1863300	0	0	0	0	0
O64700;Q6AUF3;E0ZS47	3;2;2	Urease accessory protein G	UREG	3	3	18.5	30.082	275	37.784	0	0	0	0	0	0	0	0	0	684180
O64982;Q41365;Q9SSB5	3;2;2;1	26S protease regulatory subunit 7 homolog A	RPT1A	4	3	10.6	47.557	425	37.29	0	3022000	1944900	3529100	2023700	3473500	3817300	3866900	3671600	3015700
O65084;O81153	1;0	Proteasome subunit beta type-3-B	PBC2	2	1	14.7	22.908	204	34.709	0	0	0	0	0	0	0	0	0	3309000
O65175;P93407	2;1			2	2	13	22.075	216	18.144	0	7216900	10607000	6589200	7585200	6786500	9294600	0	0	0
O65316;O65315;O65314	1;0;0			3	1	4.2	41.478	377	15.373	0	0	0	0	0	0	0	0	0	832760
O65729;Q940B0;P42791	1;1;1	60S ribosomal protein L18-3;60S ribosomal protein L18-2	RPL18C;RPL18B	3	1	7.7	20.643	183	15.301	7438700	12673000	7667300	6946700	7179900	4088000	3844100	4196100	4030100	4284500
O65731	1			1	1	6.6	22.018	197	8.1057	0	0	0	0	0	4919900	5177700	5038000	4700300	4942800
O65781	2			1	2	8.3	38.373	350	48.685	6231200	7274500	8218000	6080000	6441800	4853500	5424400	5535500	5458900	5398700
O65806;P49030	1;1			2	1	10.1	17.555	149	7.2794	0	0	0	0	0	0	0	0	0	908540
O65819;Q1SU99;P93354;O22582;Q1SWQ1;Q1S9I9;Q9LZ45;Q9ZUS0;Q9LZT0;Q9FFC0;Q9LQQ4;P40283;O23629;Q9SI96;Q99285;	2;2;2;2;2;2;2;2;2;2;2;2;2;2;1;1;1;1;1;1;1;1;1;1;1;1;1;1;1;1;1;1;1;1;1;1;1;1;1;1;1;1;1;1;1;1;1;1;1;1;1;1;1;1;1;1;1;1;1;1;1;1	Histone H2B.9;Histone H2B.4;Histone H2B.7;Histone H2B.10;Histone H2B.1;Histone H2B.11;Histone H2B.6;Histone H2B.3;Histone H2B.5;Histone H2B.8	H2B	58	2	17.5	15.045	137	27.455	2.5E+08	2.55E+08	2.32E+08	2.22E+08	2.63E+08	1.38E+08	0	0	0	2.02E+08

Q43215;
Q43216;
P49120;
Q43217;
Q943L2;
A2WWU
2;O65818
;O65821;
O49118;
Q6ZBP3;
P30756;P
05621;A2
YWI3;P3
0755;Q6
F362;P27
807;A3A
GM4;A2
Y7R3;A2
XF66;Q9
LGI2;Q9
LGH8;Q
9LGH4;
Q94JJ7;Q
94JJ4;Q7
GBK0;Q
43261;P5
4347;P54
346;P505
65;A2W
KT1;A2
WKS8;A
2WKS5;
A2WKS3
;A2WKP
5;A2WK
P3;P5434
8;Q94JE1
;P16868;
A2WKT4
;P54345;
P16867;
Q9SF55;

Q9LFF6																			
O80501	2	Ras-related protein RABH1b	RABH1B	1	2	12.5	23.13	208	63.748	5915400	3625600	4657200	3928300	4601900	12624000	12066000	12734000	11425000	10246000
O80526;Q9SGY2	2;1	ATP-citrate synthase alpha chain protein 3;ATP-citrate synthase alpha chain protein 1	ACLA-3;ACLA-1	2	2	8.5	46.945	424	13.665	0	1184000	937670	975680	846090	9805800	2211000	0	2177700	0
O80585	1	Methylenetetrahydrofolate reductase 2	MTHFR2	1	1	2.5	66.801	594	6.1349	0	0	0	0	0	0	0	0	0	1524700
O80840	2	Phosphomannomutase	PMM	1	2	10.2	27.761	246	11.683	0	0	0	0	0	3705400	0	0	0	4142400
O81361;Q9FIF3	2;1	40S ribosomal protein S8-2	RPS8B	2	2	13.6	24.721	221	37.97	42368000	41795000	39853000	48482000	38660000	17673000	17724000	18474000	15515000	19488000
O81742;Q9SUS3	3;3	Beta-adaptin-like protein C;Beta-adaptin-like protein B	BETAC-AD;BETAB-AD	2	3	5.2	99.096	893	38.395	1783300	1742600	0	0	0	2610400	2474300	2368400	2271900	2327000
O81920;Q9M060	1;1	Eukaryotic translation initiation factor 6-2	EIF6-2	2	1	7.3	20.863	193	6.1225	0	0	0	0	0	0	0	0	0	1390500
O81982	1			1	1	9.8	14.285	133	9.4279	6732100	6886300	6884200	0	5998000	11084000	11424000	16382000	11689000	0
O82229	1	Putative serine carboxypeptidase-like 23	SCPL23	1	1	4	51.528	454	10.734	0	0	0	0	0	0	0	0	0	828070
O82528	1			1	1	6.9	24.099	204	14.764	0	0	0	0	0	0	0	0	0	4200000
O82530;P52427	1;1			2	1	8.8	27.232	249	43.682	1382200	1349900	1247600	0	1555700	5414700	3308700	3156300	2981000	2798300
O82663;Q6ZDY8;Q9ZPX5	6;5;4	Succinate dehydrogenase [ubiquinone] flavoprotein subunit 1, mitochondrial;Succinate dehydrogenase [ubiquinone] flavoprotein subunit 2, mitochondrial	SDH1-1;SDH1-2	3	6	17.2	69.656	634	108.94	6545900	7364900	7910900	6078200	7860700	12111000	8838500	7195600	12071000	14533000

O82734	1	Serine/threonine-protein phosphatase PP1 isozyme 8	TOPP8	1	1	2.5	36.806	324	6.0643	0	0	0	0	0	0	0	0	0	2429200
P00784	1			1	1	4.1	38.921	345	7.054	0	0	0	0	0	0	0	0	0	8834900
P00785;A5HII1	1;1			2	1	5	42.172	380	6.5959	0	0	0	0	0	0	0	0	0	3261800
P02582	1			1	1	4.8	41.617	375	39.217	0	0	0	0	0	0	0	0	805040	0
P04464	2			1	2	10.7	16.847	149	323.31	1.07E+08	1.11E+08	1.18E+08	1.07E+08	1E+08	83228000	70077000	76398000	98497000	89192000
P04783;P84990;P15192;P12332;P08222;P04159;P14273;P08221;P09755;P12331;P12329;Q10HD0;P09756;A2XJ35;P92919;P08963;P27520;P27518;P27497;P27494;P27493;P15195;P14278;P12330;P07370;P06671;P27492;P13851;P12470;P10049;P04782;P04779;P27496;P27495;P27491;P20866;P12469;P12	2;1;1;1;1;1;1;1;1;1;1;1;1;1;1;1;1;1;1;1;1;1;1;1;1;1;1;1;1;1;1;1;1;1;1;1;1;1;1;1;1;1;1;1;1;1;1;1	Chlorophyll a-b binding protein 3, chloroplastic;Chlorophyll a-b binding protein 2, chloroplastic;Chlorophyll a-b binding protein 1, chloroplastic	LHCB1.2;LHCB1.1;LHCB1.3	48	2	16.1	28.383	267	12.74	8986000	9359300	9849000	9288400	17248000	3097400	3208700	0	0	0

333;P07369;P04781;P27490;P07371;P15194;P24006;P15193;Q8VZ87;P0CJ48;P04778																			
P05492;Q01915;P05493;P24459;Q06735;P05494;P18260	5;3;3;2;2;0;1;1;0;0;0;0;0;0;0;0			16	5	14.1	55.596	511	323.31	1.31E+08	1.32E+08	1.16E+08	1.23E+08	1.6E+08	1.64E+08	1.45E+08	1.64E+08	1.59E+08	1.4E+08
P05522;Q652F9;Q8LQ92;Q6Z2J3;Q69NF5;Q9SZ90;Q9SUS0;O49296;Q9CAC1;Q8GY58;Q9SVJ4;Q93YQ7;Q9SRX3;O81416	1;1;1;1;1;1;1;1;1;1;1;1;1;1	Endoglucanase 18;Endoglucanase 20;Endoglucanase 4;Endoglucanase 8;Endoglucanase 23;Endoglucanase 22;Endoglucanase 24;Endoglucanase 1;Endoglucanase 17	CEL1;GH9B16;CEL2	14	1	1.8	54.079	494	7.213	0	0	0	0	0	0	0	0	253860	0
P06291	1			1	1	8.7	21.081	184	6.0553	0	0	0	0	0	0	0	0	0	73283000
P07803;P68169;P68168;Q9FVL0;P23244;Q42831;Q947C5;Q94FT7;Q94FT8;O049	2;2;2;1;1;1;1;1;1;1;1	Non-symbiotic hemoglobin 1	AHB1	11	2	12.4	18.15	161	12.144	3840000	0	0	0	0	0	14815000	0	15746000	0

85;O24520																			
P08282;P32289;P04078	1;1;1			3	1	4.5	38.927	355	17.184	0	0	0	0	0	0	0	0	0	1020600
P08440;P29356	1;1			2	1	3.4	38.604	355	10.18	31378000	32953000	36457000	28923000	31515000	20174000	18906000	18518000	20776000	19415000
P08746	2			1	2	6.9	18.202	159	13.94	0	3377800	8134400	3038300	0	1386100	1382000	1475800	0	1424800
P08824	5;2			2	5	10.9	52.378	495	64.42	3967900	3890500	3606500	3839600	4939300	5732700	6561600	5585600	5713200	5751900
P08927;C0Z361;Q9LJE4;P21240;P21241	10;8;6;6;5;4;1	Chaperonin 60 subunit beta 3, chloroplastic;Chaperonin 60 subunit beta 2, chloroplastic;Chaperonin 60 subunit beta 1, chloroplastic	CPN60B3;CPN60B2;CPN60B1	7	10	25.5	62.983	595	312.17	29566000	27788000	26108000	26223000	28660000	32451000	37490000	38486000	37187000	38032000
P09003; Q04613	1;1	ATP synthase protein MI25	AtMg00640	2	1	6.6	22.436	198	6.3477	0	0	0	0	0	0	0	0	0	904540
P09204;P09205	1;1			2	1	4.7	49.586	451	16.883	0	0	0	0	0	0	0	0	0	1610300
P0C073; P0C031; P0C030; Q8RUC6;Q9SHE7;P69310; P69326;P69317;P69313;P51423;P49636;P0CH35;P0CH34;P59272;Q9ARZ9;P51431;P47905; P27923;P0CG87;P0CG86;P62981;P6	2;2;2;2;2;1;1;1;1;1;1;1;1;1;1;1;1;1;1;1;1;1;1;1;1;1;1;1;1;1;1;1;1;1;1;1;1;1;1;1;1	Ubiquitin-NEDD8-like protein RUB2;Ubiquitin;NEDD8-like protein RUB2;Ubiquitin-NEDD8-like protein RUB1;Ubiquitin;NEDD8-like protein RUB1;Ubiquitin-60S ribosomal protein L40-2;Ubiquitin;60S ribosomal protein L40-2;Ubiquitin-60S ribosomal protein L40-1;Ubiquitin;60S ribosomal protein L40-1;Ubiquitin-40S ribosomal protein S27a-1;Ubiquitin;40S ribosomal	RUB2; RUB1; RPL40B;RPL40A;RPS27AA;RPS27AC;RPS27AB;UBQ11; UBQ12;UBQ14;UBQ3; UBQ9;UBQ4;UBQ10;UBQ8	45	2	19.6	17.115	153	47.392	28035000	28715000	29256000	25669000	46008000	29251000	25905000	24200000	26104000	26468000

2980;P0C	protein
G83;P69	S27a-1;Ubiquitin-4
325;P693	0S ribosomal
15;P6930	protein
9;P0CG8	S27a-3;Ubiquitin;4
4;Q58G8	0S ribosomal
7;P69322	protein
;P0CG85;	S27a-3;Ubiquitin-4
P0CH05;	0S ribosomal
P0CH04;	protein
Q42202;	S27a-2;Ubiquitin;4
B9DHA6	0S ribosomal
;P59271;	protein
P59233;P	S27a-2;Polyubiquit
59232;P0	in
CH33;Q3	11;Ubiquitin;Polyu
E7K8;Q3	biquitin
E7T8;Q1	12;Ubiquitin-relate
EC66;Q9	d
FHQ6;P0	1;Ubiquitin-related
CH32;Q8	2;Ubiquitin-related
H159;Q3	3;Polyubiquitin
9256	14;Ubiquitin;Polyu
	biquitin
	3;Ubiquitin;Polyubi
	quitin
	9;Ubiquitin-related
	1;Ubiquitin-related
	2;Ubiquitin-related
	3;Ubiquitin-related
	4;Polyubiquitin
	4;Ubiquitin;Polyubi
	quitin
	10;Ubiquitin;Polyu
	biquitin
	8;Ubiquitin-related
	1;Ubiquitin-related
	2;Ubiquitin-related
	3;Ubiquitin-related
	4;Ubiquitin-related
	5;Ubiquitin-related
	6;Ubiquitin-related

		7;Ubiquitin-related 8																	
P0C522;P0C521;P0C520	1;1;1			3	1	1.8	55.373	509	7.5918	0	0	0	0	0	15988000	0	0	0	0
P10896	1	Ribulose bisphosphate carboxylase/oxygenase activase, chloroplastic	RCA	1	1	3.2	51.981	474	7.4822	0	0	0	0	417680	0	0	0	0	0
P11143	1			1	1	1.9	70.461	645	43.872	0	0	0	0	0	0	0	0	0	10193000
P11428;Q9SQL5;Q8L5E0;Q7M1R5;Q42612;Q42611;Q0DRV6;Q07796;Q02610;P28757;P27082;P23346;P23345;P22233;P09678;O22373;A2XGP6;O04996;P24704	1;1;1;1;1;1;1;1;1;1;1;1;1;1;1;1;1;1;1	Superoxide dismutase [Cu-Zn] 1	CSD1	19	1	6.6	15.104	151	6.5765	0	0	0	0	0	0	0	0	0	34877000
P11574;Q8W4E2	1;1	V-type proton ATPase subunit B1;V-type proton ATPase subunit B3	VHA-B1;VHA-B3	2	1	2.5	54.11	486	9.7744	18800000	18232000	16731000	16808000	15701000	9831400	9281000	9382600	10644000	8106700
P12411;P46263;P46264	3;3;3	Tubulin beta-1 chain	TUBB1	3	3	12.1	50.217	447	97.624	0	0	0	0	0	22066000	22195000	24302000	22401000	21223000
P12460	1			1	1	3.1	50.627	449	7.9753	0	0	0	0	0	0	0	3505500	3675500	3785500
P15102	2			1	2	5.6	47.246	429	252.28	1004900	934110	648250	978710	753380	11976000	12300000	4188400	10499000	10647000
P15590	1			1	1	1.7	65.028	573	6.0534	0	0	0	0	0	0	0	0	0	116460
P17094;P	1;0	60S ribosomal	ARP1;	2	1	3.9	44.559	389	18.271	0	0	0	599650	0	0	0	0	0	0

22738		protein L3-1;60S ribosomal protein L3-2	ARP2																
P17202;P28237;Q84LK3;B3VMC0;Q9S795	1;1;1;1;1	Betaine aldehyde dehydrogenase 1, chloroplastic	ALDH10A8	5	1	2.2	54.27	497	34.59	0	0	0	0	1371700	0	0	0	0	0
P17614	1;0;0;0;0;0;0;0;0;0			10	1	2.9	59.855	560	323.31	53949000	50914000	56023000	48446000	52512000	3964700	8099500	13754000	21041000	29048000
P17783;P85920;P86074;Q43744;Q9ZP06;Q9LKA3	1;0;0;0;0;0;0	Malate dehydrogenase 1, mitochondrial;Malate dehydrogenase 2, mitochondrial		8	1	6.3	36.2	347	44.652	0	0	0	0	0	0	0	0	0	5197700
P18026;Q76FS3;Q9ZRB2;Q43697;Q41785;Q9ZPN7;Q41783;Q9ZRA8;Q43594;P93176;P45960;Q9ZPN9;Q9ZRA9;Q43695;Q41782;Q9ZRB1;Q39445;P33631;P22852;P11482;P04690;O04386;Q39697	1;0;0;0;0;0;0;0;0;0;0;0;0;0;0;0;0;0;0;0;0;0;0			25	1	3.4	49.883	444	29.895	0	0	0	0	0	0	0	0	2968400	0
P19456;P20649	2;2	ATPase 2, plasma membrane-type;ATPase 1, plasma membrane-type	AHA2;AHA1	2	2	2.4	104.4	948	30.507	0	0	0	0	0	9469000	10844000	9998600	9500200	9393600

P21239;P34794	2;1;0			3	2	5.9	57.691	546	93.989	1861800	1922600	4678200	1778400	2033100	21751000	23663000	19683000	22480000	17569000
P21357;P37216;Q00218	2;2;1	Phospho-2-dehydro-3-deoxyheptonate aldolase 2, chloroplastic	DHS2	3	2	4.6	59.667	538	12.685	0	0	0	0	2879500	411980	440150	451880	445090	385750
P21820;P48494;P46226;P34937;P12863	2;1;1;1;1;0;0;0;0			10	2	10.7	27.09	253	56.209	63457000	49421000	1.03E+08	1.76E+08	1.53E+08	26496000	20403000	23402000	41521000	47340000
P22200	1;0			2	1	3.7	55.17	510	21.388	4780000	4430800	4424200	3680300	3338200	4466400	4991800	4579600	4359100	4215700
P22337;Q96456;P22243;Q42807	1;1;1;1;1;0			6	1	4.3	45.37	396	29.859	0	0	0	0	0	0	0	0	0	856710
P23712	3			1	3	6.7	39.47	358	125.51	8927200	9399400	8876100	0	7450900	42175000	17232000	18879000	28664000	17875000
P24067;O24581	1;1;0			3	1	2	73.084	663	8.1429	0	0	0	0	0	0	0	0	0	17382000
P24715	1			1	1	11.1	8.9173	81	6.1873	0	0	0	0	0	0	0	0	2222600	0
P24922;Q9AXQ6;P69040;P69039;P56337;Q9AXQ3;P56333	2;0;0;0;0;0;0;0			8	2	10.1	17.363	159	131.22	12001000	12524000	8265200	9867600	9023800	15398000	14903000	14490000	14139000	13222000
P25083;P40941	1;1	ADP,ATP carrier protein 2, mitochondrial	AAC2	2	1	2.8	42.058	386	21.363	0	0	0	0	0	0	0	0	0	7397400
P25696	1	Bifunctional enolase 2/transcriptional activator	ENO2	1	1	2.5	47.719	444	14.259	0	0	0	0	0	0	0	0	0	1967700
P26301	1			1	1	4	48.063	446	323.31	0	0	0	0	0	0	0	0	0	430870
P26360	1			1	1	5.8	35.575	326	62.824	0	0	0	0	0	0	0	0	0	1774300
P26413	1			1	1	4	70.879	645	145.89	8950900	0	0	0	0	42387000	0	0	0	0
P26517;Q09054;P08735;P	1;0;0;1;0;0;0;0;0;0;0;0			12	1	8	36.513	337	227.81	0	0	0	0	0	0	0	0	0	6864300

26520;P08477;Q43247;Q0J8A4;P25861;A2YQT7;P34922;P34921;P04796																			
P26563	1			1	1	2.2	49.915	454	6.0884	0	0	0	0	0	0	0	0	0	1849600
P27322	2;2			2	2	2.2	70.706	644	323.31	1086600<br>0	1083100<br>0	1155100<br>0	1048600<br>0	9565000	2386900<br>0	2010600<br>0	0	2345300<br>0	2326400<br>0
P27323	1	Heat shock protein 90-1	HSP90-1	1	1	2.7	80.635	700	38.919	1793000	0	1719600	0	1863300	5148700	4522600	6446500	8603500	4158100
P27489	1			1	1	3.8	28.622	265	6.0423	0	0	0	0	1047200	0	0	0	0	0
P27583	1			1	1	17.9	10.972	106	14.637	0	0	0	0	0	0	0	0	0	1038800
P27608	1			1	1	2.8	59.974	542	7.7659	0	0	6247400	2013600	0	924930	990040	934860	1018700	949060
P28011	1			1	1	3.6	45.691	418	16.873	0	0	0	0	0	0	0	0	0	990190
P28188	2;0;0;0;0;0;1;0;0	Ras-related protein RABD2a	RABD2A	9	2	11.8	22.648	203	12.902	7536600	6726600	1201500<br>0	9116000	9332800	0	0	0	0	5435400
P28644;P19683;Q9FGS0	2;1;1	RNA-binding protein CP31B, chloroplastic	CP31B	3	2	12.4	25.231	233	18.19	1921000	1733900	1332200	1484300	1768300	2017200	1960500	2071900	0	2562300
P28723;Q9SPK5	2;1	Formate--tetrahydrofolate ligase	THFS	2	2	8.3	67.854	637	12.918	0	4513300	3661800	0	4715200	0	0	3947800	3843100	4075900
P28769	4	T-complex protein 1 subunit alpha	CCT1	1	4	9.2	59.229	545	33.354	6478600	6755000	5922400	6619000	7087400	8506000	9016500	8624900	9325900	8445800
P28996	1			1	1	1.2	94.114	845	6.7067	0	0	0	0	0	0	0	0	0	2869600
P29038	1			1	1	4.2	38.55	354	6.3084	0	0	0	0	0	0	0	0	0	1554400
P29102;P93832	1;1	3-isopropylmalate dehydrogenase 2, chloroplastic	IMDH2	2	1	3	43.35	406	6.892	0	0	0	0	0	0	0	0	0	849580
P29108;O22832;Q43593;Q41319;P32062;P32061	1;1;1;1;1;1;1;1;1;1;1;1;1	Acyl-[acyl-carrier-protein] desaturase 7, chloroplastic	FAB2	13	1	4.3	45.347	398	8.1702	0	0	0	0	0	0	0	0	0	598760
P29197	1	Chaperonin CPN60, mitochondrial	CPN60	1	1	4	61.28	577	153.56	0	0	0	0	0	0	0	0	0	477260
P29307	3;0;0;0;0;0;0;0;0;0			10	3	13.5	29.255	260	236.82	1254500	1458100	1046700	1266300	2505300	1604000	1871900	1796900	1575300	1503900

															0	0	0	0	0
P29409	2			1	2	8.1	45.572	433	62.815	37666000	38435000	33415000	37140000	34166000	43969000	33583000	48428000	41015000	40396000
P29545;O81918	3;2			2	3	14.3	23.799	224	54.973	8096600	10477000	12135000	12424000	10245000	12253000	14870000	17574000	17356000	19073000
P29610	3;1;1;1			4	3	18.5	28.592	260	25.366	14561000	12566000	12561000	13513000	14147000	23610000	25948000	21547000	22527000	23059000
P29696	1			1	1	2.8	39.765	357	5.9629	0	0	0	0	0	0	0	0	0	1110100
P29828;Q9SRG3	2;1	Protein disulfide isomerase-like 1-2	PDIL1-2	2	2	4.3	57.087	512	45.516	14602000	14698000	15434000	14400000	14893000	12698000	12468000	12614000	12151000	12872000
P30167	1			1	1	7.4	41.785	377	171.34	0	0	0	0	0	28816000	28277000	32046000	21521000	22484000
P30184;Q944P7;Q6K669;Q8RX72	2;2;1;1	Leucine aminopeptidase 1;Leucine aminopeptidase 3, chloroplastic;Leucine aminopeptidase 2, chloroplastic	PM25	4	2	6	54.509	520	31.313	7612200	7612400	7185900	8070400	8224100	3151000	3299100	3387200	3131000	3325300
P30362	1			1	1	3.6	32.462	306	7.5573	0	0	0	0	0	0	0	0	346710	0
P30707;Q9SZX9;P49209	2;2;2	60S ribosomal protein L9-2;60S ribosomal protein L9-1	RPL9D;RPL9B	3	2	5.7	21.752	193	12.655	18684000	20400000	15085000	29623000	28684000	6007200	6217400	5369200	6559400	7780500
P31167	1;1	ADP,ATP carrier protein 1, mitochondrial	AAC1	2	1	2.6	41.475	381	6.739	0	0	0	0	0	0	0	0	0	4350200
P31426;P45727;O04058;Q43210;Q42609;P35511;O49836;Q42858;O23865;Q9M568;O49835;P45733;P35513;Q0DZE0;O23924;A2X7F7;P4	2;2;2;2;2;2;2;2;2;2;2;2;2;2;2;2;2;2;2;2;2;2;2;2;2;2;2;2;2;1;1;1;1;1;1;1;1;1;1;1;1;1;1;1;1	Phenylalanine ammonia-lyase 3;Phenylalanine ammonia-lyase 4;Phenylalanine ammonia-lyase 2;Phenylalanine ammonia-lyase 1	PAL3; PAL4; PAL2; PAL1	45	2	4.9	64.143	590	27.291	3795800	3623800	3892200	3874800	3481300	2285700	2206700	2173300	2413000	0

5730;P25872;P45728;P24481;Q9SMK9;P45729;P31425;P26600;Q42667;P45725;Q9SS45;P45724;P35510;P45735;P07218;Q40910;P45731;P14717;Q8VXG7;P14166;Q43052;P19142;P27991;P45732;O64963;Q01861;Q04593;P45734;P27990;P31593;Q40147	1;1				2	1	2.7	50.877	478	6.7503	0	0	0	0	0	0	0	0	0	871510
P34788;P49202	2;1		40S ribosomal protein S18	RPS18A	2	2	13.2	17.545	152	12.465	13109000	12159000	0	12552000	0	0	0	0	0	0
P34789		1	40S ribosomal protein S28-2	RPS28C	1	1	18.8	7.3404	64	20.041	41891000	52448000	45891000	32169000	34755000	25769000	0	23079000	21826000	25315000
P34790		1	Peptidyl-prolyl cis-trans isomerase CYP18-3	CYP18-3	1	1	15.7	18.373	172	81.584	2585800	2259500	1938400	1921300	1959500	3032000	2764700	3868700	3183900	2688900
P35016;Q9STX5	5;2		Endoplasmin homolog	SHD	2	5	5	93.491	817	45.789	20132000	23018000	22937000	21015000	19017000	15587000	13818000	13283000	14715000	13532000
P35614;Q9LPV8	2;1		Eukaryotic peptide chain release factor subunit 1-3;Eukaryotic peptide chain	ERF1-3;ERF1-2	2	2	4.8	49.007	435	13.557	0	0	0	0	0	2272800	2339600	2283100	2306800	0

		release factor subunit 1-2																	
P35683; Q41741; Q40471; Q40465; P41379; Q6Z2Z4; P41378; Q40470; Q40467; P41382;P41381;Q40468;Q40469;P41377;P41376;Q9CAI7	5;4;4;4;4;4;4;4;4;4;3;3;3;2;2;2;2;0	Eukaryotic initiation factor 4A-2;Eukaryotic initiation factor 4A-1;Eukaryotic initiation factor 4A-3	TIF4A-2;TIF4A-1; TIF4A-3	18	5	21.3	47.087	414	88.529	13901000	18425000	15693000	17336000	19210000	60217000	63979000	53862000	45549000	48544000
P35684	1			1	1	4.1	44.463	389	5.8137	0	0	0	0	561270	0	0	0	0	0
P35685	1			1	1	4.3	29.304	258	6.8723	0	0	0	0	0	0	0	0	0	218430
P36181	1			1	1	2.1	80.135	699	180.11	680160	743010	575780	589540	664290	10328000	9673100	9087900	8752600	9933100
P36212;P36210	1;1	50S ribosomal protein L12-3, chloroplastic;50S ribosomal protein L12-1, chloroplastic	RPL12C;RPL12A	2	1	4.8	19.682	187	6.2351	0	0	0	0	0	0	0	0	4644300	0
P36397; O48920; O48649; Q06396; Q9LQC8; P0DH91; P51822;P51823;P51821;P49076;P51824;O23778	10;9;9;9;9;9;8;8;8;8;8;6	ADP-ribosylation factor 1;ADP-ribosylation factor 2-A;ADP-ribosylation factor 2-B	ARF1; ARF2-A;ARF2-B	12	10	74.6	20.608	181	312.91	1.38E+08	1.32E+08	1.26E+08	1.29E+08	1.3E+08	1.9E+08	1.82E+08	1.96E+08	1.93E+08	1.86E+08
P37115	1			1	1	2.4	57.887	505	7.2168	0	0	0	0	0	0	0	0	0	566000
P37221	1			1	1	1.9	69.952	626	10.662	2241900	2352200	1917800	2204200	1874500	3147700	2886300	3336900	3209200	2896700

P37225;Q8L7K9	1;1	NAD-dependent malic enzyme 2, mitochondrial	NAD-ME2	2	1	2	66.271	601	5.9428	0	0	0	0	0	0	0	0	0	1034500
P37228;P46488;P19446	1;1;1			3	1	3.1	37.394	353	6.8184	0	0	0	0	0	0	0	0	0	321530
P37399	3;0			2	3	6.6	59.135	547	323.31	1.44E+08	1.49E+08	1.63E+08	1.3E+08	1.65E+08	1.28E+08	1.26E+08	1.35E+08	1.22E+08	1.15E+08
P37829;Q7XJ81;Q42896;Q9M1B9;Q9SID0;Q9LNE4;Q6XZ79;Q0JGZ6;A2WXV8;Q9SX54;Q9LNE3	3;3;3;3;2;2;1;1;1;2;1	Probable fructokinase-4;Probable fructokinase-1;Probable fructokinase-3;Putative fructokinase-8;Probable fructokinase-2		11	3	11.6	33.764	319	39.218	24418000	24465000	21627000	22622000	22613000	7479000	5304800	5389800	5622300	7246100
P37900;Q01899;Q9LDZ0;Q8GUM2	2;1;1;2	Heat shock 70 kDa protein 10, mitochondrial;Heat shock 70 kDa protein 9, mitochondrial	HSP70-10;HSP70-9	4	2	4.6	72.3	675	123.27	7731300	7710900	8287300	7222800	6965300	6082800	6425400	6441300	6389200	5873000
P38661;Q942L2;O22263	1;1;1	Protein disulfide-isomerase like 2-1	PDIL2-1	3	1	4.1	40.492	364	5.7982	0	0	0	0	0	0	0	0	0	538890
P40392	1			1	1	9.4	22.475	202	101.15	1082700	1418900	1097700	1169700	1014100	3246000	3830500	3326500	3289900	2989500
P40393;Q9S810;Q1PEX3;Q40521;Q40195;Q40194;Q01111;Q9LK99;Q9FJH0	1;1;1;1;0;0;0;0;0	Ras-related protein RABA1i;Ras-related protein RABA1h;Ras-related protein RABA1g;Ras-related protein RABA1f	RABA1I;RABA1H;RABA1G;RABA1F	9	1	4.6	23.923	217	6.8329	0	0	0	0	0	0	0	0	3946900	0
P40782	1			1	1	2.1	51.564	473	5.7868	0	0	0	0	0	0	0	0	0	2545600
P40934;P49099;Q4	1;1;1	Cytochrome b5 isoform E	CYTB5-E	3	1	6.7	15.062	134	6.1289	0	0	0	0	0	0	0	1392000	0	0

2342																			
P41090	1			1	1	3	40.874	364	7.7051	0	0	0	0	0	0	0	0	0	1297300
P41095;O24573;P50345;O04204;Q42112;P57691	1;1;1;1;1;1	60S acidic ribosomal protein P0-1;60S acidic ribosomal protein P0-2;60S acidic ribosomal protein P0-3	RPP0A;RPP0B;RPP0C	6	1	4.1	34.377	319	7.8415	0	0	0	0	0	0	0	0	0	18515000
P41099	1			1	1	8.8	11.502	114	9.608	0	0	0	0	0	0	0	0	0	57783000
P42054	1			1	1	4.3	29.596	276	6.0425	0	0	0	0	0	0	0	0	0	2970200
P42066	2			1	2	3.7	74.396	670	15.041	0	0	0	0	0	0	0	0	0	705110
P42210;Q9XEC4	1;1	Aspartic proteinase A3	APA3	2	1	2	54.226	508	6.5912	0	0	0	0	0	0	0	0	0	216550
P42643	1	14-3-3-like protein GF14 chi	GRF1	1	1	4.1	29.931	267	6.1386	0	0	0	0	0	0	0	0	0	123140
P42763	1	Dehydrin ERD14	ERD14	1	1	6.5	20.786	185	5.956	0	0	0	0	0	0	0	0	0	241920
P42815	1	Ribonuclease 3	RNS3	1	1	9	25.626	222	13.672	0	0	0	0	0	0	0	0	0	274730
P42856	1			1	1	11.7	14.3	128	12.622	0	0	0	0	0	0	0	0	0	1288800
P42895;Q42971	1;0;0			3	1	5.6	48.162	446	49.727	5456900	9832400	7014500	5931100	4630200	42840000	57578000	38168000	45107000	19317000
P42896;Q9LEJ0;P26300	1;1;1			3	1	4	47.912	445	18.958	8067700	6907300	6897700	6522000	5619900	5901500	5972500	5673400	5433100	4935400
P43156;Q9STL4	1;1	KDEL-tailed cysteine endopeptidase CEP2	CEP2	2	1	3.3	39.914	360	6.7514	0	0	0	0	0	0	0	0	0	1144700
P43333	3	U2 small nuclear ribonucleoprotein A		1	3	14.9	28.041	249	18.978	6961200	7322500	5925700	6857600	6383600	6687800	6302200	5183900	5599600	5072600
P46248	3;1	Aspartate aminotransferase, chloroplastic	ASP5	2	3	6.8	49.831	453	29.401	2695000	2422700	2333600	2359400	2552300	2435400	2430700	2618300	2904900	2705200
P46276;P46267;Q42649;Q43139;Q9MA79	2;1;1;1;1	Fructose-1,6-bisphosphatase, cytosolic		5	2	7.9	37.31	340	43.25	2040100	2020800	2058800	1816100	2167400	2628700	2087700	1881400	2368600	2339500
P46287;P42795;P4	1;1;1	60S ribosomal protein L11-1;60S	RPL11A;RPL	3	1	7.7	20.683	181	16.942	0	0	0	0	0	0	0	0	890320	0

2794		ribosomal protein L11-2	11B																
P46291;O22860	1;1	60S ribosomal protein L38	RPL38A	2	1	17.4	8.0836	69	7.6745	0	0	0	0	0	0	0	0	0	830510
P46466	1			1	1	4.5	49.586	448	168.66	0	0	0	0	0	0	0	0	0	530790
P46487	1			1	1	3.2	36.462	347	8.5524	0	0	0	0	0	0	0	0	0	2152600
P46513;Q94CE4	1;1	Beta carbonic anhydrase 4	BCA4	2	1	5.3	20.585	190	6.6964	0	0	0	0	0	0	0	0	0	3742200
P46637	1	Arginase 1, mitochondrial	ARGAH1	1	1	4.1	37.344	342	6.9704	0	0	0	0	0	0	0	0	0	1244800
P46644	1	Aspartate aminotransferase 3, chloroplastic	ASP3	1	1	3.8	48.954	449	28.626	0	0	0	0	0	0	0	0	0	1667200
P47998;O23733	1;1	Cysteine synthase 1	OASA1	2	1	5.9	33.805	322	15.51	0	0	0	0	0	0	0	0	0	2559000
P47999;P31300	1;0;0;0	Cysteine synthase, chloroplastic/chromoplastic	OASB	4	1	2.8	41.656	392	24.833	0	0	0	0	0	0	0	0	0	3418700
P48352;P48351	1;1			2	1	2.6	57.136	492	11.596	0	0	0	0	0	0	0	0	0	3958600
P48390;P48392	1;1			2	1	2.5	43.494	398	5.975	0	0	0	0	0	0	1149900	0	0	0
P48496	1			1	1	4.7	34.409	322	7.768	0	0	0	0	0	0	0	0	0	3042200
P48502;Q9SUU5;F4JWS8	1;1;1	Cytochrome b-c1 complex subunit 7-1;Cytochrome b-c1 complex subunit 7-2	QCR7-1;QCR7-2	3	1	8.9	14.471	123	10.892	28618000	29635000	33442000	26401000	38804000	14144000	12224000	13767000	14141000	0
P48534	1			1	1	3.6	27.192	250	5.791	0	0	0	0	0	0	0	0	0	16839000
P48642	1			1	1	3	53.506	496	7.563	0	0	0	0	0	0	0	0	0	4080600
P48702	1			1	1	3.4	52.526	475	7.2419	0	0	0	0	868010	0	0	0	0	0
P48705;P43223;Q36610;P43226;P43228;P43222;Q31674;Q33015;P48712;P48710;P48707;P48	2;2;2;2;2;2;2;2;2;2;2;2;2;2;2;2;2;2;2;2;2;2;2;2;2;2;2;2;2;2;2;2;2;2;0;			407	2	3.9	45.285	410	22.263	0	0	0	7175000	0	0	0	0	0	0

701;Q32890;Q32770;Q32668;Q32663;P48704;P33536;Q32026;Q9MVE5;Q8SN66;Q31795;Q1ACK0;P48696;P48695;P36476;P26964;P24680;P43224;Q32016;Q32RQ1;Q19V64	0;0																		
P48977;P25141;P17648;P12886;P28032;P14675;P14674;P14673	1;1;0;0;1;1;1;1			8	1	2.6	41.408	380	19.098	0	0	0	0	0	0	0	0	1000200<br>0	0
P49027	1			1	1	3.3	36.231	334	7.7631	0	0	0	0	0	0	0	0	0	3455800<br>0
P49040	1	Sucrose synthase 1	SUS1	1	1	3.2	92.996	808	5.9258	0	0	0	0	0	0	0	0	0	616000
P49100;O48845	1;1	Cytochrome b5 isoform B	CYTB5-B	2	1	13.1	15.282	137	7.7176	0	0	0	0	2966600	0	0	0	0	0
P49103;P49104;Q39570;P36863;P92963;Q38922	5;5;5;5;5;4;2	Ras-related protein RABB1c;Ras-related protein RABB1b	RABB1C;RABB1B	7	5	32.1	23.001	209	44.586	1142300<br>0	1109900<br>0	1149300<br>0	1162400<br>0	1158400<br>0	1775600<br>0	1623000<br>0	1754300<br>0	1747900<br>0	1613400<br>0
P49199;Q08069;Q93VG5	1;1;0	40S ribosomal protein S8-1	RPS8A	3	1	6.8	24.829	220	21.502	0	0	0	0	0	0	0	0	0	4725500
P49215;Q9SQZ1;	2;2;2;2;2	40S ribosomal protein S17-3;40S	RPS17C;RPS	5	2	8.3	16.222	144	14.245	1917400<br>0	4885400	6015500	1994000<br>0	1746000<br>0	811460	723900	809200	814480	813140

Q9SJ36; Q9LZ17; P49205		ribosomal protein S17-2;40S ribosomal protein S17-4;40S ribosomal protein S17-1	17B;RPS17D;RPS17A																
P49249;P40691;Q7XT99;A2XRZ0;Q7XQ45;A2XRZ6;F4HPY8;Q9ASZ9;Q93ZN2;Q84M96;O22707	2;2;2;2;2;2;2;2;2;2;1	Probable aldo-keto reductase 6;Probable aldo-keto reductase 5;Probable aldo-keto reductase 4;Probable aldo-keto reductase 2;Probable aldo-keto reductase 3	AGD2	11	2	8.8	33.828	306	18.821	2114700	2905800	0	2630400	2787700	6763600	4542500	4367500	6683600	6771000
P49298	1			1	1	3.4	52.182	471	21.56	0	0	0	0	0	0	0	0	0	392540
P49317;P25890;P25819;P18122;Q01297	1;1;0;1;0;1;1;1;1;1;1;0	Catalase-2	CAT2	12	1	3	57.016	492	35.938	0	0	0	0	0	0	0	0	0	331180
P49351; Q7XYT0;P49352; O24242; O24241; P49350; Q7XYS9; Q43315; Q09152	2;1;1;1;1;1;1;1;1	Farnesyl pyrophosphate synthase 2;Farnesyl pyrophosphate synthase 1, mitochondrial	FPS2; FPS1	9	2	6.4	39.269	342	13.17	5421000	3640400	7386600	14027000	13036000	0	0	0	0	0
P49358;P49357;P50433;P34899;Q9SZJ5;Q94C74	1;1;1;1;1;1	Serine hydroxymethyltransferase 1, mitochondrial;Serine hydroxymethyltransferase 2, mitochondrial	SHM1;SHM2	6	1	3.1	56.933	517	7.8337	0	0	0	0	0	0	0	0	0	795720
P49362; O49852;	1;1;1;1;1;1;1;1	Glycine dehydrogenase	GLDP1;GLD	8	1	0.9	112.78	1034	6.2775	0	0	0	0	0	0	0	0	0	2384600

O49850;O49954;P49361;P26969;Q94B78;O80988		(decarboxylating) 1, mitochondrial;Glycine dehydrogenase (decarboxylating) 2, mitochondrial	P2																
P49390;P04165;Q9ZZT8;P05718;P29757;P09843;P0C524;P0C523;P07747;P42792	1;1;1;1;1;1;1;1;1;1	Cytochrome b	MT-CYB	10	1	10.1	10.215	89	6.3715	0	0	0	0	0	0	0	0	0	1999900
P49608;Q42560	1;1;1	Aconitate hydratase 1	ACO1	3	1	2.8	98.004	898	33.835	730260	976500	788970	0	678540	5867500	6081000	2706700	6050800	4863400
P49612;Q6F3F3;P24260	1;0;0			3	1	5.2	39.939	366	26.624	3292200	3215800	2900700	2599400	0	3403400	2971200	2962500	3296200	2716300
P49627	1			1	1	4	23.391	202	6.1006	12355000	11717000	13342000	13265000	12665000	0	0	0	0	0
P49690	1	60S ribosomal protein L23	RPL23A	1	1	14.3	15.027	140	15.093	0	0	0	0	0	0	0	0	0	271350
P50888;Q9FUL4;O65743	1;1;0			3	1	7.4	18.399	162	15.25	0	0	0	0	1396800	0	0	0	0	0
P51430;P29345;Q9M3V8;O48549	2;1;1;1	40S ribosomal protein S6-2;40S ribosomal protein S6-1	RPS6B;RPS6A	4	2	9.6	28.162	249	17.818	21987000	19666000	24036000	23154000	18520000	0	9950400	10235000	6879300	0
P52428	1			1	1	5.6	29.63	270	19.976	8580600	9035500	8938200	13459000	8073400	6362700	6581500	6240000	0	6642500
P52596	1			1	1	4.9	44.545	411	6.7998	0	0	0	0	0	0	0	0	0	3909600
P52885	1			1	1	9.6	22.932	198	7.5252	0	0	0	0	0	0	0	0	0	4256600
P52903;P52902;Q8H1Y0	1;1;1	Pyruvate dehydrogenase E1 component subunit alpha-2, mitochondrial	IAR4	3	1	3.1	43.228	391	7.5413	0	0	0	0	0	0	0	0	0	1407600
P52904	1			1	1	4.5	38.793	359	38.021	0	0	0	0	0	0	0	0	0	4227500
P53504	1			1	1	4.2	41.862	377	12.344	0	0	0	0	0	0	0	0	1584200	0

P53799	1	Squalene synthase	SQS1	1	1	2.7	47.141	410	5.7895	0	0	0	0	0	0	0	0	0	178950
P54243;P54240;P54239;P54236;P54234;P54242;P29333;P54235;P34796	7;4;4;4;4;4;4;4;3;3;2;2;1;1;1;1;1;1;1			20	7	19.2	62.685	568	56.509	17002000	16643000	18891000	14134000	15305000	8599200	9926900	10238000	12917000	10650000
P54411;P40412;O04450	2;2;2	T-complex protein 1 subunit epsilon	CCT5	3	2	4.5	59.033	535	14.059	0	0	0	0	873940	0	0	0	0	0
P54609	1	Cell division control protein 48 homolog A	CDC48A	1	1	2.2	89.392	809	123.49	0	0	0	0	0	0	0	0	0	989220
P54766;P54765;Q7GD79;Q7F7I7;P41919;P41918;P38548;P38547;P38546;A2Y7R5;A2WSI7;Q8H156;P41917;P41916	1;1;1;1;1;1;1;1;1;1;1;1;1;1	GTP-binding nuclear protein Ran-3;GTP-binding nuclear protein Ran-2;GTP-binding nuclear protein Ran-1	RAN3;RAN2;RAN1	14	1	6.2	23.73	209	6.7691	0	0	0	0	0	0	0	0	0	1643600
P54776;O04019;O23894;P46465;Q9SEI2	4;3;2;2;2	26S protease regulatory subunit 6A homolog B;26S protease regulatory subunit 6A homolog A	RPT5B;RPT5A	5	4	14.4	47.505	423	47.833	3690200	3439700	3555200	3335700	3315700	8085700	8033400	8962600	11775000	7434300
P54778;P85200;Q9SEI4	3;3;1	26S protease regulatory subunit 6B homolog	RPT3	3	3	18.9	46.532	413	66.833	2432400	2515300	4224700	3390200	2688300	5688600	5034000	5294400	5621000	4366400
P55310;P55308	1;1			2	1	2.8	56.599	492	11.224	0	0	0	0	0	0	0	0	0	508900
P55737;P51818	2;2	Heat shock protein 90-2;Heat shock protein 90-3	HSP90-2;HSP90-3	2	2	2.3	80.063	699	39.486	10356000	11770000	0	12371000	8203600	8113800	7024300	8392000	9666800	8140700

P55852	1	Small ubiquitin-related modifier 1	SUMO1	1	1	15	10.976	100	41.777	0	0	0	0	0	0	0	0	0	5102900
P55939; O82549; Q9SSQ9	1;0;0	Phospholipase D alpha 2	PLDALPHA2	3	1	1.7	92.061	812	23.741	0	0	0	0	0	0	0	0	0	336090
P59230	1	60S ribosomal protein L10a-2	RPL10AB	1	1	6.5	24.425	216	7.3184	2730900	2986100	5994600	2761000	2645500	0	1026300	1085900	1127000	0
P60118;P60117;P60116;P60115;P60114	1;1;1;1;1			5	1	41.9	7.563	74	30.544	120610	162960	0	0	0	1069300	2593500	2239500	0	1525000
P62302;P46298;P59224;P59223	1;1;1;1	40S ribosomal protein S13-2;40S ribosomal protein S13-1	RPS13B;RPS13A	4	1	11.3	17.14	151	10.912	0	0	0	0	1647000	0	0	0	0	0
P62773;P62772;P00073;P00071;P00069;P00067;P00065;P00064;P00062;P00061;P00060;P00056;P00054;P00053;P00052;P00051;P15451;P00070;P00068;P00063;O22642;Q9T0G2;O23138	1;1;1;1;1;1;1;1;1;1;1;1;1;1;1;1;1;1;1;1;1;1;1	Cytochrome c-2;Cytochrome c-1	CYTC-2;CYTC-1	23	1	7.2	12.083	111	6.215	0	0	0	0	0	1040800	0	0	0	0
P68173;P68172;P93253;Q9SWF5;Q	2;2;1;2;2;2;1;1;1;1;1	Adenosylhomocysteinase 2;Adenosylhomocysteinase 1	SAHH2;SAHH1	11	2	7.8	53.103	485	179.81	77461000	70997000	70767000	65837000	60016000	33257000	35520000	33990000	30136000	29067000

9LK36;O23255;P50249;P50246;Q9SP37;P35007																			
P80269; O24143; Q9FX83; Q42599	2;2;2;2	NADH dehydrogenase [ubiquinone] iron-sulfur protein 8-B, mitochondrial;NADH dehydrogenase [ubiquinone] iron-sulfur protein 8-A, mitochondrial		4	2	11.4	26.378	229	22.223	6446700	5591200	5093800	6579000	5386900	10147000	9322800	0	10122000	10545000
P83372	2			1	2	7.9	52.282	469	84.896	0	0	0	0	0	3644700	0	4478300	3208700	3001500
P83970	1;1			2	1	1.6	104.68	951	6.3007	0	0	0	0	2864900	0	0	0	0	0
P84977;P49310;P49311;Q03250	2;1;1;1	Glycine-rich RNA-binding protein 7	RBG7	4	2	75	5.2176	48	15.617	42602000	42743000	39116000	53139000	53865000	34877000	37226000	41682000	0	40567000
P84988; Q9XF89	2;2	Chlorophyll a-b binding protein CP26, chloroplastic	LHCB5	2	2	49.1	6.17	53	12.956	7044000	6493400	5311000	5761800	5865800	0	0	0	0	0
P85333	1			1	1	100	1.2875	13	11.337	28077000	29719000	18717000	19173000	18450000	9590700	10858000	10554000	10297000	11307000
P85929;P85930;P47921;Q02254;O81372;P47922;Q96559;P47920; P47919; Q39839; P47923; Q852S5; Q01402; P39207; O64903	2;2;2;2;2;2;1;1;1;1;1;1;1	Nucleoside diphosphate kinase 1;Nucleoside diphosphate kinase II, chloroplastic	NDK1;NDPK2	15	2	100	1.8881	18	12.202	1.06E+08	1.04E+08	1.04E+08	1.46E+08	1.25E+08	54518000	57499000	68567000	58017000	57546000
P85942;P85941	1;1			2	1	35.7	3.2487	28	8.1856	0	0	0	0	0	0	0	0	0	1070700
P86008;P85346;Q9	1;1;1			3	1	100	1.0061	9	12.46	0	0	0	0	0	0	0	0	0	0

LEH3																			
P92974;P31251;P20973;P31252	1;0;0;0	Ubiquitin-activating enzyme E1 2	UBA2	4	1	1.5	119.62	1077	6.5208	0	0	0	0	0	0	0	0	0	1014900
P93000	1	Germin-like protein subfamily 2 member 3	GLP8	1	1	7.3	23.032	219	6.9652	0	0	0	0	0	0	0	0	0	397870
P93028	1	Ubiquitin-activating enzyme E1 1	UBA1	1	1	1	120.25	1080	13.24	0	0	0	0	0	0	0	0	551940	0
P93031; Q9SNY3	2;1	GDP-mannose 4,6 dehydratase 2;GDP-mannose 4,6 dehydratase 1	MUR1;GMD1	2	2	10.5	41.961	373	18.142	0	0	0	0	0	0	0	0	0	2016200
P93208	1			1	1	3.9	28.879	254	159.24	0	0	0	0	0	0	0	0	1390700	0
P93306; Q36450	6;3	NADH dehydrogenase [ubiquinone] iron-sulfur protein 2	NAD7	2	6	18.5	44.961	394	136.58	11461000	11873000	9852500	10674000	11357000	9979500	10252000	10060000	9829600	9642300
P93395; Q8LD27	2;1	Proteasome subunit beta type-6	PBA1	2	2	11.5	25.183	234	18.59	11534000	13587000	13377000	13052000	12375000	12798000	12285000	12733000	15570000	12821000
P93400; O04883; O04865; Q38882	1;0;0;0	Phospholipase D alpha 1	PLDALPHA1	4	1	2	91.855	808	27.225	0	0	0	0	0	0	0	0	0	993040
P93401; O05000	1;1	NADH-ubiquinone oxidoreductase chain 2	ND2	2	1	2.3	54.05	488	7.4762	0	0	0	0	0	0	0	170970	0	0
P93447	1			1	1	3.5	24.497	226	9.3247	0	0	0	0	0	0	0	0	950390	0
P93484;P93026	1;0	Vacuolar-sorting receptor 1	VSR1	2	1	2.1	68.895	623	18.346	0	0	0	0	0	0	0	0	0	2555300
P93541; Q9LEC8; Q43314; Q38946	1;0;0;0	Glutamate dehydrogenase 1;Glutamate dehydrogenase 2	GDH1;GDH2	4	1	2.9	44.813	412	18.191	0	0	0	0	0	0	0	2807100	0	0
P93768; Q9LNU4;Q06364; Q9LQR8	3;3;2;2	26S proteasome non-ATPase regulatory subunit 3 homolog A;26S proteasome non-ATPase	RPN3A;RPN3B	4	3	6.1	55.576	488	39.119	10338000	9992700	10685000	10948000	10374000	4711200	4270000	4507600	4678700	5124800

		regulatory subunit 3 homolog B																	
P93819; Q9SML8 ;Q9FSF0; P57106	2;1;1;1	Malate dehydrogenase, cytoplasmic 1;Malate dehydrogenase, cytoplasmic 2	MDH1 ;MDH 2	4	2	8.4	35.571	332	41.767	27276000	23121000	34762000	36533000	38624000	18957000	13414000	12350000	10512000	14993000
P93844	1			1	1	1.6	91.318	817	11.559	0	0	0	0	0	0	0	0	0	864470
Q00834	1			1	1	4	34.186	325	10.891	0	0	0	0	0	0	0	0	0	2396300
Q01525	2	14-3-3-like protein GF14 omega	GRF2	1	2	11.2	29.161	259	323.31	3254200	0	3156600	0	0	0	4245700	0	6106600	6066600
Q01781	1			1	1	1.9	53.181	485	6.119	0	0	0	0	0	0	0	0	0	6935100
Q02028	1			1	1	1.3	75.514	706	17.763	0	0	0	0	0	0	0	0	0	3363700
Q02200	1			1	1	5.3	34.646	322	8.6437	2194700	2036500	1547200	1941400	2072700	0	0	0	0	0
Q03194; Q7XPY2; P20431; Q42556; Q9LV11	2;0;0;1; 1	ATPase 3, plasma membrane-type;ATPase 9, plasma membrane-type;ATPase 11, plasma membrane-type	AHA3 ;AHA 9;AH A11	5	2	3.2	105.19	952	80.15	0	0	461940	491780	0	12693000	12696000	0	0	11406000
Q03460; Q9LV03; Q0JKD0; Q0DG35	2;2;1;1	Glutamate synthase 1 [NADH], chloroplastic	GLT1	4	2	1.5	240.37	2194	11.584	0	0	0	0	0	892740	857320	782390	0	816290
Q03682; Q03681	1;1			2	1	3.8	32.259	292	39.752	0	0	0	0	0	0	0	0	4592100	0
Q04960; P42825; Q94AW8 ;Q03363; P43644	2;2;2;1; 1	Chaperone protein dnaJ 2;Chaperone protein dnaJ 3	ATJ2; ATJ3	5	2	6.5	46.064	413	12.663	0	0	0	0	0	0	0	0	0	2182000
Q05046; Q05045	1;1			2	1	2.4	61.13	575	25.016	0	0	0	0	0	0	0	0	0	737700
Q05492	1			1	1	5.7	22.293	192	6.4503	0	0	0	0	0	0	0	0	0	104310
Q06009; Q10BT5; A2XN40; Q9XGH7 ;A3C4N5 ;P0C5D7; P48578	2;1;1;1; 1;1;1	Serine/threonine-protein phosphatase PP2A-3 catalytic subunit	PP2A3	7	2	11.5	35.711	313	44.626	0	0	0	0	0	1141800	1215600	0	0	0

Q06197	1;0;0			3	1	2.7	46.049	413	7.7223	8617300	8920900	9132300	7159800	7926400	3706600	3814600	3414400	4089700	4020500<br>8974800
Q06611	1	Aquaporin PIP1-2	PIP1-2	1	1	3.5	30.597	286	14.021	0	0	0	0	0	0	0	0	0	0
Q07353	1			1	1	6	41.466	369	7.4167	0	0	0	0	0	0	0	0	0	1424400
Q08276	1			1	1	3.8	73.076	682	6.4541	0	0	0	0	0	0	0	0	50610	0
Q08435;P22180;P23980;Q08436	1;0;0;0			4	1	1.9	105.15	957	36.383	0	0	0	0	0	0	0	0	0	440180
Q08479;O82514	1;1	Adenylate kinase 4	ADK1	2	1	5.8	26.406	241	11.047	5186000	5648900	5077200	5011700	6144600	0	0	6501700	6638700	7246800
Q08480;Q9FK35	1;0	Adenylate kinase 3	ADK2	2	1	6.2	26.675	243	34.204	0	0	0	0	0	0	0	0	0	5049700
Q09020	1			1	1	23.4	5.4511	47	7.749	0	0	0	0	0	0	0	0	0	4179800
Q0DJ99;Q0DJA0;P49661;Q93Y22	2;2;1;1	Coatomer subunit delta		4	2	4.2	57.502	523	16.603	0	5883600	5038300	4981200	7178700	5813400	5812500	5455000	5422400	4702600
Q0DK10;A2YDY2	1;1			2	1	7.7	20.836	182	6.855	0	0	0	0	0	0	0	0	0	851470
Q0E0P0	1			1	1	2.4	63.983	577	7.6992	3245000	2909200	2589700	3177200	2445600	1836200	1691800	1960700	1567100	1567900
Q0J6T3;Q9ZRF1	1;1			2	1	2.8	37.196	354	6.8767	0	0	0	0	0	0	0	0	0	2683700
Q0JHF8;A2WXB2;Q9SDL8	1;1;1			3	1	4.7	37.035	339	41.768	0	0	0	0	0	0	0	0	0	1217000
Q0JLS6	1			1	1	1.4	90.477	806	7.698	0	0	0	0	0	0	0	0	0	896610
Q0WM29	1	Methylmalonate-semialdehyde dehydrogenase [acylating], mitochondrial	ALDH6B2	1	1	2	65.927	607	7.257	483630	458640	591950	502440	459990	907600	798770	627430	854390	722350
Q0WNJ6;Q0WLB5	3;2	Clathrin heavy chain 1;Clathrin heavy chain 2	CHC1;CHC2	2	3	2.2	193.24	1705	25.138	1624400<br>0	1442700<br>0	1401700<br>0	1401000<br>0	1320500<br>0	1452500<br>0	1522500<br>0	1771600<br>0	1418700<br>0	1493800<br>0
Q10712;P31427	1;1			2	1	2.1	60.279	571	10.352	0	0	0	0	0	0	0	0	0	2906000
Q10CE4;B8AKX6	1;1			2	1	4.3	40.383	369	8.0984	0	0	0	0	0	0	0	0	0	1408200
Q19VA5;Q85X67;	1;0;0;0;0;0;0;0;	ATP synthase subunit alpha,	atpA	104	1	2.4	54.803	506	7.5973	0	0	0	0	0	0	0	0	0	773150

P41602;	0;0;0;0;	chloroplastic
Q9TL16;	0;0;0;0;	
P08215;	0;0;0;0;	
Q0P3K5;	0;0;0;0;	
Q06SI2;	0;0;0;0;	
Q06RE6;	0;0;0;0;	
P12112;	0;0;0;0;	
A4QLR8;	0;0;0;0;	
A1E9I8;	0;0;0;0;	
Q9MUT2	0;0;0;0;	
;Q32RL1	0;0;0;0;	
;B1A920;	0;0;0;0;	
A1EA05;	0;0;0;0;	
P56294;	0;0;0;0;	
O99015;	0;0;0;0;	
A6YG64;	0;0;0;0;	
A4QJR8;	0;0;0;0;	
Q8S8Y3;	0;0;0;0;	
Q85FN4;	0;0;0;0;	
Q85AU2;	0;0;0;0;	
Q7YJY4;	0;0;0;0;	
Q70XV0;	0;0;0;0;	
Q6YXK3	0;0;0;0;	
;Q6L3A1	0;0;0;0	
;Q6EW6		
3;Q6EN		
W6;Q6E		
NH7;Q68		
S21;Q5S		
CX6;Q58		
9B3;Q49		
L13;Q3V		
549;Q3C		
1H4;Q3B		
AQ7;Q33		
C53;Q2Q		
DA3;Q2		
MIK2;Q2		
MIB5;Q2		
L8Z1;Q2		
7S65;Q1		
KVU0;Q		

14FH2;Q
0ZJ35;Q0
G9X7;Q0
G9N4;Q0
9X32;Q0
9MJ3;Q0
9G61;Q0
9FX6;Q0
6H12;Q0
6GS5;Q0
6FX6;P0
C2Z6;P0
C2Z5;P0
C2Z4;P0
6450;P06
283;B2Y
1W2;B1
NWD5;B
0YPM5;
A9LYH0
;A9L981;
A8Y9G7;
A8W3H5
;A8W3A
9;A8SE5
9;A7Y3A
4;A7M8
Y9;A6M
MS9;A6
MMJ2;A
6MMA7;
A6MM21
;A6H5F1
;A4QLH
9;A4QL9
1;A4QL0
4;A4QK
R6;A4Q
KH7;A4
QK90;A4
QK03;A4
QJI4;A4

QJA0;A4GYP3;A1XGM3;A1XFU0;A1E9S1;A0ZZ20;A0A320;Q8WI30;Q332Y4;Q1KXW5;Q1ACM8;P26526;A6BM08;A2T317;Q9BBS3;Q2PMS8;A4GGB2;A7M951;Q8MA05;Q32RS8;P56757																			
Q1H595	1	Sm-like protein LSM2	LSM2	1	1	14	10.694	93	6.0566	0	0	0	0	0	0	0	0	0	1080700
Q1PER6	3;1;1;1;1	L-ascorbate peroxidase 2, cytosolic	APX2	5	3	13.9	28.006	251	24.729	33941000	39874000	56074000	62511000	47624000	13294000	14412000	13617000	6019600	9597700
Q20EV9	1			1	1	2.4	54.012	498	63.772	16669000	19154000	17165000	18567000	16656000	20803000	21316000	20493000	22957000	22135000
Q285L8;A7Q5X9;Q9XEG7;P49198;P33444;A5B4K1;Q9MBB3	2;1;1;1;1;1;1			7	2	11.5	29.727	261	63.069	5999500	6577600	6295700	5362800	5628000	5423300	4689800	5617800	4225900	4376900
Q2KNL5;P31657;P42495	1;1;1			3	1	2.8	38.768	357	6.7663	0	0	0	0	0	0	0	0	0	6804900
Q2PCF1	2;0;0;0;0;0;0;0;0;0;0;0;0;0;0;0;0;0;0;0;0;0;0;0;0			25	2	1.8	166.35	1461	13.869	0	0	0	0	7781500	0	0	0	0	0
Q2PF16;Q45QI7;	1;1;1;1;1			5	1	5.9	24.019	222	36.233	0	0	0	0	0	0	0	0	0	2803100

Q9M5B3																			
;P11650;																			
Q33DL3																			
Q2QD63;																			
Q9MTJ7;																			
Q9BBQ8																			
;Q7YJV1																			
;Q7FNS4																			
;Q70XY1																			
;Q6L376;																			
Q6EW26																			
;Q6ENT8																			
;Q68RY1																			
;Q4FFN0																			
;Q4FFM9																			
;Q49KX2																			
;Q3V508;																			
Q3C1K3;																			
Q3BAL2;	3;3;3;3;																		
Q33C06;	3;3;3;3;																		
Q332V1;	3;3;3;3;																		
Q2VEF4;	3;3;3;3;																		
Q2PMQ9	3;3;3;3;																		
;Q2MIG2	3;3;3;3;																		
;Q2MI75;	3;3;3;3;																		
Q2L932;	3;3;3;3;																		
Q1KXT3	3;3;3;3;																		
;Q14FD1	3;3;3;3;																		
;Q0ZIZ3;	3;3;3;3;																		
Q0G9T6;	3;3;3;3;																		
Q0G9J3;	3;3;3;3;																		
Q09WZ1	3;3;3;3;																		
;Q09MF1	3;3;3;3;																		
;Q09G20;	3;3;3;3;																		
Q09FT5;	3;3;3;3;																		
Q06RA6;	3;3;3;3;																		
Q06GX1;	3;3;2;2;																		
Q06GN4;	2;2;2;2;																		
Q06FP2;	2;1;1;1;																		
P19819;P	1;1;1;1;	Photosystem II																	
06411;P0	1;1;1;1;	CP47 reaction																	
5641;P04	1	center protein	psbB	93	3	5.1	55.903	506	67.441	5868900	6421900	5494000	5785300	6834500	0	0	0	0	0

160;B2X
WP9;B2
LML9;B
1NWH6;
B1A961;
B0Z5F4;
B0Z570;
B0Z4Y6;
A9QC94;
A9LYC6;
A9L9C2;
A8W3E7
;A8SEC9
;A7Y3H4
;A7M989
;A6MMX
0;A6MM
N3;A6M
ME8;A6
MM62;A
4QLM0;
A4QLD2
;A4QKV
7;A4QK
L8;A4Q
KD1;A4
QK44;A4
QJV7;A4
QJE1;A4
GYT7;A
4GGD1;
A1E9V0;
A0ZZ61;
A0A361;
O03061;
B5LMP9;
P56777;
Q85X12;
P41624;B
2Y1Y5;B
1VKC1;
A6H5K7;

A6BM29;A4QJM5 Q2QKB4;Q2QZL4;Q9ZR40;Q2R0Q1;Q8L716	1;1;1;1;1	Splicing factor U2af large subunit B	U2AF65B	5	1	1.8	60.586	543	6.7717	0	0	0	0	0	0	0	0	0	1097200
Q2QLY5;Q2QLY4;Q0WNZ5	1;0;0;0	5-methyltetrahydropteroyltriglutamate--homocysteine methyltransferase 3, chloroplastic	MS3	4	1	1.4	84.584	766	7.5597	0	0	0	0	0	0	0	0	0	1540200
Q2QM55;Q10G39;Q9C6Z3;O64688	1;1;1;1	Pyruvate dehydrogenase E1 component subunit beta-2, chloroplastic;Pyruvate dehydrogenase E1 component subunit beta-3, chloroplastic	PDH-E1 BETA;E1-BETA-2	4	1	2.6	42.12	391	6.8757	0	0	0	0	0	0	0	0	0	1860900
Q2QMN7;Q8VXY9	2;1	Ureidoglycolate hydrolase	UAH	2	2	5.4	51.715	484	13.417	0	0	0	0	0	0	0	2614000	0	0
Q2QV94	2			1	2	2.6	88.228	806	13.107	1504700	1534800	1432400	1748100	1591000	1644200	1595300	1715700	0	0
Q2RAK2;B8BJ39;Q2QXR8;B8BM17	5;5;4;4			4	5	14.4	57.319	527	65.131	6485300	4993500	6075600	6055100	4819100	12229000	11976000	10981000	12214000	13046000
Q2RBN7;Q2QYW2	4;4			2	4	2.4	193.34	1708	64.419	13588000	16265000	0	16875000	16804000	10322000	9638300	12808000	10327000	11683000
Q336X9;Q5J4W4;Q9LMM5;Q39024	1;1;1;1	Mitogen-activated protein kinase 11;Mitogen-activated protein kinase 4	MPK11;MPK4	4	1	4.8	42.772	376	10.869	0	0	0	0	0	0	0	0	0	1085300
Q35322;P80261;Q37787;Q34011;Q33994;P34944;	4;4;4;4;4;3;3;1	NADH dehydrogenase [ubiquinone] iron-sulfur protein 3	NAD9	8	4	18.9	22.991	190	25.834	10877000	12107000	13278000	11504000	10065000	3676800	4627000	5776000	6136100	5384700

Q95748																			
Q38707	1			1	1	3	39.69	365	7.6562	0	0	0	0	0	0	0	0	0	1692600
Q38858	1	Calreticulin-2	CRT2	1	1	3.8	48.156	424	6.6206	0	0	0	0	0	0	0	0	0	1650000
Q38936;Q41649;Q38935	2;1;1	Peptidyl-prolyl cis-trans isomerase FKBP15-2;Peptidyl-prolyl cis-trans isomerase FKBP15-1	FKBP15-2;FKBP15-1	3	2	16.6	17.658	163	12.284	6396600	5468000	5875000	5540300	5302900	0	0	0	0	0
Q38950;Q38845	1;0	Serine/threonine-protein phosphatase 2A 65 kDa regulatory subunit A beta isoform;Serine/threonine-protein phosphatase 2A 65 kDa regulatory subunit A alpha isoform	PP2AA2;PP2AA1	2	1	1.7	65.597	587	12.545	0	0	0	0	0	0	0	0	0	5821000
Q38951	1	Serine/threonine-protein phosphatase 2A 65 kDa regulatory subunit A gamma isoform	PP2AA3	1	1	3.7	65.516	587	15.719	0	0	0	0	0	0	0	0	0	3581000
Q39043	1	Mediator of RNA polymerase II transcription subunit 37f	MED37F	1	1	1.6	73.56	668	13.444	0	0	0	0	0	0	0	0	0	1460400
Q39097	2	Eukaryotic peptide chain release factor subunit 1-1	ERF1-1	1	2	5.7	48.722	436	12.442	0	3224700	0	0	0	0	0	1866400	1971900	0
Q39161	2	Ferredoxin--nitrite reductase, chloroplastic	NIR1	1	2	4.4	65.504	586	21.078	0	0	0	0	0	5033800	4565800	4156200	5078100	4151300
Q39230;O81983	3;2	Serine--tRNA ligase		2	3	5.8	51.628	451	27.209	0	0	0	0	0	3161500	2539500	2943500	2669200	0
Q39336	1			1	1	3.4	35.723	327	20.037	0	0	0	0	0	0	0	0	0	5729600
Q39434;Q40523;Q40193	1;1;1			3	1	4.7	23.787	214	7.3148	0	0	0	0	0	0	0	0	0	1692200
Q39471;	6;3			2	6	23.8	32.561	286	63.045	2526600	1856900	2774300	2582200	2282200	8558100	8582200	9171800	8147000	9509200

Q39664										0	0	0	0	0					
Q39613;										1669500	2213300	1812600	2119100	1619500	1449500	1427200	1776700	1515800	1747100
P21569	2;1			2	2	15.7	18.285	172	19.483	0	0	0	0	0	0	0	0	0	0
Q39817	1			1	1	2.9	62.08	546	21.688	0	0	0	0	0	0	0	0	0	1699000
Q39828; Q39821; P42697	2;2;1	Dynamin-related protein 1A	DRP1 A	3	2	3.4	68.328	610	13.527	1681900	0	0	0	0	0	1897200	1618000	0	1958900
Q40106; P37392;P 29516;Q9 ASR0;Q5 6YW9;Q 6VAF4;P 29501;P2 9517;P29 515;P295 00;P2463 6	1;1;1;0; 0;1;0;0; 0;0;0;0	Tubulin beta-8 chain;Tubulin beta-3 chain;Tubulin beta-2 chain;Tubulin beta-9 chain;Tubulin beta-7 chain;Tubulin beta-4 chain	TUBB 8;TUB B3;TU BB2;T UBB9; TUBB 7;TUB B4	12	1	3.6	50.34	448	239.67	0	0	0	0	0	0	0	0	1877100	0
Q40191	1			1	1	5.8	24.842	226	6.0275	0	0	0	0	0	0	0	0	0	4588200
Q40450; P68158; Q43467; P46280; Q43364; P17745	1;1;1;1; 1;1	Elongation factor Tu, chloroplastic	TUFA	6	1	3.3	51.956	478	22.591	0	0	0	0	0	0	0	0	0	1231200
Q41140	3;1			2	3	9.7	67.36	617	43.788	0	0	0	0	1196100	2453400	3011800	3242100	3352900	2646200
Q41142	1			1	1	2.8	91.991	808	7.7225	0	0	0	0	0	0	0	0	0	1644600
Q41266	1			1	1	4.2	38.128	333	7.6503	0	0	0	0	1128100	0	0	0	0	0
Q41328; O80719	1;1	Probable receptor-like protein kinase At2g47060		2	1	4.5	38.539	354	8.13	0	0	0	0	0	0	0	0	0	824810
Q41595	1			1	1	5.9	36.672	340	33.568	0	0	0	0	0	0	0	0	0	201350
Q41630; Q41629; O22342	1;1;1;1;1;1;1			7	1	3	35.79	331	19.865	0	0	0	0	0	0	0	0	7912400	0
Q41640	1			1	1	6.8	23.098	206	39.339	0	0	0	0	0	1707600	1921100	1977700	0	0
Q41769; Q6K2E8	1;1			2	1	2	69.025	638	6.5402	0	0	0	0	0	0	0	0	0	365070
Q41803; Q41011; Q40034; Q03033; P43643;P	1;1;1;1; 1;1;1;1; 1;1;1;1; 1;1;1	Elongation factor 1-alpha 2;Elongation factor 1-alpha 4;Elongation factor	A2;A4 ;A3;A 1	15	1	6.5	49.232	447	34.473	0	0	0	0	0	0	0	0	0	2607600

34823;P25698;O64937;O24534;P17786;O49169;Q8W4H7;Q8GTY0;Q0WL56;P0DH99		1-alpha 3;Elongation factor 1-alpha 1																	
Q41870;Q7XSQ9;Q9XF59;Q6EU94;Q9AQU5	2;2;2;2;2			5	2	5.9	30.885	287	21.333	5714400	6286800	5445000	5890000	4174300	12892000	10227000	14337000	16904000	0
Q42434	1			1	1	1	73.591	668	174.35	0	0	0	0	0	0	0	0	1124100	0
Q42521;Q9ZPS3;Q07346;Q9LSH2;Q42472;Q9ZPS4	6;5;4;3;3;3;2	Glutamate decarboxylase 1;Glutamate decarboxylase 4;Glutamate decarboxylase 5;Glutamate decarboxylase 2;Glutamate decarboxylase 3	GAD1;GAD4;GAD5;GAD2;GAD3	7	6	19.7	57.066	502	84.176	0	7195900	5189400	10758000	7908100	4663900	5694000	4603200	4152300	4063400
Q42586;Q9LPX2	1;1	Uridine 5-monophosphate synthase;Orotate phosphoribosyltransferase;Orotidine 5-phosphate decarboxylase;Pentatricopeptide repeat-containing protein At1g12775, mitochondrial	PYRE-F	2	1	2.1	51.85	476	6.7036	0	0	0	0	1701200	0	0	0	0	0
Q42662;P93263;O50008	4;1;1	5-methyltetrahydropteroyltriglutamate--homocysteine methyltransferase 1	MS1	3	4	9.7	84.589	764	267.64	51832000	60599000	48778000	52770000	50489000	38350000	43306000	45318000	38606000	40777000
Q42676	1			1	1	4	56.187	519	28.156	0	0	0	331710	0	8060000	7427000	9185500	7452400	3575500

Q42699	1;1			2	1	1.4	84.856	765	7.7854	0	0	0	0	0	0	0	0	0	4656700
Q42806	3			1	3	8.4	55.302	511	58.866	5152400	5955200	4660500	5374900	6109400	8561600	7887900	8148900	7802000	8080900
Q42919;Q9LK23	2;2;1;1	Glucose-6-phosphate 1-dehydrogenase, cytoplasmic isoform 1	ACG9	4	2	3.3	58.923	515	13.059	9678500	8253400	11062000	11021000	10086000	0	0	0	0	0
Q42942	1			1	1	2.6	49.76	461	6.9789	0	0	0	0	0	0	0	0	0	1347000
Q42961	2			1	2	5.4	50.176	481	106.27	5289200	4599800	4886100	4328200	4920700	6166400	6716000	6545200	6023700	5218200
Q42962;P50318;Q9LD57	1;0;0;0;0;0	Phosphoglycerate kinase 2, chloroplastic;Phosphoglycerate kinase 1, chloroplastic	PGK1	6	1	2.5	42.364	401	6.7274	0	0	0	0	0	0	0	0	8690000	0
Q43046;O82054;Q43047;Q41086;Q00763	1;0;1;1;0;0;0;0;0;0;0;0;0;0;0;0;0;1			18	1	3.3	39.791	365	24.077	0	0	0	0	0	0	0	0	0	3903000
Q43117	1			1	1	2.1	64.093	583	21.205	0	0	0	0	0	0	0	0	0	1505700
Q43128	1	ATPase 10, plasma membrane-type	AHA10	1	1	1.8	104.81	947	6.3577	0	0	0	0	1257000	0	0	0	0	0
Q43175	1			1	1	2.8	52.611	471	6.9414	1697000	2321600	2104200	2510100	2504700	0	0	1486900	0	0
Q43187;Q0DYB1;O48556;A2X8Q3;O23979;Q9LFF9	1;1;1;1;1;1	Soluble inorganic pyrophosphatase 4	PPA4	6	1	5.7	24.261	211	6.6205	0	0	0	0	0	0	0	0	0	2790200
Q43237;O81185;Q43095;Q42945;O65922;O65862;O04854	1;1;1;1;1;1;1;1			8	1	5	27.234	242	6.1297	0	0	0	0	0	0	0	12105000	0	0
Q43260	1			1	1	2.9	44.022	411	10.617	0	0	0	0	0	0	0	0	0	1229400
Q43266;P22177;Q00268;O82797;P24314;	2;1;1;1;1;1;1	Proliferating cellular nuclear antigen 1;Proliferating cell nuclear antigen 2	PCNA;PCNA2	7	2	11.4	29.342	263	13.021	0	0	0	0	3825900	0	11835000	11936000	11831000	11433000

Q9M7Q7;Q9ZW35																			
Q43317	1			1	1	4	34.342	325	141.69	0	0	0	0	0	0	0	0	0	3371400
Q43621	1			1	1	4	53.896	498	6.153	0	0	0	0	0	3509800	3126900	2865800	0	2653500
Q43644;Q9FGI6	2;1	NADH dehydrogenase [ubiquinone] iron-sulfur protein 1, mitochondrial	EMB1467	2	2	3.9	79.969	738	18.844	1699800	1590800	0	0	0	4573600	4166700	0	4044900	0
Q43715	1			1	1	1.2	88.268	809	6.8574	0	0	0	0	0	0	0	0	355570	0
Q43725	1	Cysteine synthase, mitochondrial	OASC	1	1	4.2	45.814	430	37.278	0	0	0	0	0	0	0	0	0	1361200
Q43727;O24357;Q43839	1;1;0	Glucose-6-phosphate 1-dehydrogenase 1, chloroplastic	APG1	3	1	2.6	65.427	576	51.409	0	0	0	0	0	0	0	0	0	554000
Q43729	1	Peroxidase 57	PER57	1	1	7	34.098	313	8.5338	0	0	0	0	0	0	0	0	0	460600
Q43772	1			1	1	2.5	51.644	473	6.5965	0	0	0	0	0	0	0	0	0	6397200
Q43793;Q9FY99;Q8L743	1;1;1	Glucose-6-phosphate 1-dehydrogenase 2, chloroplastic;Glucose-6-phosphate 1-dehydrogenase 3, chloroplastic		3	1	2	67.368	593	7.2742	0	0	0	0	0	0	0	0	0	1131700
Q43844;P42027;Q42577	1;1;1	NADH dehydrogenase [ubiquinone] iron-sulfur protein 7, mitochondrial		3	1	4.2	23.396	213	12.976	0	0	0	0	0	0	0	0	0	613880
Q43872	1	Peroxidase 64	PER64	1	1	5.7	34.705	317	6.5519	0	0	0	0	0	0	0	0	0	957540
Q43873	2	Peroxidase 73	PER73	1	2	6.7	35.927	329	37.368	11189000	11245000	10883000	12200000	11827000	6259700	6115700	6135400	6008800	6261100
Q4H1G3;P43281;Q9M7K8;Q96553;Q38JH8;P50301;P48498;P43282;A9PHC5;A7PQS0;Q94FA6;Q94FA4;Q5DNB1;A	1;1;0;0;0;0;0;0;0;0;0;0;0;0;0;0;0;0;0;0;0	S-adenosylmethionine synthase 1;S-adenosylmethionine synthase 2	SAM1;SAM2	21	1	5.3	42.96	393	176.61	0	0	0	0	0	2895100	2933900	2778900	2780600	2682900

4PU48;Q9FPL6;P23686;P17562																			
Q4W1I9;Q4W1I8	1;1			2	1	5.3	34.232	321	21.107	21462000	11080000	15042000	10252000	9567900	0	0	0	0	945460
Q56ZQ3;O80977	1;1	Vacuolar-sorting receptor 4;Vacuolar-sorting receptor 3	VSR4;VSR3	2	1	1.3	69.812	628	11.412	0	0	0	0	0	0	0	0	0	1301500
Q5E924	1	Glyceraldehyde-3-phosphate dehydrogenase GAPCP2, chloroplastic	GAPCP2	1	1	3.3	44.845	420	15.351	0	0	0	0	0	0	0	0	0	5209700
Q5I7K9	1			1	1	10.7	12.275	112	10.702	0	0	0	0	0	0	0	0	341860	0
Q5JK84;Q0JM17	1;1			2	1	2.8	48.566	432	19.54	0	0	2651200	0	1832400	0	0	0	0	0
Q5VQ78	1			1	1	1.9	102.89	907	12.214	0	0	0	0	0	0	0	0	0	295790
Q5XF03;Q9LMM0	1;1	Probable glycerol-3-phosphate acyltransferase 8;Glycerol-3-phosphate 2-O-acyltransferase 4	GPAT8;GPAT4	2	1	2.2	55.867	500	7.7414	0	0	0	0	807780	0	0	0	0	0
Q5Z627;Q6YW46;Q9ZRI7;Q9FUM1	2;2;1;1			4	2	6	47.39	416	21.547	1636300	1574100	2705300	0	3116000	0	0	0	0	0
Q65CJ7	1			1	1	5.4	34.127	313	6.9881	0	0	0	0	0	0	0	0	0	1585700
Q65XK0;O82043;Q05758	2;1;1	Ketol-acid reductoisomerase, chloroplastic		3	2	5.7	62.376	578	91.574	3069300	2626200	2263100	2873500	0	0	0	0	0	7818600
Q66GI2	1	Dirigent protein 22	DIR22	1	1	9.6	13.604	125	8.7858	0	0	0	0	0	0	0	0	0	2492100
Q683D5	1	U-box domain-containing protein 37	PUB37	1	1	3	46.024	400	6.4421	0	0	0	0	0	0	0	0	0	148880
Q69RJ0	1			1	1	0.7	175.04	1615	7.1	0	0	0	0	0	0	0	0	0	456840
Q69TH4;B9FFD3;Q84QK0;	1;1;1;1;1			5	1	2.7	42.068	376	6.9195	0	0	0	0	0	0	0	0	0	1610700

Q69TH6; B9FFD2																			
Q69TY4	1			1	1	4.7	23.688	232	7.4428	0	0	0	0	0	0	0	0	0	6172400
Q69U49	1			1	1	3	50.303	461	5.8934	0	0	0	0	0	0	0	0	0	540070
Q6AVG6	1			1	1	2.8	51.063	459	6	0	0	0	341030	0	0	0	0	0	0
Q6AVK1;A2XKU9;B4YYA9;A9NK39;Q8LBN7	1;1;1;1;1	Costars family protein At4g33640		5	1	14.9	9.5799	87	106.3	0	0	0	0	0	0	0	0	0	1779200
Q6ER94; P84729; Q96468; P80602; Q9C5R8	1;0;0;0;0	2-Cys peroxiredoxin BAS1-like, chloroplastic		5	1	6.1	28.097	261	28.066	7908200	7973800	9075500	7986700	8289500	8255900	6760200	6124300	0	7628200
Q6F2Y7; P42730; Q75GT3; F4JVJ1; Q9LF37	2;2;1;1;1	Chaperone protein ClpB1;Putative chaperone protein ClpB2, chloroplastic;Chaperone protein ClpB3, chloroplastic	CLPB1;CLPB2;CLPB3	5	2	3.1	100.9	912	11.766	0	0	0	0	559270	0	0	0	0	0
Q6F2Z1; Q93ZY3	1;1	Dolichyl-diphosphooligosaccharide--protein glycosyltransferase subunit STT3A	STT3A	2	1	1.5	87.013	787	5.9614	0	0	0	0	1753300	0	0	0	0	0
Q6H5J0; Q6Z0I4	1;1			2	1	4.9	39.02	371	10.829	0	0	0	0	0	0	0	0	0	492660
Q6IMT1	1	Protein SABRE	SAB	1	1	0.4	292.91	2603	6.6781	0	0	0	0	0	0	0	0	0	332820
Q6K5G8	1;0;0;0			4	1	8.3	36.567	337	54.746	0	0	0	0	0	7541700	1170800<br>0	8606200	8492400	8162400
Q6K9N6	2			1	2	5.9	45.091	422	35.952	2166700	2145300	2188300	2262800	2040400	2902600	4742800	0	0	5300000
Q6L5I5	1			1	1	4.3	29.602	280	6.0063	0	0	0	0	0	0	0	0	3099700	0
Q6QNU4;Q6E7D1;Q9M0V3;O49552	2;2;2;2	DNA damage-binding protein 1a;DNA damage-binding protein 1b	DDB1A;DDB1B	4	2	2.6	121.73	1090	12.144	0	1217000	0	1078800	0	0	0	740120	0	0
Q6R2K3	1	Protein STRUBBELIG-RE	SRF3	1	1	0.9	84.719	776	6.0115	0	0	0	0	0	0	0	0	0	1006500

		CEPTOR FAMILY 3																	
Q6UNT2;Q8L4L4;Q0JGY1;A2WXX3;Q8LBI1;P49227	1;1;1;1;1;1	60S ribosomal protein L5-1;60S ribosomal protein L5-2	ATL5;RPL5B	6	1	3	34.328	302	6.3912	0	0	0	0	0	0	0	0	0	19336000
Q6V4H0	1			1	1	2.8	38.937	360	6.9182	0	0	0	0	0	0	0	0	0	4574000
Q6YT73;B8B7C5	1;1			2	1	4.9	40.244	369	9.1542	0	0	0	0	0	0	0	0	0	2594100
Q6YZX6	1			1	1	1.2	98.082	898	25.56	0	0	0	0	0	0	0	0	0	928050
Q6Z1G7	1			1	1	10.4	39.944	374	69.636	0	0	0	0	0	2092400	2782400	2510800	2208000	1632300
Q6ZL94;P68209	2;2	Succinyl-CoA ligase [ADP-forming] subunit alpha-1, mitochondrial		2	2	5.7	34.246	331	12.741	0	0	0	0	0	0	0	0	0	2299300
Q6ZLK0;Q944K2	1;1	Dolichyl-diphosphooligosaccharide--protein glycosyltransferase 48 kDa subunit	OST48	2	1	2.5	48.389	439	7.723	0	0	0	0	0	0	0	0	0	283630
Q70EW5	1			1	1	1.5	91.782	808	27.77	0	0	0	0	0	0	0	0	0	6138300
Q70Z19;Q9ZUP3	1;1	Nucleosome assembly protein 1;2	NAP1;2	2	1	3.2	42.578	374	8.0738	0	0	0	0	0	0	0	0	0	1772500
Q71VM4;O04294;Q9FWY7	2;0;0;0;0;0;0	Importin subunit alpha-3;Importin subunit alpha-6	IMPA3;IMPA6	7	2	5.3	57.57	526	28.849	3991900	4013200	4374500	4584300	4094200	4261800	3958300	4034100	4387800	4363600
Q75IM9	1			1	1	3.9	44.531	409	6.4237	0	0	0	0	0	0	0	0	0	574070
Q75LJ3;A2XNR6	1;1			2	1	5.3	37.352	358	7.3252	0	0	0	0	0	0	0	0	0	596170
Q75LU8;Q3HRP5	2;1;0;0;0			5	2	12.4	25.803	225	19.422	2460000	2373200	2067600	2228800	2032800	3018800	0	0	0	2697800
Q75W16	3;0;0;0;0			5	3	5	59.333	539	24.154	2794600	2326100	2671200	2862600	2606300	0	0	0	0	0
Q76H85;Q71V09;Q6WZ83;Q6V9I2;Q6PMI5;Q6LAF3;Q41811;P62887;P	3;3;3;3;3;3;3;3;3;3;3;3;3;3;3;2;2;2	Histone H4		18	3	29.1	11.409	103	27.954	35872000	36181000	34461000	35110000	36101000	2.47E+08	2.76E+08	2.18E+08	2.6E+08	2.93E+08

62788;P62787;P62786;P62785;P35057;P0CG89;P59259;P85945;P50566;P08436																			
Q7DLR9	1	Proteasome subunit beta type-4	PBG1	1	1	7.3	27.651	246	32.629	2532600	2419400	2321900	2365200	2527900	0	3079400	3064900	3095600	3975700
										3733600	2893700	3824700	4769800	4046900	2413800	2579900	2132800	2514700	2569100
Q7M2G6	1	ATP synthase subunit alpha	ATP1	1	3	27.7	53.323	491	153.66	0	0	0	0	0	0	0	0	0	0
Q7PC84;Q7PC87	2;1	ABC transporter G family member 39;ABC transporter G family member 34	ABCG39;ABCG34	2	2	1.9	165.19	1454	19.365	0	0	0	0	0	2673300	2565500	2522900	2645400	2441100
Q7SIC9	1			1	1	5.2	72.993	675	107.24	0	0	0	0	0	0	0	0	0	2766300
Q7XI40;Q6K7H2;O24464;O04905;Q8VY84;Q6NMK6	1;1;1;1;1;1	UMP-CMP kinase 3;Probable UMP-CMP kinase 1;Probable UMP-CMP kinase 2	UMK3;UMK1;UMK2	6	1	3.8	23.234	210	6.2423	0	0	0	0	0	0	0	0	0	782990
Q7XQP4;Q6ZI44;Q7XKA8;Q7Y0B9;O64812;Q9C958;Q39193;Q940H6;Q39192;P43291	1;1;1;1;1;1;1;1;1;1	Serine/threonine-protein kinase SRK2J;Serine/threonine-protein kinase SRK2B;Serine/threonine-protein kinase SRK2I;Serine/threonine-protein kinase SRK2E;Serine/threonine-protein kinase SRK2D;Serine/threonine-protein kinase SRK2A	SRK2J;SRK2B;SRK2I;SRK2E;SRK2D;SRK2A	10	1	3.6	41.325	359	13.62	0	0	0	0	0	0	0	0	0	1143000
Q7XRB5	1			1	1	1.9	57.334	517	6.2184	0	0	0	0	0	0	0	0	0	6602300

Q7XTJ3	1			1	1	2.8	46.144	425	7.1752	0	0	0	0	0	0	0	0	0	2207000
Q84LB6; O82662	1;1	Succinyl-CoA ligase [ADP-forming] subunit beta, mitochondrial		2	1	4.3	44.839	417	9.0622	0	0	0	0	0	0	0	0	0	1708900
Q84MP7; Q8H7Y8; Q8S857; Q9T0H7; Q9C944; Q9SII0;O23628	1;1;1;1;1;1;1	Histone H2A.8;Probable histone H2A variant 3;Probable histone H2A variant 2;Histone H2A variant 1	H2AV	7	1	7.3	14.459	137	6.7951	0	0	0	0	0	0	0	929600	0	0
Q84P54; Q84P52	1;1			2	1	2.3	56.748	515	11.023	0	0	0	0	0	0	0	0	0	1102500
Q84TA3; Q9FY49	1;1	Leukotriene A-4 hydrolase homolog	LKHA4	2	1	1.6	67.791	611	6.6848	0	0	0	0	0	0	0	0	0	846040
Q84VX0	1	Probable galactinol--sucrose galactosyltransferase 1	RFS1	1	1	2.3	83.476	754	7.099	0	0	0	0	0	0	0	0	0	514200
Q84WU2;Q9FPT1	2;2	Ubiquitin carboxyl-terminal hydrolase 13;Ubiquitin carboxyl-terminal hydrolase 12	UBP13;UBP12	2	2	2.1	130.65	1115	12.672	0	0	0	0	0	0	0	0	1691900	0
Q851S8; C5WNV2	1;1			2	1	2.7	52.674	489	11.728	0	0	0	0	0	0	0	0	1254800	0
Q8GTQ9;Q6DQL1	1;1			2	1	8.7	34.684	332	10.168	0	0	0	0	0	1295900	1748200	1226700	0	1305500
Q8GXE2;Q8W108	1;1	1,2-dihydroxy-3-keto-5-methylthiopentene dioxygenase 2;1,2-dihydroxy-3-keto-5-methylthiopentene dioxygenase 3	ARD2;ARD3	2	1	5.2	22.588	192	6.459	0	0	0	0	0	0	0	0	0	1641400
Q8H103	2	Glucose-6-phosphate isomerase 1, chloroplastic	PGI1	1	2	4.1	67.048	613	37.911	2238300	2641300	2375600	2700200	2471500	2414700	2169500	1891900	1747500	2258200
Q8H104	1		At4g2	1	1	2.8	60.332	532	8.3857	2858000	0	0	0	0	0	0	1734700	1745600	0

Q8H173	2;0;0;0;0	40S ribosomal protein Sa-2	6870 RPSaB	5	2	4.3	30.653	280	57.144	27388000	25688000	28089000	26905000	22861000	22099000	21731000	22573000	21680000	0
Q8H852; Q6Z382; Q0WW26	1;1;1	Coatomer subunit gamma		3	1	1.1	98.605	884	6.7957	0	0	0	0	0	0	0	0	1532500	0
Q8L4A7; O23708	3;3	Proteasome subunit alpha type-2-B;Proteasome subunit alpha type-2-A	PAB2; PAB1	2	3	17.9	25.733	235	19.412	11805000	12083000	12802000	11921000	12177000	14781000	14907000	13848000	13987000	12863000
Q8L6Y1	1	Ubiquitin carboxyl-terminal hydrolase 14	UBP14	1	1	1.5	88.373	797	6.8817	0	0	0	0	0	0	0	0	0	711340
Q8L719	1	THO complex subunit 4B	ALY2	1	1	5.5	30.78	292	7.2126	0	0	0	0	0	0	0	0	0	517670
Q8L828; Q9C827; Q9CAA0	4;4;2;1;1	Coatomer subunit beta-3;Coatomer subunit beta-2;Coatomer subunit beta-1		5	4	5.5	102.48	909	35.114	5189900	4922000	5513000	5825100	4630500	6389100	6200100	6456400	6678500	7491300
Q8LA13; Q84W89; Q9M2F9; P46942; Q75HJ0; Q6Z4K6; Q5N7W4;Q5VQL1;Q5JKF2;Q8W4R3;Q8H136;Q9LYJ9;Q9SQV1	1;1;1;0;0;0;0;0;0;0;0;0;0	DEAD-box ATP-dependent RNA helicase 11;DEAD-box ATP-dependent RNA helicase 37;DEAD-box ATP-dependent RNA helicase 52;DEAD-box ATP-dependent RNA helicase 30;DEAD-box ATP-dependent RNA helicase 14;DEAD-box ATP-dependent RNA helicase 46;DEAD-box ATP-dependent RNA helicase 40	RH11; RH37; RH52; RH30; RH14; RH46; RH40	13	1	2	66.025	612	18.026	0	0	0	0	0	0	0	0	0	2524600

Q8LAC4	1	Probable uridine nucleosidase 2	URH2	1	1	3.4	34.667	322	7.7336	0	0	0	0	0	0	0	0	0	773300
Q8LBZ7; Q8LB02	1;1	Succinate dehydrogenase [ubiquinone] iron-sulfur subunit 1, mitochondrial;Succinate dehydrogenase [ubiquinone] iron-sulfur subunit 2, mitochondrial	SDH2-1;SDH2-2	2	1	4.3	31.171	279	8.2073	0	0	0	0	0	0	0	0	0	556020
Q8LE58	1	ESCRT-related protein CHMP1A	CHMP1A	1	1	6.4	22.763	203	6.6078	0	0	0	0	0	0	0	0	494130	0
Q8LEM7	1	Calcineurin B-like protein 3	CBL3	1	1	7.5	26.031	226	10.48	0	0	0	0	0	0	0	0	55131	0
Q8LF48; Q570C8	1;1	3-ketoacyl-CoA thiolase 1, peroxisomal;3-ketoacyl-CoA thiolase 5, peroxisomal	KAT1; KAT5	2	1	2	46.611	443	5.8136	0	0	0	0	0	0	0	0	0	318000
Q8LFC0; O81796; P93032	2;2;1	Isocitrate dehydrogenase [NAD] regulatory subunit 1, mitochondrial;Isocitrate dehydrogenase [NAD] regulatory subunit 3, mitochondrial;Isocitrate dehydrogenase [NAD] regulatory subunit 2, mitochondrial	IDH1; IDH3; IDH2	3	2	6	39.626	367	20.455	4002700	3797700	4496100	3906600	3756600	5949500	4538700	6058800	6571400	6500100
Q8LFT2; Q8S944	1;1	Dynamin-related protein 3B;Dynamin-related protein 3A	DRP3B;DRP3A	2	1	1.3	86.643	780	6.4949	0	0	0	0	0	0	0	0	0	614030
Q8LSN3	1			1	1	5.3	34.871	303	8.5058	0	0	0	0	0	0	0	0	0	455430
Q8RUF8	2	Omega-amidase,chloroplastic	NLP3	1	2	7.6	40.33	369	34.572	0	1903100	1801000	0	0	1415700	1207300	1115000	1246100	1055600

Q8RWA7;O49313	1;1	NADH dehydrogenase [ubiquinone] 1 alpha subcomplex subunit 13-A;NADH dehydrogenase [ubiquinone] 1 alpha subcomplex subunit 13-B	MEE4	2	1	12.6	16.125	143	10.829	0	0	0	0	0	0	0	0	0	401660
Q8RWF0;Q8GYA6	1;1	26S proteasome non-ATPase regulatory subunit 13 homolog A;26S proteasome non-ATPase regulatory subunit 13 homolog B	RPN9A;RPN9B	2	1	3.1	44.167	386	19.646	1408500	1388900	1343900	0	0	2351100	2376500	2383500	3249500	2092800
Q8RWN9	2	Dihydrolipoyllysine-residue acetyltransferase component 2 of pyruvate dehydrogenase complex, mitochondrial		1	2	3.9	58.467	539	13.664	10527000	11560000	10950000	11279000	10476000	10884000	10219000	9979700	10818000	10722000
Q8RXK6;Q7XMK8;Q6H7S2;Q109G2;Q9M2E0;Q94BV4	2;1;1;1;1;1	DEAD-box ATP-dependent RNA helicase 8;DEAD-box ATP-dependent RNA helicase 12;DEAD-box ATP-dependent RNA helicase 6	RH8;RH12;RH6	6	2	6.1	57.686	505	18.428	0	0	0	0	0	0	0	0	716940	0
Q8S920;Q8S919;P35135;Q94F47;P35134;P35133;P35132;P35131	1;1;1;1;1;1;1;1	Ubiquitin-conjugating enzyme E2 28;Ubiquitin-conjugating enzyme E2 11;Ubiquitin-conjugating enzyme E2 10;SUMO-conjugating enzyme UBC9;Ubiquitin-conjugating enzyme E2 8	UBC28;UBC11;UBC10;UBC9;UBC8	8	1	18.4	16.573	147	7.1591	0	0	0	0	0	0	0	0	0	832130

Q8S9J8; Q8LF20	1;1	Probable clathrin assembly protein At4g32285;Putative clathrin assembly protein At2g25430		2	1	1.6	70.554	635	6.9118	0	0	0	0	0	0	0	0	0	740510
Q8S9Z2	1			1	1	4.5	44.332	398	22.886	0	0	0	0	0	0	0	0	0	934340
Q8SAG3; Q9ZSK2	1;1	Actin-depolymerizi ng factor 6	ADF6	2	1	11.2	16.551	143	8.1565	0	0	0	0	0	0	0	0	0	1875100
Q8VWK 0;Q9FIB 6	2;1	26S proteasome non-ATPase regulatory subunit 12 homolog B;26S proteasome non-ATPase regulatory subunit 12 homolog A	RPN5 B;RP N5A	2	2	5.4	51.117	442	12.077	1440600	1335300	1415400	1555200	1480200	1312900	1392700	1559500	1325000	1182500
Q8VXX5 ;Q9FHN6	2;1	Monocopper oxidase-like protein SKS1;Monocopper oxidase-like protein SKS2	SKS1; SKS2	2	2	4.4	65.876	589	15.398	5931700	5760100	0	5754300	0	6551600	7187400	7178500	0	8330200
Q8VYN6	1	ATP-dependent 6-phosphofructokin ase 5, chloroplastic	PFK5	1	1	2.4	58.614	537	10.033	0	0	0	0	0	0	0	0	0	718500
Q8VYR5	1	Pentatricopeptide repeat-containing protein At4g35850, mitochondrial		1	1	2.9	50.456	444	12.659	0	0	0	0	0	0	132090	0	0	0
Q8VYV7	1	3-dehydroquinate synthase, chloroplastic	DHQS	1	1	2.3	48.064	442	6.831	0	0	0	0	0	0	0	0	0	1142300
Q8VZC3	1	Delta-1-pyrroline-5 -carboxylate dehydrogenase 12A1, mitochondrial	ALDH 12A1	1	1	2	61.772	556	8.1719	0	0	0	0	0	0	0	0	0	270160
Q8VZH2	3;1	Aminopeptidase M1	APM1	2	3	4	98.178	879	20.83	3472400	3635800	3512800	0	4075900	2952000	3775900	4559500	4006200	4023400
Q8W246	1	Zinc transporter 7	ZIP7	1	1	7.7	39.378	365	5.9944	0	0	0	0	587560	0	0	0	0	0
Q8W4L0 ;Q9FYE4	1;1	Probable calcium-binding protein CML49;Probable calcium-binding	CML4 9;CM L50	2	1	4.5	35.302	335	12.364	0	0	0	0	0	0	0	0	0	1919300

		protein CML50																	
Q93V93	1	Peroxidase 44	PER44	1	1	4.2	33.805	310	6.4213	0	0	0	0	0	0	0	0	0	1482500
Q93VH9;Q8VYK6;P49204;O81363;P46299;P49398;O22424	1;1;1;0;0;0;0	40S ribosomal protein S4-1;40S ribosomal protein S4-3;40S ribosomal protein S4-2	RPS4A;RPS4D;RPS4B	7	1	3.8	29.802	261	18.087	0	0	0	0	0	1542800	0	0	0	0
Q93VR3;Q2R1V8;A3C4S4;A2Z7B3	5;4;4;4	GDP-mannose 3,5-epimerase		4	5	18.6	42.758	377	52.694	9423500<br>0	9427700<br>0	9726300<br>0	9984100<br>0	9313800<br>0	4982500<br>0	4862500<br>0	4774300<br>0	5195500<br>0	5344300<br>0
Q93Y40	1	Oxysterol-binding protein-related protein 3C	ORP3C	1	1	3.3	51.966	457	7.7117	0	0	0	0	0	0	0	0	0	4410100
Q93Z53;P55964;Q9FLW9	2;1;1	Plastidial pyruvate kinase 3, chloroplastic;Plastidial pyruvate kinase 2	PKP3;PKP2	3	2	5.6	62.614	571	50.86	0	0	0	1174800	1422600	3515700	5032300	5097900	3537800	3108500
Q93ZH0;Q6NPN4	1;1	LysM domain-containing GPI-anchored protein 1;LysM domain-containing GPI-anchored protein 2	LYM1;LYM3	2	1	2.9	43.489	416	9.8039	0	0	0	0	0	0	0	0	7072500	0
Q93ZN9	1	LL-diaminopimelate aminotransferase, chloroplastic	DAP	1	1	4.1	50.396	461	85.958	0	2027300	1640900	1752700	1548500	3369400	2718200	2871000	3110300	2907500
Q940J9	1	Probable methyltransferase PMT8		1	1	1.6	70.507	623	6.84	0	0	0	0	0	0	0	0	0	353460
Q940P8	2	T-complex protein 1 subunit beta	CCT2	1	2	8.9	57.285	527	96.056	1805400	1751200	1639500	1905400	2096100	8846500	9566100	9434200	7992300	7501200
Q940X7	1	RING-box protein 1a	RBX1A	1	1	11.9	13.238	118	7.9314	0	0	0	0	0	0	0	0	0	1105200
Q945F4;Q9AXQ4;P56336;Q9AXQ7;Q9AXQ	1;0;0;0;0;0;0;0;0	Eukaryotic translation initiation factor 5A-1;Eukaryotic translation	ELF5A-1;ELF5A-3;ELF5A-2	10	1	15.1	17.284	159	35.981	0	0	272580	185480	226490	1944400	2223100	2082700	2169600	1584500

5;Q9AXJ4;P56335;Q9XI91; Q9C505; Q93VP3		initiation factor 5A-3;Eukaryotic translation initiation factor 5A-2																	
Q945K7	1	Isocitrate dehydrogenase [NAD] catalytic subunit 5, mitochondrial	IDH5	1	1	3.7	40.624	374	54.979	3186100	3106300	3151600	3112300	3051400	5068400	8431700	7887300	5435600	5837100
Q945M1	1	NADH dehydrogenase [ubiquinone] 1 beta subcomplex subunit 9	CIB22	1	1	12.8	13.616	117	9.1963	0	0	0	0	0	0	0	0	0	1172500
Q949X7; Q94A94	4;4;1	Diaminopimelate decarboxylase 1, chloroplastic;Diaminopimelate decarboxylase 2, chloroplastic	LYSA1;LYSA2	3	4	13	53.557	484	36.987	2500700	1897700	2148600	2686600	2737600	4316400	4731100	5331100	4028000	4629600
Q949Y3	1	Bifunctional purple acid phosphatase 26;Acid phosphatase;Peroxidase	PAP26	1	1	4.2	55.009	475	74.199	0	0	0	0	0	0	0	0	0	2692400
Q94A08	1	Probable galactinol--sucrose galactosyltransferase 2	RFS2	1	1	1.8	85.142	773	96.401	0	0	0	0	0	0	0	0	0	906260
Q94A52	1	DEAD-box ATP-dependent RNA helicase 2	RH2	1	1	3.9	45.844	408	13.054	0	0	0	0	0	0	0	0	0	1518600
Q94A97; Q9FZ48	4;3	Ubiquitin-conjugating enzyme E2 35;Ubiquitin-conjugating enzyme E2 36	UBC35;UBC36	2	4	43.1	17.192	153	92.741	723770	685810	832020	718250	765020	1016400	1320800	1213000	1234000	1233800
Q94AZ4; Q8VZ50	2;2	Probable calcium-binding protein CML13;Probable	CML13;CML14	2	2	12.2	16.517	148	12.964	11576000	11081000	11850000	11993000	10478000	11992000	12330000	12863000	12780000	12522000

		calcium-binding protein CML14																	
Q94CD1	1	Omega-hydroxypal mitate O-feruloyl transferase	HHT1	1	1	1.8	50.957	457	5.9783	0	0	0	0	0	0	0	0	0	164150
Q94F62; Q94AG2; Q9XIC7; Q8LPS5; Q9SKG5	2;2;2;1; 1	BRASSINOSTER OID INSENSITIVE 1-associated receptor kinase 1;Somatic embryogenesis receptor kinase 1;Somatic embryogenesis receptor kinase 2;Somatic embryogenesis receptor kinase 5;Somatic embryogenesis receptor kinase 4	BAK1 ;SERK 1;SER K2;SE RK5;S ERK4	5	2	4.7	68.16	615	14.755	0	0	0	0	0	0	0	0	844930	0
Q94JN3	1			1	1	14.5	14.042	131	17.409	4223000	4172000	3784700	3838000	4396400	1106400 0	1000500 0	2061400 0	2249600 0	1023000 0
Q94JQ4	1	Reactive Intermediate Deaminase A, chloroplastic	RIDA	1	1	8	19.816	187	7.3848	0	0	0	0	0	0	0	0	0	877090
Q94JX9	2	Nascent polypeptide-associated complex subunit alpha-like protein 2		1	2	14.3	23.712	217	189.59	4568900 0	4543600 0	4230900 0	4523000 0	4424500 0	3159400 0	3379400 0	3359100 0	2874000 0	2933700 0
Q94K05	2	T-complex protein 1 subunit theta	CCT8	1	2	4.9	58.939	549	12.586	7383400	7154900	0	7499600	0	0	7168600	7571500	7373500	7876100
Q94KU1	2			1	2	6.8	53.244	483	83.555	7247800	7651400	6908500	5742900	6482000	8237000	7575200	8966500	8643900	8151500
Q94KU2	3;0			2	3	9.1	58.293	537	101.69	1447100 0	1369100 0	1623500 0	1435400 0	1349200 0	7818400	9689400	6831700	8747900	7378100
Q96007; Q36664; P60160;P 60159;P1 8630;P16	1;1;1;1; 1;1;1;1; 1	NADH-ubiquinone oxidoreductase chain 3	ND3	9	1	12.7	13.791	118	6.935	0	0	0	0	0	0	0	0	0	838020

265;P681<br>60;P6815<br>9;P92533																			
Q96283	1	Ras-related protein RABA2c	RABA<br>2C	1	1	8.3	23.849	217	22.705	0	0	0	0	0	0	0	0	0	758310
Q96300;<br>P42645;<br>Q96450	1;1;1	14-3-3-like protein GF14 nu;14-3-3-like protein GF14 upsilon	GRF7;<br>GRF5	3	1	6.4	29.824	265	30.575	2588500<br>0	2467500<br>0	3040300<br>0	2554500<br>0	2315400<br>0	2786900<br>0	2956200<br>0	2543100<br>0	2983900<br>0	2860700<br>0
Q96361;<br>P40940;<br>A8ISN6	2;2;1	ADP-ribosylation factor 3	ARF3	3	2	14.8	20.256	182	13.108	0	0	0	0	0	0	0	0	0	935100
Q96451;<br>O49996;<br>P93207;P<br>48349;P4<br>8348	3;2;2;2;<br>2	14-3-3-like protein GF14 lambda;14-3-3-like protein GF14 kappa	GRF6;<br>GRF8	5	3	15.8	27.921	247	131.91	2108800	1939800	1948900	810930	1823800	2518700<br>0	9202300	1292300<br>0	1092400<br>0	2011000<br>0
Q96481	1			1	1	4.8	37.271	336	5.8639	0	0	0	0	726120	0	0	0	0	0
Q96510	1	Peroxidase 35	PER35	1	1	3.6	35.756	329	25.275	0	0	0	0	0	0	0	0	0	3588500
Q96518;<br>Q96522	2;2	Peroxidase 16;Peroxidase 45	PER16<br>;PER4<br>5	2	2	6.8	35.832	323	17.449	4197500	4273900	4221800	4541900	4144800	7666400	7901000	1346300<br>0	7646800	1215000<br>0
Q96558;<br>Q2QS14;<br>Q2QS13;<br>Q75GS4;<br>B7F958;<br>Q9LF33;<br>Q9FM01;<br>Q9FZE1	1;0;0;0;<br>0;0;0;0	UDP-glucose 6-dehydrogenase 3;UDP-glucose 6-dehydrogenase 4;UDP-glucose 6-dehydrogenase 1	UGD3<br>;UGD<br>4;UG<br>D1	8	1	3.1	52.941	480	41.438	550180	557980	614020	563980	579140	3902200	4609600	3906100	3867000	3542600
Q9AT34;<br>Q00332;<br>Q9LX88;<br>P42798	1;1;1;1	40S ribosomal protein S15a-4;40S ribosomal protein S15a-1	RPS15<br>AD;R<br>PS15A<br>A	4	1	10.8	14.818	130	15.181	0	0	0	0	0	0	0	0	0	3538400
Q9ATF5;<br>Q943F3;<br>Q9LUD4	1;1;1	60S ribosomal protein L18a-3	RPL18<br>AC	3	1	3.9	21.407	178	6.4991	0	0	0	0	0	0	0	0	0	0
Q9AWB<br>2;O82802	1;1			2	1	4.2	63.821	573	26.28	0	0	0	0	0	0	0	0	0	1204700
Q9C522;<br>Q93VT8	2;1	ATP-citrate synthase beta chain	ACLB<br>-1	2	2	4.1	65.813	608	59.45	2137900	1900900	2484700	1769800	1703300	0	0	0	1487500	1694500

		protein 1																	
Q9C5I1; Q5W915; Q09WE7 ;Q0GZS3	2;1;1;1	UDP-sugar pyrophosphorylase	USP	4	2	4.9	67.851	614	12.528	0	0	0	0	0	1221400	0	0	0	0
Q9C5U3; Q94BQ2	2;2	26S protease regulatory subunit 8 homolog A;26S protease regulatory subunit 8 homolog B	RPT6 A;RPT 6B	2	2	7.6	47.247	419	15.954	0	0	0	0	0	4848300	4910800	5091300	5683600	4544800
Q9C5Z2	1	Eukaryotic translation initiation factor 3 subunit H	TIF3H 1	1	1	3	38.372	337	6.4208	0	0	0	0	0	0	0	0	0	1664100
Q9C6D2	1	Methylthioribose kinase	MTK	1	1	2.4	48.07	420	6.012	0	0	0	0	0	0	0	0	0	666920
Q9C8J2	1	NifU-like protein 5, mitochondrial	NIFU5	1	1	4.4	29.971	275	7.7861	0	0	0	0	0	0	0	0	0	1360800
Q9C8P0; Q9SQI8	1;1	Dihydrolipoyllysin e-residue acetyltransferase component 5 of pyruvate dehydrogenase complex, chloroplastic;Dihyd rolipoyllysine-resid ue acetyltransferase component 4 of pyruvate dehydrogenase complex, chloroplastic	EMB3 003;L TA2	2	1	3.2	48.306	465	7.123	0	0	0	0	0	0	0	0	0	819460
Q9C8Z4	1	Uncharacterized protein At3g06530		1	1	0.5	246.32	2197	6.2435	0	0	0	0	0	0	0	0	0	7693400
Q9C9C4	2	Enolase 1, chloroplastic	ENO1	1	2	7.5	51.474	477	26.704	2352000	2929800	2587700	2179500	2193600	2620200	2247400	0	2442400	0
Q9C9W4	1	Tapetum-specific methyltransferase 1	TSM1	1	1	5.2	26.35	233	5.8173	0	0	0	0	0	0	0	0	0	1056100
Q9CAR7	1	Hypersensitive-ind uced response	HIR2	1	1	5.6	31.405	286	12.353	0	0	0	0	0	0	0	0	0	2029300

		protein 2																	
Q9CB01	1	Ras-related protein RABF1	RABF1	1	1	5.4	21.889	202	7.6359	0	0	0	0	0	0	0	90404	0	0
Q9FFS8	1	40S ribosomal protein S10-2	RPS10B	1	1	8.3	19.733	180	7.7595	0	0	0	0	0	0	0	0	0	1050700
Q9FGR0	1	Cleavage and polyadenylation specificity factor subunit 1	CPSF160	1	1	0.6	158.07	1442	7.0571	0	0	0	0	426210	0	0	0	0	0
Q9FGX1	1	ATP-citrate synthase beta chain protein 2	ACLB-2	1	1	2.8	65.827	608	10.458	0	0	0	0	0	0	0	0	0	2121400
Q9FHK7	1	Probable leucine-rich repeat receptor-like protein kinase At5g05160		1	1	1.9	70.563	640	6.5933	0	0	0	0	0	0	0	0	0	421930
Q9FHW7	1	SKP1-like protein 1B	SKP1B	1	1	8.8	19.097	171	7.0361	0	0	0	0	0	0	0	0	0	971090
Q9FI53	1	Fumarate hydratase 2, chloroplastic	FUM2	1	1	3.4	54.083	499	5.9373	0	0	0	0	0	0	0	0	0	876480
Q9FJA6;Q9M339;Q9SIP7	2;2;2	40S ribosomal protein S3-3;40S ribosomal protein S3-2;40S ribosomal protein S3-1	RPS3C;RPS3B;RPS3A	3	2	10.1	27.458	248	13.685	0	0	0	3976100<br>0	5088300<br>0	8736400	0	1256100<br>0	1634800<br>0	1803100<br>0
Q9FJN8	1	Ras-related protein RABA4a	RABA4A	1	1	5.8	24.842	226	6.0366	0	0	0	0	0	0	0	0	0	590200
Q9FK53;Q8VZT0	1;1	H/ACA ribonucleoprotein complex subunit 1-like protein 2;Putative H/ACA ribonucleoprotein complex subunit 1-like protein 1		2	1	8.5	20.313	189	8.1691	0	0	0	0	0	0	0	0	0	442020
Q9FK68;Q39222	1;1	Ras-related protein RABA1c;Ras-related protein RABA1b	RABA1C;RABA1B	2	1	6.9	23.87	216	7.8131	0	0	0	0	0	0	0	0	0	771210
Q9FKK7	2	Xylose isomerase	XYLA	1	2	5.7	53.719	477	21.584	3031500	2507900	2984900	2983000	3088400	2087600	1516600	1340600	1410000	1584300
Q9FL69	1	Probable ADP-ribosylation factor GTPase-activating protein AGD5	AGD5	1	1	1.7	52.592	483	5.8061	0	0	0	0	0	0	0	0	539270	0
Q9FLF0;	2;2	40S ribosomal	RPS9	2	2	5.1	23.16	197	13.182	3994300	4114300	5764300	5566400	5872400	0	0	0	4028500	1365800

Q9LXG1		protein S9-2;40S ribosomal protein S9-1	C;RPS9B							0	0	0	0	0					
Q9FLH8	1	Probable fructokinase-7		1	1	3.2	37.028	343	7.7397	0	0	0	0	0	0	0	0	0	13820000
Q9FLX7	2	Probable NADH dehydrogenase [ubiquinone] 1 alpha subcomplex subunit 5, mitochondrial		1	2	21.9	19.179	169	15.238	0	0	0	3315800	1971800	11070000	11521000	10416000	11043000	10556000
Q9FM19	2	Hypersensitive-induced response protein 1	HIR1	1	2	9.8	31.431	286	19.207	0	1068600	901750	938260	642920	3297800	5248900	3361700	2216100	2835800
Q9FMV1;Q9SMN1	1;1	Gamma carbonic anhydrase-like 1, mitochondrial;Gamma carbonic anhydrase-like 2, mitochondrial	GAMMACAL1;GAMMACAL2	2	1	4.4	27.569	252	7.5687	0	0	0	0	0	0	0	0	0	776040
Q9FNX5;Q8S3C9;Q8LF21	2;1;1	Dynamin-related protein 1E;Dynamin-related protein 1D;Dynamin-related protein 1C	DRP1E;DRP1D;DRP1C	3	2	3.4	69.803	624	12.368	1911300	1615500	2278600	3100600	2236500	0	0	0	0	0
Q9FQ25	2	Protein TONNEAU 1a	TON1A	1	2	11.2	29.334	260	13.963	0	0	0	0	0	1269700	1260500	1215400	1155100	1034000
Q9FS87;Q9SWG0	1;1	Isovaleryl-CoA dehydrogenase, mitochondrial	IVD	2	1	2.9	45.263	412	6.7644	0	0	0	0	0	0	0	0	0	1032300
Q9FT36;P22275	1;0			2	1	4	49.64	451	254.24	0	0	0	0	0	0	0	0	0	2050800
Q9FT45;Q8RWC5	1;1	Fasciclin-like arabinogalactan protein 15;Fasciclin-like arabinogalactan protein 16	FLA15;FLA16	2	1	4.6	48.072	436	14.447	0	0	0	0	0	0	0	0	0	2003900
Q9FT97	2	Alpha-galactosidase 1	AGAL1	1	2	5.9	45.709	410	22.194	4117400	3995600	4207600	4243800	3493800	5389100	6219100	6202300	0	0
Q9FV65	1;0;1;1;1			5	1	1	110.64	967	64.655	0	0	0	0	0	0	0	0	0	2059600
Q9FVT2;O04487	2;1	Probable elongation factor 1-gamma 2;Probable		2	2	9	46.4	413	50.847	3000000	3438500	2417900	2758500	2410400	3058700	2378900	3027000	2739000	2237300

		elongation factor 1-gamma 1																	
Q9FWX8	1	ABC transporter B family member 12	ABCB 12	1	1	0.9	136.77	1273	43.331	0	0	0	0	0	0	0	0	0	408280
Q9FX32; Q6K973	1;1	Sucrose synthase 6	SUS6	2	1	1.5	106.87	942	14.238	0	0	0	0	0	0	0	0	0	324340
Q9FXT4; Q8VXZ7	1;1	Alpha-galactosidas e 3	AGAL 3	2	1	2.4	45.821	417	6.6978	0	0	0	0	0	0	0	0	0	6626300
Q9FYB7	1	Serine/arginine-rich splicing factor RS2Z32	RS2Z3 2	1	1	3.9	31.823	284	6.195	0	0	0	0	0	0	0	0	0	350200
Q9LD43	1	Acetyl-coenzyme A carboxylase carboxyl transferase subunit alpha, chloroplastic	CAC3	1	1	1.4	85.305	769	5.976	0	0	0	0	0	0	0	0	0	662950
Q9LDQ7	1			1	1	6.1	42.8	393	7.978	3185900	3556300	3083100	3234300	0	6481400	6457800	9303400	1046000<br>0	1041300<br>0
Q9LEI9	1			1	1	3.8	47.913	445	10.481	2586700	2518300	4635600	2898400	3088600	2803400	2756200	2665000	2551900	2722700
Q9LFH5; Q9FF52; P50883	2;2;2	60S ribosomal protein L12-2;60S ribosomal protein L12-3;60S ribosomal protein L12-1	RPL12 B;RPL 12C;R PL12 A	3	2	11.4	17.97	166	17.89	8629200	0	9618300	0	0	0	5278600	6364700	6253900	0
Q9LFN6; Q56XG6	1;1	DEAD-box ATP-dependent RNA helicase 56;DEAD-box ATP-dependent RNA helicase 15	RH56; RH15	2	1	4.4	48.337	427	6.7157	0	0	0	0	0	0	0	0	0	1372100
Q9LFT9; Q9SID8; Q9SMR4	1;0;0;0	Ras-related protein RABH1e;Ras-relat ed protein RABH1d;Ras-relat ed protein RABH1c	RABH 1E;RA BH1D ;RAB H1C	4	1	5.8	23.133	207	7.0071	0	0	0	0	0	0	0	0	0	1294700
Q9LHE9	1	Myosin-1	VIII-1	1	1	1.3	131.13	1166	7.3462	0	0	0	0	0	0	0	0	0	0
Q9LIA8	1	UDP-glucose 6-dehydrogenase 2	UGD2	1	1	3.1	53.172	480	21.795	0	0	0	0	0	0	0	0	0	5537100
Q9LIK0; Q40545	1;0	Plastidial pyruvate kinase 1, chloroplastic	PKP1	2	1	3	65.13	596	13.658	0	0	0	0	0	0	0	0	0	1026800
Q9LIK9	2	ATP sulfurylase 1,	APS1	1	2	5.4	51.458	463	23.273	1316100	4922900	1094300	0	4737800	1450400	1426100	1468500	0	1504100

		chloroplastic								0		0							
Q9LJA0	2	Putative inactive cysteine synthase 2	OASA2	1	2	13.3	19.638	188	11.643	2169800	2085800	2072000	2129800	1976400	7649000	3966800	3856200	3767400	3910900
Q9LJI5; Q9LHA4;Q8RU33	2;2;1	V-type proton ATPase subunit d1;V-type proton ATPase subunit d2	VHA-d1;VHA-d2	3	2	9.7	40.791	351	12.704	0	0	801790	0	0	2270300	2173100	3455400	0	1970000
Q9LKG7	1			1	1	1.9	51.55	471	12.951	0	0	0	0	0	0	0	0	0	5448000
Q9LM92	1	Small nuclear ribonucleoprotein SmD3b	SMD3B	1	1	11.5	14.237	131	6.7897	0	0	0	0	0	0	0	0	0	212240
Q9LNL0	1	Peroxidase 8	PER8	1	1	2.9	34.331	310	6.373	0	0	0	0	0	0	0	0	0	1574300
Q9LQ55	1	Dynamin-2B	DRP2B	1	1	1.1	100.23	920	6.7468	0	0	0	0	0	0	0	0	0	1545200
Q9LRS0	1	Peroxisomal (S)-2-hydroxy-acid oxidase GLO2	GLO2	1	1	2.7	40.306	367	6.8757	0	0	0	0	0	0	0	0	0	5730600
Q9LSU0	1			1	1	6.4	27.238	249	8.1599	0	0	0	0	0	0	0	0	0	811440
Q9LT08	2	26S proteasome non-ATPase regulatory subunit 14 homolog	RPN11	1	2	11.4	34.353	308	26.614	0	0	0	0	0	0	0	0	3953900	0
Q9LT69	1	D-3-phosphoglycerate dehydrogenase 3, chloroplastic	PGDH3	1	1	1.7	62.121	588	6.7733	0	0	0	0	0	0	0	0	0	3548600
Q9LT91	1	Peroxidase 66	PER66	1	1	2.8	35.559	322	6.3322	0	0	0	0	0	0	0	0	0	8448100
Q9LTX9; Q9STW6	2;1;1	Heat shock 70 kDa protein 7, chloroplastic;Heat shock 70 kDa protein 6, chloroplastic	HSP70-7;HSP70-6	3	2	6.1	76.996	718	50.761	2538200	2691300	2609300	2430000	2637500	1011700	1058500	1177400	1010400	1058200
Q9LU41; Q9LF79	2;1	Calcium-transporting ATPase 9, plasma membrane-type;Calcium-transporting ATPase 8, plasma membrane-type	ACA9;ACA8	2	2	2.3	118.77	1086	16.104	2046800	1881600	2082400	2056500	0	640980	652930	646955	0	0
Q9LUE6	1	Probable UDP-arabinopyranose mutase 4	RGP4	1	1	2.7	41.866	364	5.9769	0	0	0	0	786340	0	0	0	0	0

Q9LV21	4	T-complex protein 1 subunit delta	CCT4	1	4	14.4	57.775	536	40.051	4404200	4579600	4410200	2879600	4691900	5560500	5639600	5902900	5346000	5010600
Q9LV52	1	Heat stress transcription factor C-1	HSFC 1	1	1	4.5	37.714	330	6.4335	0	0	0	0	0	0	0	0	0	196090
Q9LVI9	1	Dihydropyrimidine dehydrogenase (NADP(+)), chloroplastic	PYD1	1	1	2.8	46.846	426	10.605	0	0	0	0	0	0	0	0	0	5928300
Q9LW64 ;P58728	1;1	Uncharacterized protein At3g23325;Uncharacterized protein At4g14342		2	1	14.9	10.177	87	10.202	0	0	0	0	0	0	0	0	0	502960
Q9LX08	1	Annexin D6	ANN6	1	1	3.1	36.569	318	9.5081	0	0	0	0	0	0	0	0	0	1323000
Q9LXC0; Q96254	1;1	Guanosine nucleotide diphosphate dissociation inhibitor At5g09550;Guanosine nucleotide diphosphate dissociation inhibitor 1	GDI1	2	1	3.1	49.549	445	20.651	1768600	2520700	2489000	2510700	1551400	849940	1050800	842000	839390	938400
Q9LXJ0; O82533	1;1	Cell division protein FtsZ homolog 2-2, chloroplastic;Cell division protein FtsZ homolog 2-1, chloroplastic	FTSZ2-2;FTSZ2-1	2	1	3.8	50.317	473	8.0579	0	0	0	0	0	0	0	0	0	1383300
Q9LY99; O04331; Q9LK25	1;1;1	Prohibitin-5, mitochondrial;Prohibitin-3, mitochondrial;Prohibitin-4, mitochondrial	PHB5; PHB3; PHB4	3	1	4	30.4	277	7.5532	2929000	2880100	2826200	2367000	2709400	5114200	3647400	4828200	4814600	4777800
Q9LYQ2 ;Q56YT0	2;1	Laccase-13;Laccase-3	LAC13;LAC3	2	2	8.3	63.144	569	10.885	0	0	0	0	0	0	0	0	0	2382200
Q9LZ06	1	Glutathione S-transferase L3	GSTL3	1	1	4.3	27.09	235	6.8978	0	0	0	0	0	0	0	0	0	4441100
Q9LZ82	1	Protein BTR1	BTR1	1	1	4.5	33.82	313	8.054	0	0	0	0	0	0	0	0	0	4111100

Q9M040;Q05326;Q05327;P51851;Q0D3D2;A2YQ76;P51850;Q10MW3;Q0DHF6;A2Y5L9;A2XFI3;P28516;P51846;Q9M039;Q9FFT4;O82647	2;1;1;1;1;1;1;1;1;1;1;1;1;1;1;1	Pyruvate decarboxylase 4;Pyruvate decarboxylase 3;Pyruvate decarboxylase 2;Pyruvate decarboxylase 1	PDC4;PDC3;PDC2;PDC1	16	2	3.3	65.464	603	11.157	1346600	0	0	4345900	0	0	0	0	0	0
Q9M069	1	Glucan endo-1,3-beta-glucosidase 7		1	1	2	53.121	504	5.9067	0	0	0	0	0	0	0	0	0	420930
Q9M076;Q9M0F9;Q9C5J7;Q94AA4;Q9FKG3	1;1;1;1;1	ATP-dependent 6-phosphofructokinase 6;ATP-dependent 6-phosphofructokinase 1;ATP-dependent 6-phosphofructokinase 7;ATP-dependent 6-phosphofructokinase 3;ATP-dependent 6-phosphofructokinase 4, chloroplastic	PFK6;PFK1;PFK7;PFK3;PFK4	5	1	2.8	50.787	462	11.559	0	0	0	0	0	0	0	0	0	1391200
Q9M0M4;Q9FGK3	1;1	Putative MO25-like protein At4g17270;Putative MO25-like protein At5g47540		2	1	2.6	39.65	343	6.2109	0	0	0	0	0	0	0	0	0	722580
Q9M1H3	1	ABC transporter F family member 4	ABCF4	1	1	2.1	80.48	723	5.8868	0	0	0	0	0	0	0	0	0	416830
Q9M1R2	1		T17J13.80	1	1	2.6	60.755	530	7.3581	0	0	0	0	0	0	0	0	0	676030
Q9M1S8	1	Probable glutamate	AMP1	1	1	3	77.152	705	6.8181	0	0	0	0	0	1762100	0	0	0	0

		carboxypeptidase 2																	
Q9M263	3;1;1;1;1;1;1;1;1	Germin-like protein subfamily 2 member 4	GLP10	9	3	11.4	23.55	220	20.042	30502000	29396000	31595000	9524900	9953200	1318800	4794400	7246400	9748100	13250000
Q9M2A0	1	ATPase 8, plasma membrane-type	AHA8	1	1	1.9	104.13	948	16.9	537350	438610	423950	416350	0	31696000	33644000	31076000	30026000	29095000
Q9M4S8;Q9SKP6	1;1;0	Triosephosphate isomerase, chloroplastic	TIM	3	1	5.4	33.526	314	27.538	0	0	0	0	0	0	0	0	0	153570
Q9M4T8;Q42134;O81149	3;3;3;1	Proteasome subunit alpha type-5-B;Proteasome subunit alpha type-5-A	PAE2;PAE1	4	3	19.8	25.98	237	115.16	6682800	9379300	5000500	4944900	7989900	27530000	24569000	24587000	22758000	20583000
Q9M571;Q9FR44;Q944H0;Q9C6B9	2;2;2;1	Phosphoethanolamine N-methyltransferase 1;Phosphomethylethanolamine N-methyltransferase;Phosphoethanolamine N-methyltransferase 3	NMT1;NMT2;NMT3	4	2	5.1	56.36	494	12.724	0	0	0	0	0	0	0	1868500	0	0
Q9M5K3;P31023;Q9SPB1;Q9M5K2	2;1;1;1	Dihydrolipoyl dehydrogenase 1, mitochondrial;Dihydrolipoyl dehydrogenase 2, mitochondrial	LPD1;LPD2	4	2	5.5	53.987	507	13.019	3433000	0	0	3120700	0	2937800	0	2854400	2934300	2776200
Q9M612;Q9LHG9	1;1	Nascent polypeptide-associated complex subunit alpha-like protein 1		2	1	6.3	22.408	205	6.7759	0	0	0	0	0	0	0	0	0	3622800
Q9M888;Q8L7N0	1;1	T-complex protein 1 subunit zeta 1;T-complex protein 1 subunit zeta 2	CCT6A;CCT6B	2	1	2.6	58.949	535	8.7219	0	0	0	0	0	0	0	0	0	1043200
Q9M8X6	1	Germin-like protein subfamily 1 member 6		1	1	5.7	24.921	227	6.8216	0	0	0	0	0	10786000	11684000	11914000	10829000	10413000

Q9M9K1;P35494	2;2;1	Probable 2,3-bisphosphoglycerate-independent phosphoglycerate mutase 2		3	2	3.4	60.763	560	49.977	0	0	12675000	19744000	22818000	0	0	0	0	0
Q9M9S2	1	Probable pectate lyase 3	AT59	1	1	3.5	51.454	459	5.9886	0	0	0	0	0	0	0	0	0	1301100
Q9MAB3	1	Probable nucleolar protein 5-2	NOP5-2	1	1	1.9	59.002	533	34.754	0	0	0	0	0	0	0	804830	0	0
Q9MAH0;Q84VW9	1;1	Phosphoenolpyruvate carboxylase 1;Phosphoenolpyruvate carboxylase 3	PPC1;PPC3	2	1	1.4	110.28	967	40.832	0	0	0	0	0	0	0	0	0	710730
Q9MAT0;O48844	4;4	26S proteasome non-ATPase regulatory subunit 1 homolog B;26S proteasome non-ATPase regulatory subunit 1 homolog A	RPN2B;RPN2A	2	4	5.3	108.86	1001	25.747	7324300	6821200	6864800	8815400	8012700	8032700	7781800	8133900	8946300	8523100
Q9MAY3	1			1	1	7.2	29.139	264	39.329	0	0	0	0	0	0	0	0	0	969950
Q9MB94;P54847;O65378	1;1;1	1-aminocyclopropane-1-carboxylate oxidase 3		3	1	5	36.242	319	6.7437	0	0	0	0	955080	0	0	0	0	0
Q9MTJ8	3	ATP-dependent Clp protease proteolytic subunit		1	3	33.3	27.956	249	85.579	0	2405800	2345400	2140000	2298400	5344000	3897700	6009200	3957100	3823700
Q9MTK5;Q70XY9;Q6L383;Q6EW36;Q6ENU7;Q6ENF6;Q4VZH5;Q332W1;Q2L925;Q1KXU2;P69390;P69388;P69387;P69386;P69383;	1;1;1;1;1;1;1;1;1;1;1;1;1;1;1;1;1;1;1;1;1;1;1;1;1;1	Cytochrome b559 subunit alpha	psbE	26	1	14.5	9.3864	83	7.3313	0	0	0	0	0	0	0	0	0	262440

P69382;P59702;P13554;P0C370;P0C369;P0C368;P05170;A1EA25;A1E9U1;A0ZZ52;P56779 Q9MTL3	1			1	1	2.3	59.317	517	10.625	0	0	0	0	0	0	0	0	0	1298600
Q9MTL7;B0Z5D4;B0Z4W6;B0Z4N2;B0Z550	1;1;1;1;1			5	1	2.2	55.246	505	7.1325	0	0	0	0	827380	0	0	0	0	0
Q9MTN2;B0Z5C3;B0Z539;B0Z4V5;B0Z4M1;Q9BBT1;Q3C1I3;Q33C41;Q09G50;Q09FW5;A8SE99;A0ZZ31;Q9XPS4;Q8S8X7;Q7YJX6;Q70Y07;Q6ENY0;Q6ENJ0;Q68S10;Q4FFN5;Q49L02;Q3V538;Q3BAP4;Q332Y2;Q2VEI0;Q2QD93;	4;4;4;4;4;3;3;3;3;3;3;3;3;3;3;3;3;3;3;3;3;3;3;3;3;3;3;3;3;3;3;3;3;3;3;3;3;3;3;3;3;3;3;3;3;3;3;3;3;3;3;3;3;3;3;3;3;3;3;3;3;3;3;3;3;3;3;3;3;3;3;3;3;3;3;3;3;3;3;3;3;3;3;3;2;2;2;2;2;2;2;2;2;2;2;2;2;2;2;2;2;2;2;2;	Photosystem II CP43 reaction center protein	psbC	109	4	11.2	51.821	473	25.466	7320100	6742600	7415700	7680400	7473600	0	0	0	0	0

Q2PMT9;Q2MIJ1;Q2MIA4;Q2L901;Q1KXW3;Q14FG1;Q0ZJ24;Q0G9W6;Q0G9M3;Q09X21;Q06RD5;Q06H01;Q06GR4;Q06FW0;P11095;P10804;P0C367;P0C366;P0C365;P0C158;P06413;P06004;P06003;B5LMM0;B3TNB9;B2XWK1;B2LMI9;B1NWE6;B1A931;A9LYI1;A8Y9F6;A8W3I3;A8W3C0;A7Y3C5;A7M962;A7M8Z7;A6MMU0;A6MMK3;A6MMB8;A6MM32;A4QLS9;A	2;1;1;1;1

4QLJ0;A4QLA2;A4QL15;A4QKS7;A4QKI8;A4QKA1;A4QK14;A4QJS9;A4QJJ5;A4QJB1;A4GYQ5;A4GGA1;A1E9Z4;A1E9R0;A0A331;Q09MI2;P56778;Q8WI20;B1NTP4;Q8M9W5;Q8HB52;Q85WW6;Q85FM3;Q85AU0;Q6YXN9;Q6EW52;Q5SCW5;Q32RS9;P41643;P06414;B2Y1U6;B1VKG9;A9L992;A6H5G7;A6BM30;A2T329;A1XFV1;P48187Q9MTP7;B0Z5B2;B0Z528;	2;2;2;2;2;1;1;1;1;1;1;1;0;0;0;0;0;0;0;0;0;0;0;0;0;0;0;0;0;0;0;0;0;0;0;0;0;0;0;0;0;0;0;0;0;0;0;0;0;0;0;0;0	53	2	6.6	53.531	498	42.595	2493300	2574000	2484100	2624400	2481500	1476900	1664800	0	0	766430

B0Z4U4;
B0Z4L0;
Q06RC2;
Q9MU41
;Q9MRV
8;Q9MR
R1;Q9M
RF3;Q49
KZ1;Q0Z
J13;Q9M
U82;Q9
MU80;Q
9MU43;
Q9MU30
;Q9MRQ
5;Q9MR
M0;Q9M
RI8;Q9B
A96;Q9B
A86;Q9B
A84;Q9B
A69;Q95
DR6;Q7
YJW5;Q
7HHY7;
Q7HHY5
;Q7HHY
4;Q7HH
X4;Q7H
HX1;Q7
HHW4;Q
7HDI4;Q
6QBP2;Q
09X10;Q
09G39;Q
09FV4;Q
06GQ3;A
8SEB0;A
6MMC9;
A0A342;
Q9TMV0
;Q9TMQ

4;A6MML4;Q9BA92;Q95FL8;Q95AF8;Q95AD7;Q14FF0;A4GYR7;Q9MU81;Q9BA85;B1A942;B1NWF7																			
Q9MUS6;Q0P3M5	1;1			2	1	1.6	76.777	668	7.2699	0	0	0	0	591180	0	0	0	0	0
Q9S726	1	Probable ribose-5-phosphate isomerase 3, chloroplastic	RPI3	1	1	6.9	29.305	276	6.436	0	0	0	0	0	0	0	0	0	299450
Q9S7A0	2	Probable glutamate dehydrogenase 3	GSH3	1	2	6.6	44.527	411	33.108	4547500	5626100	3961000	4749900	4262800	3004000	3055400	2886900	0	0
Q9S7D8	1	ATP sulfurylase 4, chloroplastic	APS4	1	1	1.5	52.125	469	5.7747	0	0	0	0	0	0	0	0	0	462510
Q9S7L9	1	Cytochrome c oxidase subunit 6b-1	COX6B-1	1	1	8.9	21.195	191	5.8623	0	0	0	0	0	0	0	0	0	264540
Q9S7Y7	1	Alpha-xylosidase 1	XYL1	1	1	1.1	102.4	915	6.8389	0	0	0	0	0	0	0	0	0	1954200
Q9S818;Q05963;Q05965;Q06942;Q05964;P28038	2;1;1;1;1;1	Naringenin,2-oxoglutarate 3-dioxygenase	F3H	6	2	8.4	40.275	358	40.619	0	971850	0	0	0	2316100	3564200	2343900	2206700	2231500
Q9SA78	1	Coatomer subunit epsilon-1		1	1	4.8	32.602	292	7.0857	767460	1025900	959650	968330	1030700	1727600	2998500	1674200	2515600	2474200
Q9SAJ6	1	Glyceraldehyde-3-phosphate dehydrogenase GAPCP1, chloroplastic	GAPCP1	1	1	4.7	44.83	422	175.35	4575300	4288100	3622800	3923800	3867100	4169600	4637400	4117600	4066500	4143700
Q9SAK4	1	Succinate-semialdehyde dehydrogenase, mitochondrial	ALDH5F1	1	1	2.3	56.558	528	10.975	0	0	0	0	0	0	0	0	0	4933100

Q9SC12	1			1	1	7.5	17.265	159	35.032	0	0	0	0	0	0	0	0	0	5074800
Q9SC19; Q6Z4G3; P80607	1;1;0			3	1	3	41.805	365	23.032	0	0	0	0	0	0	0	0	0	835960
Q9SCM3;Q93VB8;Q8L8Y0;P49688	2;2;2;2	40S ribosomal protein S2-4;40S ribosomal protein S2-2;40S ribosomal protein S2-1;40S ribosomal protein S2-3	RPS2D;RPS2B;RPS2A;RPS2C	4	2	5.8	30.116	276	12.848	0	0	0	0	8649300	0	0	0	0	0
Q9SCN8	1	Cell division control protein 48 homolog D	CDC48D	1	1	1.8	90.339	815	87.03	0	0	0	0	0	0	0	0	0	3548900
Q9SDG6; O48558; Q9M5M6;O49884; Q9LSA3; Q9C8F7; Q8VZ19	2;2;1;1;1;1;1	60S ribosomal protein L30-3;Putative 60S ribosomal protein L30-1;60S ribosomal protein L30-2	RPL30C;RPL30A;RPL30B	7	2	24.3	12.363	111	14.282	8535300	9260700	4984600	5852300	5292900	0	0	2709300	3202200	2534900
Q9SE94	1			1	1	3	66.428	593	31.471	0	0	0	0	0	0	0	0	0	1208200
Q9SEI3; Q9MAK9	5;5	26S protease regulatory subunit 10B homolog A;26S protease regulatory subunit S10B homolog B	RPT4A;RPT4B	2	5	15.8	44.816	399	37.92	4360100	4209900	4874700	4438000	5309900	7700300	6120600	6500200	7880800	7042400
Q9SEU4	1	Serine/arginine-rich SC35-like splicing factor SCL33	SCL33	1	1	3.1	33.337	287	6.0915	0	0	0	0	0	0	0	0	0	440250
Q9SF16	5	T-complex protein 1 subunit eta	CCT7	1	5	11.7	59.776	557	45.156	6952400	5997600	6888400	7685400	7053600	5338600	6067500	5687400	6119600	5909100
Q9SFU6; Q9SJM0	1;1	Callose synthase 9;Callose synthase 10	CALS9;CALS10	2	1	0.7	217.08	1890	6.0475	0	0	0	0	455510	0	0	0	0	0
Q9SG79	1	Protein SAD1/UNC-84 domain protein 2	SUN2	1	1	2.6	49.94	455	5.9019	0	0	0	0	0	0	0	2906100	0	0
Q9SGA6; Q9LF30; Q9FNP8	1;1;1	40S ribosomal protein S19-1;40S ribosomal protein	RPS19A;RPS19B;R	3	1	9.1	15.828	143	6.4213	0	0	0	0	0	0	0	0	0	4421100

		S19-2;40S ribosomal protein S19-3	PS19C																
Q9SGC1; P93805; O49299; P93804; Q9SNX2; Q9ZSQ4; Q9SM60; P93262	6;5;5;4;3;3;3;3;2	Probable phosphoglucomutase, cytoplasmic 2;Probable phosphoglucomutase, cytoplasmic 1		9	6	14.7	63.481	585	54.576	10869000	8928700	12713000	12407000	11368000	6913500	7641900	3540900	4620500	9240000
Q9SGW3	1	26S proteasome non-ATPase regulatory subunit 8 homolog A	RPN12A	1	1	6	30.706	267	17.776	0	0	0	0	0	0	0	0	0	1157100
Q9SH69; Q9FFR3; Q9LI00	1;0;0	6-phosphogluconate dehydrogenase, decarboxylating 1, chloroplastic;6-phosphogluconate dehydrogenase, decarboxylating 2, chloroplastic		3	1	3.7	53.377	487	21.583	0	0	0	0	0	0	0	0	0	1047300
Q9SHP0; Q96255; P52877	2;2;1	Phosphoserine aminotransferase 2, chloroplastic;Phosphoserine aminotransferase 1, chloroplastic	PSAT2;PSAT1	3	2	5.5	46.633	422	11.798	0	0	0	0	0	0	0	0	0	3301400
Q9SIF2	1		CR88	1	1	1.8	88.662	780	10.499	0	0	0	0	0	0	0	0	0	1022400
Q9SIV2; Q6XJG8	1;1	26S proteasome non-ATPase regulatory subunit 2 homolog A;26S proteasome non-ATPase regulatory subunit 2 homolog B	RPN1A;RPN1B	2	1	1.1	98.143	891	6.5981	0	0	0	0	0	0	0	0	0	4320600
Q9SJ44; Q9SVD7	2;2	Ubiquitin-conjugating enzyme E2 variant 1C;Ubiquitin-conjugating enzyme E2	UEV1C;UEV1D	2	2	24.1	16.481	145	24.33	930950	823930	904460	956620	940840	697450	1042400	1108200	2118800	1398500

		variant 1D																	
Q9SJB3	2	ATPase 5, plasma membrane-type	AHA5	1	2	1.4	104.74	949	16.048	12996000	13264000	11291000	11946000	11069000	5919100	5871300	4958900	5849500	5565200
Q9SJH7; P49299; Q9LXS7; Q9LXS6	2;1;1;1	Citrate synthase 3, peroxisomal;Citrate synthase 1, peroxisomal;Citrate synthase 2, peroxisomal	CSY3; CSY1; CSY2	4	2	5.3	52.895	480	13.61	3930500	3720900	3919700	4467000	3720300	1159000	1097000	1291700	1448600	1232100
Q9SKC5	1	UDP-glycosyltransferase 74D1	UGT74D1	1	1	4.2	51.053	456	7.3457	0	0	0	0	0	3019500	3054200	3639200	3106100	0
Q9SKC9	1	NADH dehydrogenase [ubiquinone] 1 beta subcomplex subunit 7		1	1	8.7	11.74	103	6.3701	0	0	0	0	0	0	0	0	0	5584400
Q9SLA8; P80030	2;1	Enoyl-[acyl-carrier-protein] reductase [NADH], chloroplastic	MOD1	2	2	7.4	41.213	390	38.256	1537600	2162000	1260500	1208300	1290800	4049100	4749700	2152500	2140600	2339200
Q9SM09; P49087; Q40002	1;0;0;0;0			5	1	2.4	68.681	623	72.957	0	0	0	0	0	5385800	5946700	5878000	5865700	7612700
Q9SM64; P35017; Q9LYK8	2;2;1	Superoxide dismutase [Mn] 2, mitochondrial	MSD2	3	2	7	25.455	228	17.014	10088000	9704300	10123000	19567000	8631500	2435900	3072200	5008500	7313900	7206500
Q9SMV6	1	Protein EXPORTIN 1A	XPO1	1	1	1	123.24	1075	7.6523	0	0	0	0	0	0	0	0	0	1445000
Q9SN35; O49513	1;1	Ras-related protein RABA1d;Ras-related protein RABA1e	RABA1D;RABA1E	2	1	4.7	23.869	214	15.773	0	0	0	0	0	0	0	0	0	2773500
Q9SN58; Q9T0A7	1;1	UDP-glucose 4-epimerase 5;UDP-glucose 4-epimerase 2	UGE5; UGE2	2	1	2.3	38.617	351	14.143	0	0	0	0	0	0	0	0	0	19381000
Q9SN68; P31582	1;1	Ras-related protein RABF2b;Ras-related protein RABF2a	RABF2B;RABF2A	2	1	5.5	21.873	200	6.0697	0	0	0	0	0	0	0	0	0	2539000
Q9SN95; Q9FIE8; Q9ZV36	1;1;1	UDP-glucuronic acid decarboxylase 5;UDP-glucuronic acid decarboxylase 3;UDP-glucuronic	UXS5; UXS3; UXS6	3	1	3.2	38.389	341	6.7597	0	0	0	0	0	0	0	0	0	2510000

		acid decarboxylase 6																	
Q9SP07; P93259; O49997; P42652	1;1;1;1			4	1	6.6	29.253	259	30.167	37785000	36062000	38010000	30888000	35466000	31403000	29784000	32327000	31528000	32763000
Q9SPE6	1	Alpha-soluble NSF attachment protein 2	ASNAP2	1	1	4.2	32.755	289	7.1623	0	0	0	0	0	0	0	0	0	3956800
Q9SPP9	1			1	1	2	60.933	540	6.16	0	0	0	0	0	0	0	0	0	452210
Q9SQT8	1	Bifunctional 3-dehydroquinate dehydratase/shikimate dehydrogenase, chloroplastic;Dehydroquinate dehydratase;Shikimate dehydrogenase	EMB3004	1	1	1.5	65.795	603	6.3661	0	0	0	0	0	0	0	0	0	3269900
Q9SRH6	1	Hypersensitive-induced response protein 3	HIR3	1	1	3.5	31.321	285	6.9979	0	0	0	0	0	0	0	0	0	3757300
Q9SRI1; O64740	1;1	Protein transport protein SEC13 homolog A;Protein transport protein SEC13 homolog B	SEC13A;SEC13B	2	1	4	32.63	302	6.2354	0	0	0	0	0	0	0	0	1517400	0
Q9SRT9; Q9LFW1	1;1	UDP-arabinopyranose mutase 1;UDP-arabinopyranose mutase 2	RGP1; RGP2	2	1	3.6	40.629	357	102.7	0	0	0	0	0	0	0	0	0	1671700
Q9SRV5	1	5-methyltetrahydropteroyltriglutamate--homocysteine methyltransferase 2	MS2	1	1	2.4	84.583	765	22.454	865270	769800	614190	695170	730450	17636000	18398000	16047000	17534000	16309000
Q9SRZ6	2;1	Cytosolic isocitrate dehydrogenase [NADP]	CICDH	2	2	6.6	45.746	410	225.26	12784000	11022000	14795000	15604000	10483000	16562000	22159000	20443000	17469000	13650000
Q9STG6	1	Deoxyuridine 5-triphosphate nucleotidohydrolase	DUT	1	1	10.2	17.557	166	7.3221	0	0	0	0	0	0	0	0	0	1284300
Q9SU63	2	Aldehyde	ALDH	1	2	5.2	58.588	538	50.329	1824500	1577100	1583200	1397500	1517700	8671600	9181600	9253500	9174600	9915800

		dehydrogenase family 2 member B4, mitochondrial	2B4							0	0	0	0	0					
Q9SUE0; Q9SRS3	1;1		T13J8.100;F21O3.14	2	1	5	23.705	218	6.2538	0	0	0	0	0	0	0	0	286330	0
Q9SV21; Q9SV20	1;1	Coatomer subunit beta-1;Coatomer subunit beta-2		2	1	0.9	106.14	948	6.373	0	0	0	0	0	0	0	0	0	1383400
Q9SVC2	1	Syntaxin-122	SYP122	1	1	3.8	37.836	341	6.9263	0	0	0	0	0	0	0	0	0	1709900
Q9SVR0; Q9SFU1; Q9LRX8; Q9FKC0	1;1;1;1	60S ribosomal protein L13a-3;60S ribosomal protein L13a-1;60S ribosomal protein L13a-2;60S ribosomal protein L13a-4	RPL13AC;RPL13AA;RPL13AB;RPL13AD	4	1	5.8	23.629	206	7.9345	0	0	0	0	0	0	0	0	0	482290
Q9SW96; Q9SSK1	2;1	Asparagine--tRNA ligase, cytoplasmic 1;Asparagine--tRNA ligase, cytoplasmic 3	SYNC1;SYNC3	2	2	4	63.782	572	16.439	1529400	0	0	0	1029300	1781100	1664600	1804100	1811800	0
Q9SWE7	1			1	1	3.9	26.343	230	6.3868	0	0	0	0	0	0	0	0	0	474290
Q9SXF8; Q9AR14	1;1			2	1	8.7	30.756	288	5.9411	0	0	0	0	0	0	0	133490	0	0
Q9SXU1; Q6YT00; A2YXU2;O24030; Q0J006; A2Z3I9;P30186;O24616	4;3;3;3;2;2;2;2	Proteasome subunit alpha type-7-A;Proteasome subunit alpha type-7-B	PAD1;PAD2	8	4	26.1	27.096	249	119.29	31425000	30205000	28503000	19660000	26229000	20116000	16579000	22774000	16485000	14557000
Q9SYM5;Q9LH76;Q9LPG6	2;1;1	Trifunctional UDP-glucose 4,6-dehydratase/UDP-4-keto-6-deoxy-D-glucose 3,5-epimerase/UDP	RHM1;RHM3;RHM2	3	2	4.2	75.371	669	15.866	0	0	0	0	0	0	0	0	2119100	0

-4-keto-L-rhamnos
e-reductase
RHM1;UDP-glucos
e
4,6-dehydratase;U
DP-4-keto-6-deoxy
-D-glucose
3,5-epimerase/UDP
-4-keto-L-rhamnos
e
4-keto-reductase;Tr
ifunctional
UDP-glucose
4,6-dehydratase/U
DP-4-keto-6-deoxy
-D-glucose
3,5-epimerase/UDP
-4-keto-L-rhamnos
e-reductase
RHM3;UDP-glucos
e
4,6-dehydratase;U
DP-4-keto-6-deoxy
-D-glucose
3,5-epimerase/UDP
-4-keto-L-rhamnos
e
4-keto-reductase;Tr
ifunctional
UDP-glucose
4,6-dehydratase/U
DP-4-keto-6-deoxy
-D-glucose
3,5-epimerase/UDP
-4-keto-L-rhamnos
e-reductase
RHM2;UDP-glucos
e
4,6-dehydratase;U
DP-4-keto-6-deoxy
-D-glucose
3,5-epimerase/UDP

		-4-keto-L-rhamnose 4-keto-reductase																	
Q9SZD4; Q9SL67	1;1	26S proteasome regulatory subunit 4 homolog A;26S proteasome regulatory subunit 4 homolog B	RPT2A;RPT2B	2	1	4.7	49.369	443	18.046	0	0	0	0	0	0	0	0	0	940240
Q9SZN1; Q40079; Q40078; Q43432; Q43433	2;1;1;0;0;0;0	V-type proton ATPase subunit B2	VHA-B2	7	2	6.6	54.304	487	323.31	6446500	6990200	6905300	7047800	7391800	7113600	9614800	10647000	11742000	12468000
Q9SZX3	1	Argininosuccinate synthase, chloroplastic		1	1	4	53.845	494	7.3269	0	0	0	0	0	0	0	0	0	900760
Q9T029; Q8GYL5;Q9SIW5	1;1;1	40S ribosomal protein S25-4;40S ribosomal protein S25-3;40S ribosomal protein S25-1	RPS25E;RPS25D;RPS25A	3	1	13	12.053	108	6.7686	0	0	0	0	0	0	0	0	0	322800
Q9T041	1	Microtubule-associated protein TORTIFOLIA1	TOR1	1	1	1.7	94.003	864	6.1137	0	0	0	0	0	0	0	0	0	614450
Q9T0E0	1	Putative ATPase, plasma membrane-like		1	1	1.7	90.438	813	25.678	7382000	7035100	9148100	7352600	6781100	9028600	6114300	6294900	6321100	6656300
Q9T4F6	1			1	1	1.9	48.313	431	5.9878	0	0	0	0	0	0	0	0	0	4972900
Q9XEA8;Q9XEA6;P38076	1;0;0			3	1	5.8	34.306	325	10.09	0	0	0	0	0	0	0	0	0	1574200
Q9XEK6;P46295; Q9SIH0; Q9CAX6;P42036	1;1;1;1;1	40S ribosomal protein S14-1;40S ribosomal protein S14-2;40S ribosomal protein S14-3	RPS14A;RPS14B;RPS14C	5	1	9.7	14.416	134	5.8893	0	0	0	0	0	0	0	0	0	8342100
Q9XF41; Q9FR39; Q94JN2; Q5VMJ3; Q5EF31; P83647;P	1;1;1;1;1;1;1;1;1;1;1;1			12	1	9.9	14.052	131	6.861	0	0	0	0	0	0	0	0	6584800	0

52184;P49234;P49233;P49232;O22655;A4KA43																			
Q9XF61	1			1	1	3.2	57.089	507	6.2893	0	0	0	0	0	0	0	0	2033500	0
Q9XFT3	1	Oxygen-evolving enhancer protein 3-1, chloroplastic	PSBQ1	1	1	6.2	23.866	224	6.3974	0	0	0	0	1796800	0	0	0	0	0
Q9XG77; O48551; O81147; O81146	2;2;1;1	Proteasome subunit alpha type-6-B;Proteasome subunit alpha type-6-A	PAA2; PAA1	4	2	9.3	27.303	246	11.733	3349800	2943100	3441000	3730900	3349800	0	1625100	0	0	0
Q9XGX1;P24825; Q9XGX2;Q9SBL7;Q9SBL3	1;1;1;1;1			5	1	3.2	43.576	400	6.3516	0	0	3065500	0	0	0	0	0	0	0
Q9XGX8	1			1	1	11.8	10.878	93	6.208	0	0	0	0	0	0	0	0	0	2341100
Q9XQ94; P25462;P14655;P08281;P13564	1;1;1;1;1;1;1;1			8	1	3.3	47.115	428	5.9327	0	0	0	0	0	0	0	0	0	587680
Q9XQA8;Q9MTN1;Q9BBT0;Q8S8X8;Q7YJX7;Q70Y08;Q6L3B1;Q6EW53;Q6ENY1;Q6ENJ1;Q68S11;Q56P05;Q4VZN8;Q4FFP4;Q4FFP2;Q4F	2;2;2;2;2;2;2;2;2;2;2;2;2;2;2;2;2;2;2;2;2;2;2;2;2;2;2;2;2;2;2;2;2;2;2;2;2;2;2;2;2;2;2;2;2;2;2;2;2;2;2;2;2;2;2;2;2;2;2;2;2;2;2;2;	Photosystem II D2 protein	psbD	116	2	9.9	39.656	353	12.222	3190700	0	3022500	0	3114700	0	0	0	0	0

FP1;Q49	2;2;2;2;
L03;Q3V	2;2;2;2;
539;Q3C	2;2;2;2;
1I4;Q3B	2;2;2;2;
AP5;Q36	2;2;2;2;
814;Q33	2;2;2;2;
C42;Q2V	2;1;1;1;
EI1;Q2P	1;1;1;1;
MT8;Q2	1;1;1;1;
MIJ2;Q2	1;1;1;1;
MIA5;Q2	1;1;1;1;
L902;Q1	1;1;1;1;
KXW4;Q	1;1;1;1
14FG2;Q	
0ZJ25;Q0	
G9W7;Q	
0G9M4;	
Q09X22;	
Q09MI3;	
Q09G51;	
Q09FW6;	
Q06RD6;	
Q06H02;	
Q06FW1;	
P69686;P	
69685;P4	
8184;P11	
849;P108	
03;P0C43	
7;P0C436	
;P0C435;	
P06005;B	
5LMM1;	
B3TNB8;	
B2Y1U5;	
B2XWK	
0;B2LMI	
8;B1VK	
H0;B1N	
WE5;B1	
A930;B0	
Z5C4;B0	

Z540;B0
Z4V6;B0
Z4M2;A9
L991;A8
Y9F5;A8
W3B9;A
8SE98;A
7Y3C4;A
7M961;A
6MMT9;
A6MMK
2;A6MM
B7;A6M
M31;A6
H5G6;A4
QLS8;A4
QLI9;A4
QLA1;A
4QL14;A
4QKS6;A
4QKI7;A
4QKA0;
A4QK13;
A4QJS8;
A4QJJ4;
A4QJB0;
A4GYQ4
;A4GGA
2;A1E9Z
3;A1E9Q
9;A0ZZ3
0;A0A33
0;P56761
;Q9TL00;
Q3ZJ46;
Q20EU7;
Q0P3Q0;
Q06SH5;
P56319;P
06007;A6
YG76;Q8
5WW5;Q

49CA8;P41644;A8W3I2;A7M8Z6;Q8WI19;Q8M9W4;Q85FM2;Q85AC9;Q6YXN8;Q32RT0;Q32RM5;Q1KVW6;P06404;P06006;A2T330;Q9MUW2;Q19VC7;Q06GR5																			
Q9ZNT7;Q9SIL6	1;1	Prohibitin-2, mitochondrial;Prohibitin-6, mitochondrial	PHB2;PHB6	2	1	3.8	31.81	286	5.8795	0	0	0	0	0	0	0	275210	0	0
Q9ZNZ7;Q43155;Q9T0P4	2;1;1	Ferredoxin-dependent glutamate synthase 1, chloroplastic/mitochondrial;Ferredoxin-dependent glutamate synthase 2, chloroplastic	GLU1;GLU2	3	2	2	176.75	1622	37.288	0	0	0	0	0	2366600	2500900	2529000	2302600	2063100
																			1029600
Q9ZPN8	1			1	1	3.4	50.107	446	19.449	0	0	0	0	0	0	0	0	0	0
Q9ZRD6	2	VAMP-like protein YKT61	YKT61	1	2	13.6	22.543	199	11.361	0	0	0	0	0	0	0	2490000	0	0
Q9ZSE4;Q0DBD3;A2YEB4;Q0E2S4;A2X2G3;Q07099;Q07098;	1;1;1;1;1;1;1;1;1;0;0;0;0;0;0	Serine/threonine-protein phosphatase PP2A-1 catalytic subunit;Serine/threonine-protein phosphatase PP2A-2 catalytic	PP2A1;PP2A2;PP2A5;PPX1;PPX2;PP2A4	15	1	6.9	34.968	306	7.5667	0	0	0	0	0	0	0	0	0	1025800

O04951; P48579;P48577;P23778;O04860;P48529;P48528;Q07100		subunit;Serine/threonine-protein phosphatase PP2A-5 catalytic subunit;Serine/threonine-protein phosphatase PP-X isozyme 1;Serine/threonine-protein phosphatase PP-X isozyme 2;Serine/threonine-protein phosphatase PP2A-4 catalytic subunit																	
Q9ZSR8; Q08682; O22518	1;1;1	40S ribosomal protein Sa-1	RPSaA	3	1	5.8	32.03	292	32.384	16518000	18907000	12133000	14592000	15067000	22595000	26276000	23965000	23776000	22501000
Q9ZSW9;Q6DUX3;Q5J907;Q2PS27;P43349;P31265;P85905;Q9ZRX0;Q8H6A5;Q9XHL7;Q9M5I8;Q9M5G3; Q944W6; Q944T2; Q8LRM8;P35681; O03992	2;2;2;2;2;2;1;1;1;1;1;1;1;1;1;1;1	Translationally-controlled tumor protein homolog	TCTP	17	2	17.9	19.038	168	93.324	26182000	25065000	24200000	27799000	31941000	75337000	1.34E+08	1.11E+08	73214000	71665000
Q9ZT91	2	Elongation factor Tu, mitochondrial	TUFA	1	2	4.6	49.409	454	12.954	5531100	5609100	5689400	5780400	5462500	0	0	838810	825190	852430
Q9ZTR1	1			1	1	4.8	36.7	334	7.571	0	0	0	0	0	0	0	0	0	1104400
Q9ZTS1	2			1	2	3.1	89.452	801	11.376	0	0	0	0	0	0	0	0	0	450620
Q9ZTT5	1			1	1	9.7	29.145	259	11.877	0	0	0	0	0	0	0	0	0	3355300
Q9ZTW3;Q9MAS	1;1;1;1	Vesicle-associated membrane protein	VAMP721;	4	1	5.5	24.765	219	6.9152	0	0	0	0	0	0	0	0	0	1451300

5;P47192;O48850		721;Putative vesicle-associated membrane protein 726;Vesicle-associated membrane protein 722;Vesicle-associated membrane protein 725	VAMP726;VAMP722;VAMP725																
Q9ZU52	1	Probable fructose-bisphosphate aldolase 3, chloroplastic	FBA3	1	1	3.6	42.327	391	14.9	0	0	0	0	0	0	0	0	0	11287000
Q9ZUY6;Q9SGE0	2;2	UDP-D-apiose/UDP-D-xylose synthase 1;UDP-D-apiose/UDP-D-xylose synthase 2	AXS1;AXS2	2	2	4.9	43.637	389	11.828	0	0	0	1360300	1638900	0	2039000	2266400	2042900	2217700
Q9ZVX8;P93004;Q9FF53	1;1;1	Probable aquaporin PIP2-8;Aquaporin PIP2-7;Aquaporin PIP2-7, N-terminally processed;Probable aquaporin PIP2-4;Probable aquaporin PIP2-4, N-terminally processed	PIP2-8;PIP2-7;PIP2-4	3	1	4.3	29.5	278	10.714	0	0	0	0	0	0	0	0	0	11234000
S8CE21	1	Peptidyl-prolyl cis-trans isomerase	M569_09669	1	2	30.8	18.064	172	83.284	1454400	2033300	1672200	1397200	1423300	3228900	2340900	2881200	3171400	2832600
S8E148	1	Pyruvate dehydrogenase E1 component subunit alpha	M569_08768	1	1	3.7	41.637	377	45.633	7897900	8175000	5292100	4759200	7949900	2273200	3350300	2171200	2374000	2538500
T1E156	1	ATP synthase subunit gamma	ATP3	1	2	17	20.685	188	38.84	3134700	5697000	3883200	3032700	3808500	2332900	2551200	2203300	2042500	1664900
V7BP31	1	Lactoylglutathione lyase	PHAVU_006G149400g	1	3	13.6	32.35	287	24.63	12414000	15187000	11878000	11429000	11898000	7424600	6894300	6071000	5956300	6383000

Table S2. The information of identified differentially expressed proteins by MaxQuant

	protein	Protein names	Gene names	Unique peptides	Mol. weight [kDa]	Sequence length	LFQ intensity 50_R1	LFQ intensity 50_R2	LFQ intensity 50_R3	LFQ intensity 50_R4	LFQ intensity 50_R5	LFQ intensity CK_R1	LFQ intensity CK_R2	LFQ intensity CK_R3	LFQ intensity CK_R4	LFQ intensity CK_R5	mean 50	sd 50	mean ck	sd ck	ratio 50/ck	p_value 50/ck
52	Q76H85	Histone H4	SlH4	3	11.409	103	35872000	36181000	34461000	35110000	36101000	247480000	275520000	218140000	260420000	292780000	35545000	739060.5523	258868000	28364494	0.1373094	1.11E-07
1	Q9ZT91	Elongation factor Tu, mitochondrial	TUFA	2	49.409	454	5531100	5609100	5689400	5780400	5462500	0	0	838810	825190	852430	5614500	125719.4695	838810	13620	6.6934109	9.716E-09
11	P30707	60S ribosomal protein L9	RPL9	2	21.752	193	18684000	20400000	15085000	29623000	28684000	6007200	6217400	5369200	6559400	7780500	22495200	6382259.608	6386740	891696.8	3.5221725	0.0005166
47	Q39471	Isopentenyl-diphosphate Delta-isomerase II	IPI2	6	32.561	286	25266000	18569000	27743000	25822000	22822000	8558100	8582200	9171800	8147000	9509200	24044400	3528671.039	8793660	541608.8	2.7342881	1.194E-05
12	P51430	40S ribosomal protein S6-2	RPS6B	2	28.162	249	21987000	19666000	24036000	23154000	18520000	0	9950400	10235000	6879300	0	21472600	2326176.219	9021566.67	1860706.6	2.3801409	0.0002108
13	O81361	40S ribosomal protein S8	RPS8	2	24.721	221	42368000	41795000	39853000	48482000	38660000	17673000	17724000	18474000	15515000	19488000	42231600	3797722.647	17774800	1461469.7	2.3759255	9.004E-07
26	O49845	Sucrose synthase 4	SUS4	2	91.649	801	5574700	4736700	5926000	6621600	4542700	2323200	2410800	2367000	0	0	5480340	857636.3233	2367000	43800	2.3153105	0.0003875
43	P85929	Nucleoside diphosphate kinase 1	NDK1	2	1.8881	18	105990000	104190000	104240000	146100000	125050000	54518000	57499000	68567000	58017000	57546000	117114000	18436928.43	59229400	5401067	1.9772951	0.000147
44	C6TBN2	Probable aldo-keto reductase 1	AKR1	3	38.252	346	10344000	10374000	8581600	10613000	9305500	4656400	4600700	6089900	5141500	4524800	9843620	866876.0419	5002660	654061.42	1.9676772	8.693E-06
55	Q43873	Peroxidase 73	PER73	2	35.927	329	11189000	11245000	10883000	12200000	11827000	6259700	6115700	6135400	6008800	6261100	11468800	532563.799	6156140	106672.27	1.8629856	2.015E-08
45	Q9SU63	Aldehyde dehydrogenase family 2 member B4, mitochondrial	ALDH2B4	2	58.588	538	18245000	15771000	15832000	13975000	15177000	8671600	9181600	9253500	9174600	9915800	15800000	1557132.3	9239420	443748.94	1.7100641	1.764E-05
41	A7PZL3	Probable polygalacturonase	GSVIVT00026920001	2	53.489	491	2291000	2080700	2082000	1918100	3084000	1456200	1348000	1287100	1305400	1346700	2291160	462550.0978	1348680	65631.905	1.6988166	0.0019733

46	P31426	Phenylalanine ammonia-lyase 2	PAL-2	2	64.143	590	3795800	3623800	3892200	3874800	3481300	2285700	2206700	2173300	2413000	0	3733580	176544.3061	2269675	106540.83	1.6449844	0.0032274
48	Q9S7A0	Probable glutamate dehydrogenase 3	GSH3	2	44.527	411	4547500	5626100	3961000	4749900	4262800	3004000	3055400	2886900	0	0	4629460	631623.4741	2982100	86358.381	1.5524161	0.0067403
14	Q9SXU1	Proteasome subunit alpha type-7	PAD1	4	27.096	249	31425000	30205000	28503000	19660000	26229000	20116000	16579000	22774000	16485000	14557000	27204400	4646848.48	18102200	3293365.4	1.5028229	0.0072565
2	Q9SEI3	26S protease regulatory subunit 10B homolog A	RPT4A	5	44.816	399	4360100	4209900	4874700	4438000	5309900	7700300	6120600	6500200	7880800	7042400	4638520	449436.6385	7048860	754856.67	0.6580525	0.0002786
28	F4JLP5	Dihydrolipoyl dehydrogenase 2, chloroplastic	LPD2	2	60.144	567	3392900	2559700	2421300	2770600	2781500	1936200	1678200	1734400	0	0	2785200	371752.4714	1782933.33	135674.66	1.5621448	0.0064423
3	P54778	26S protease regulatory subunit 6B homolog	RPT3	3	46.532	413	2432400	2515300	4224700	3390200	2688300	5688600	5034000	5294400	5621000	4366400	3050180	757267.5729	5200880	535565.92	0.5864738	0.0008377
29	Q94KU2	6-phosphogluconate dehydrogenase, decarboxylating 2, chloroplastic	pgdP	3	58.293	537	14471000	13691000	16235000	14354000	13492000	7818400	9689400	6831700	8747900	7378100	14448600	1082787.745	8093100	1135203.9	1.7852986	1.767E-05
56	Q93VR3	GDP-mannose 3,5-epimerase	At5g28840	5	42.758	377	94235000	94277000	97263000	99841000	93138000	49825000	48625000	47743000	51955000	53443000	95750800	2752859.64	50318200	2355064.6	1.9029059	2.824E-09
17	Q949X7	Diaminopimelate decarboxylase 1, chloroplastic	LYSA1	4	53.557	484	2500700	1897700	2148600	2686600	2737600	4316400	4731100	5331100	4028000	4629600	2394240	361107.7305	4607240	489877.91	0.519669	3.884E-05
15	Q9MTJ8	ATP-dependent Clp protease proteolytic subunit	clpP	3	27.956	249	0	2405800	2345400	2140000	2298400	5344000	3897700	6009200	3957100	3823700	2297400	113769.2401	4606340	1006027.7	0.4987474	0.0026337
16	P68173	Adenosylhomocysteinase	SAHH	2	53.103	485	77461000	70997000	70767000	65837000	60016000	33257000	35520000	33990000	30136000	29067000	69015600	6507964.874	32394000	2703301.4	2.1305056	2.738E-06
30	Q9SJB3	ATPase 5, plasma membrane-type	AHA5	2	104.74	949	12996000	13264000	11291000	11946000	11069000	5919100	5871300	4958900	5849500	5565200	12113200	987167.5136	5632800	401409.95	2.1504758	8.227E-07
27	P54243	Glucose-6-phosphate isomerase, cytosolic	PGIC	7	62.685	568	17002000	16643000	18891000	14134000	15305000	8599200	9926900	10238000	12917000	10650000	16395000	1800013.194	10466220	1570733.3	#DIV/0!	0.0005414
39	P30184	Leucine aminopeptidase 1	LAP1	2	54.509	520	7612200	7612400	7185900	8070400	8224100	3151000	3299100	3387200	3131000	3325300	774100	413265.4	325872	112346	2.37547	1.181E-08

																	0	655	0	.06	26	
10	Q9LTX9	Heat shock 70 kDa protein 7, chloroplastic	HSP70-7	2	76.996	718	2538200	2691300	2609300	2430000	2637500	1011700	1058500	1177400	1010400	1058200	2581260	100984.8157	1063240	68060.289	2.4277303	2.963E-09
31	Q9LU41	Calcium-transporting ATPase 9, plasma membrane-type	ACA9	2	118.77	1086	2046800	1881600	2082400	2056500	0	640980	652930	646955	0	0	2188837.5	91393.81361	646955	5975	3.3832917	0.0225673
4	O04308	Probable mitochondrial-processing peptidase subunit alpha-2	MPPA2	2	54.053	499	3779300	4206400	4547200	4817200	5600900	17853000	17614000	15052000	15383000	15453000	4590200	685520.5577	16271000	1346291.4	0.2821093	1.276E-07
32	P37829	fructokinase	N/A	3	33.764	319	24418000	24465000	21627000	22622000	22613000	7479000	5304800	5389800	5622300	7246100	23149000	1247369.833	6208400	1063174.2	3.7286579	1.304E-08
18	Q940P8	T-complex protein 1 subunit beta	CCT2	2	57.285	527	1805400	1751200	1639500	1905400	2096100	8846500	9566100	9434200	7992300	7501200	1839520	172587.7081	8668060	900486.12	0.2122182	1.709E-07
21	P93819	Malate dehydrogenase, cytoplasmic 1	MDH1	4	35.571	332	27276000	23121000	34762000	36533000	38624000	18957000	13414000	12350000	10512000	14993000	32063200	6580025.737	14045200	3192950.2	2.2828582	0.0005679
5	S8CE21	Peptidyl-prolyl cis-trans isomerase	M569_09669	2	18.064	172	1454400	2033300	1672200	1397200	1423300	3228900	2340900	2881200	3171400	2832600	1596080	267598.1072	2891000	353170.35	0.5520858	0.0001814
6	A8MRZ7	Translational initiation factor 4A-1	EIF4A1	3	41.85	369	2041100	2660800	1381900	1857400	1855700	3513600	3496000	3501600	2866400	2802100	1959380	461871.4074	3235940	367450.16	0.6055057	0.0012941
7	Q9FZ48	Ubiquitin-conjugating enzyme E2 36	UBC36	4	17.192	153	723770	685810	832020	718250	765020	1016400	1320800	1213000	1234000	1233800	744974	56220.56768	1203600	112588.01	0.6189548	3.822E-05
8	G7IRR6	Protein disulfide-isomerase	MTR_2g094180	2	63.018	564	1846200	1914000	1931400	1734000	1754500	2555700	2669800	2632600	2536400	3898500	1836020	89910.29974	2858600	583882.42	0.6422794	0.0047379
9	J7KE88	Heat shock protein 90	HSP90	2	80.271	699	6862900	5518300	6082200	6132900	4478900	3510000	4144600	3687500	3455600	3908800	5815040	886530.4214	3741300	286673.05	1.5542833	0.0010838
19	Q9LXS7	Citrate synthase 1, peroxisomal	CSY1	2	52.895	480	3930500	3720900	3919700	4467000	3720300	1159000	1097000	1291700	1448600	1232100	3951680	305704.9754	1245680	135183.16	3.1723075	8.906E-08
20	S8E148	Pyruvate dehydrogenase E1 component subunit alpha	M569_08768	1	41.637	377	7897900	8175000	5292100	4759200	7949900	2273200	3350300	2171200	2374000	2538500	6814820	1647407.684	2541440	472051.28	2.6814798	0.0005248

22	M0TRQ8	Succinyl-CoA ligase subunit beta	N/A	2	45.009	421	820130	1351800	784120	806490	785880	613720	583000	592160	555610	592130	909684	247603.1232	587324	21022.061	1.5488623	0.0198688
23	T1E156	ATP synthase subunit gamma	ATP3	2	20.685	188	3134700	5697000	3883200	3032700	3808500	2332900	2551200	2203300	2042500	1664900	3911220	1069477.376	2158960	333065.91	1.8116223	0.0081036
24	Q7M2G6	ATP synthase subunit alpha	ATP1	3	53.323	491	37336000	28937000	38247000	47698000	40469000	24138000	25799000	21328000	25147000	25691000	38537400	6733183.593	24420600	1849702.5	1.5780693	0.0019482
25	Q9FKK7	Xylose isomerase	XYLA	2	53.72	477	3031500	2507900	2984900	2983000	3088400	2087600	1516600	1340600	1410000	1584300	2919140	233877.3888	1587820	294789.08	1.8384578	4.732E-05
33	B9T118	NADH-ubiquinone oxidoreductase, putative	RCOM_0458390	3	80.768	744	778470	695050	707620	756700	705380	1218000	1438000	995690	1075600	1363000	728644	36679.51922	1218058	186496.97	0.5982014	0.0004252
34	P40392	Ras-related protein RIC1	RIC1	1	22.475	202	1082700	1418900	1097700	1169700	1014100	3246000	3830500	3326500	3289900	2989500	1156620	156692.0292	3336480	306148.17	0.3466588	5.977E-07
35	O80501	Ras-related protein RABH1b	RABH1B	4	23.13	208	5915400	3625600	4657200	3928300	4601900	12624000	12066000	12734000	11425000	10246000	4545680	883096.1935	11819000	1021741.7	0.3846078	2.086E-06
36	P11574	V-type proton ATPase subunit B1	VHA-B1	15	54.11	486	18800000	18232000	16731000	16808000	15701000	9831400	9281000	9382600	10644000	8106700	17254400	1248083.851	9449140	923127.42	1.8260286	3.517E-06
37	B7SDI4	Aquaporin	N/A	2	30.759	289	2318400	2113200	2359900	2467900	2261400	3598100	3606400	3865500	4270200	3689300	2304160	130780.9734	3805900	280937.4	0.6054179	4.646E-06
38	B6T451	Importin subunit alpha	N/A	3	57.879	527	914240	826100	926750	893010	860130	1341100	2134900	1343000	1516600	1403200	884046	41065.1206	1547760	335870.86	0.5711777	0.0023299
40	Q9LXC0	GDP dissociation inhibitor	At5g09550	3	49.549	445	1768600	2520700	2489000	2510700	1551400	849940	1050800	842000	839390	938400	2168080	470265.2092	904106	91753.037	2.3980374	0.0003622
42	V7BP31	Lactoylglutathione lyase	PHAVU_006G149400g	3	32.35	287	12414000	15187000	11878000	11429000	11898000	7424600	6894300	6071000	5956300	6383000	12561200	1508721.876	6545840	610980.94	1.9189592	3.456E-05
49	B9RT61	Translationally-controlled tumor protein homolog	RCOM_0681260	2	18.962	168	1595200	2243300	1705700	1487400	1768700	5331400	5017100	4639900	4285200	3887400	1760060	290720.1627	4632200	572761.73	0.379962	8.497E-06
50	O04331	Prohibitin-3, mitochondrial	PHB3	3	30.4	277	2929000	2880100	2826200	2367000	2709400	5114200	3647400	4828200	4814600	4777800	2742340	225176.2821	4636440	568979.41	0.5914754	0.0001219

51	Q0WM29	Methylmalonate-semialdehyde dehydrogenase [acylating], mitochondrial	ALDH6B2	2	65.927	607	483630	458640	591950	502440	459990	907600	798770	627430	854390	722350	499330	54858.76913	782108	110393.45	0.6384412	0.0008968
53	D7LSV8	ADP-ribosylation factor	ARALYDRAFT_486735	9	21.585	189	20973000	24493000	23539000	23302000	21816000	42248000	41494000	49589000	48209000	46034000	22824600	1411340.958	45514800	3569530.9	0.5014764	1.023E-06
54	D7UC38	Phosphomannomutase	VIT_15s0046g03520	1	28.114	249	1501000	1587400	1334300	1300900	1389300	664990	745090	577410	642260	757130	1422580	119396.6792	677376	74708.825	2.1001335	2.388E-06
57	A5B8T3	Putative uncharacterized protein	VIT_05s0102g00710	1	35.199	330	1336700	1486600	1331500	1311100	1407300	3150200	2022700	1915000	1946100	1867800	1374640	72352.52587	2180360	545076.54	0.6304647	0.0112453
58	D7SW76	Uncharacterized protein	VIT_07s0031g01740	2	27.683	240	1389800	1024000	1209800	1249700	991190	1712500	1602200	2206900	2026700	2001800	1172898	165450.2902	1910020	246952.84	0.6140763	0.0005442

Table S3. KEGG pathway analysis of 58 DEPs

Pathway Name	Pathway ID	Pvalue	Pvalue_ adjusted	Genes	Count	Pop Hit	List_Total	Background Genes	URL
Citrate cycle (TCA cycle)	ath00020	1.91E-05	9.54E-04	LPD2|1.56;At2g20420|1.55;E1 ALPHA|2.68;MDH1|2.28;At3g13930|0.61;CSY1|3.17	6	63	44	4805	http://www.kegg.jp/kegg-bin/show_pathway?ath00020/ath:AT4G16155%09red/ath:AT2G20420%09red/ath:AT1G59900%09red/ath:AT1G04410%09red/ath:AT3G13930%09blue/ath:AT3G58740%09red
Carbon metabolism	ath01200	9.33E-05	1.65E-03	LPD2|1.56;GSH3|1.55;At2g20420|1.55;E1 ALPHA|2.68;At5g41670|1.79;MDH1|2.28;PGI1|1.57;ALDH6B2|0.64;At3g13930|0.61;CSY1|3.17	10	262	44	4805	http://www.kegg.jp/kegg-bin/show_pathway?ath01200/ath:AT4G16155%09red/ath:AT3G03910%09red/ath:AT2G20420%09red/ath:AT1G59900%09red/ath:AT5G41670%09red/ath:AT1G04410%09red/ath:AT4G24620%09red/ath:AT2G14170%09blue/ath:AT3G13930%09blue/ath:AT3G58740%09red
Pyruvate metabolism	ath00620	9.89E-05	1.65E-03	LPD2|1.56;ALDH2B4|1.71;E1 ALPHA|2.68;MDH1|2.28;GLX1|1.92;At3g13930|0.61	6	84	44	4805	http://www.kegg.jp/kegg-bin/show_pathway?ath00620/ath:AT4G16155%09red/ath:AT3G48000%09red/ath:AT1G59900%09red/ath:AT1G04410%09red/ath:AT1G11840%09red/ath:AT3G13930%09blue
Fructose and mannose metabolism	ath00051	2.61E-03	2.46E-02	PMM|2.1;At3g59480|0.63;At3g59480|0.63;XYLA|1.84	4	64	44	4805	http://www.kegg.jp/kegg-bin/show_pathway?ath00051/ath:AT2G45790%09red/ath:AT3G59480%09blue/ath:AT3G59480%09blue/ath:AT5G57655%09red
Glycolysis / Gluconeogenesis	ath00010	4.00E-03	2.85E-02	LPD2|1.56;ALDH2B4|1.71;E1 ALPHA|2.68;PGI1|1.57;At3g13930|0.61	5	117	44	4805	http://www.kegg.jp/kegg-bin/show_pathway?ath00010/ath:AT4G16155%09red/ath:AT3G48000%09red/ath:AT1G59900%09red/ath:AT4G24620%09red/ath:AT3G13930%09blue
Amino sugar and nucleotide sugar metabolism	ath00520	7.32E-03	4.58E-02	PMM|2.1;At3g59480|0.63;PGI1|1.57;At3g59480|0.63;At5g28840|1.9	5	135	44	4805	http://www.kegg.jp/kegg-bin/show_pathway?ath00520/ath:AT2G45790%09red/ath:AT3G59480%09blue/ath:AT4G24620%09red/ath:AT3G59480%09blue/ath:AT5G28840%09red
Oxidative phosphorylation	ath00190	1.54E-02	7.08E-02	ATPA|1.58;ATPC|1.81;EMB1467|0.6;AHA5|2.15;VHA-B1|1.83	5	162	44	4805	http://www.kegg.jp/kegg-bin/show_pathway?ath00190/ath:ArthMp100%09red/ath:AT2G33040%09red/ath:AT5G37510%09blue/ath:AT2G24520%09red/ath:AT1G76030%09red
Proteasome	ath03050	1.56E-02	7.08E-02	PAD2|1.5;RPT4A|0.66;RPT3|0.59	3	58	44	4805	http://www.kegg.jp/kegg-bin/show_pathway?ath03050/ath:AT5G66140%09red/ath:AT5G43010%09blue/ath:AT5G58290%09blue
Biosynthesis of	ath01110	2.23E-02	9.31E-02	PAL1|1.64;PMM|2.1;LPD2|1.56;PER73|1.86;ALDH2B4|1.71;LYSA1|0.5	16	1066	44	4805	http://www.kegg.jp/kegg-bin/show_pathway?

secondary metabolites				2;IPP2|2.73;NDK1|1.98;At2g20420|1.55;E1 ALPHA|2.68;At5g41670|1.79;MDH1|2.28;PGI1|1.57;At3g13930|0.61;At5g28840|1.9;CSY1|3.17					ath01110/ath:AT2G37040%09red/ath:AT2G45790%09red/ath:AT4G16155%09red/ath:AT5G67400%09red/ath:AT3G48000%09red/ath:AT3G14390%09blue/ath:AT3G02780%09red/ath:AT4G09320%09red/ath:AT2G20420%09red/ath:AT1G59900%09red/ath:AT5G41670%09red/ath:AT1G04410%09red/ath:AT4G24620%09red/ath:AT3G13930%09blue/ath:AT5G28840%09red/ath:AT3G58740%09red
Glyoxylate and dicarboxylate metabolism	ath00630	2.96E-02	1.14E-01	LPD2|1.56;MDH1|2.28;CSY1|3.17	3	74	44	4805	http://www.kegg.jp/kegg-bin/show_pathway?ath00630/ath:AT4G16155%09red/ath:AT1G04410%09red/ath:AT3G58740%09red
beta-Alanine metabolism	ath00410	5.13E-02	1.79E-01	ALDH2B4|1.71;ALDH6B2|0.64	2	40	44	4805	http://www.kegg.jp/kegg-bin/show_pathway?ath00410/ath:AT3G48000%09red/ath:AT2G14170%09blue
Ascorbate and aldarate metabolism	ath00053	5.36E-02	1.79E-01	ALDH2B4|1.71;At5g28840|1.9	2	41	44	4805	http://www.kegg.jp/kegg-bin/show_pathway?ath00053/ath:AT3G48000%09red/ath:AT5G28840%09red
Pentose phosphate pathway	ath00030	9.82E-02	3.07E-01	At5g41670|1.79;PGI1|1.57	2	58	44	4805	http://www.kegg.jp/kegg-bin/show_pathway?ath00030/ath:AT5G41670%09red/ath:AT4G24620%09red
Starch and sucrose metabolism	ath00500	1.12E-01	3.29E-01	At3g59480|0.63;SUS4|2.32;PGI1|1.57;At3g59480|0.63	4	202	44	4805	http://www.kegg.jp/kegg-bin/show_pathway?ath00500/ath:AT3G59480%09blue/ath:AT3G43190%09red/ath:AT4G24620%09red/ath:AT3G59480%09blue
Lysine biosynthesis	ath00300	1.37E-01	3.81E-01	LYSA1|0.52	1	16	44	4805	http://www.kegg.jp/kegg-bin/show_pathway?ath00300/ath:AT3G14390%09blue
Histidine metabolism	ath00340	1.53E-01	4.02E-01	ALDH2B4|1.71	1	18	44	4805	http://www.kegg.jp/kegg-bin/show_pathway?ath00340/ath:AT3G48000%09red
Pentose and glucuronate interconversions	ath00040	1.69E-01	4.23E-01	ALDH2B4|1.71;XYLA|1.84	2	81	44	4805	http://www.kegg.jp/kegg-bin/show_pathway?ath00040/ath:AT3G48000%09red/ath:AT5G57655%09red

Table S4. PPI analysis of enriched 13 proteins

Node1	Node2	Type	Score
Citrate cycle (TCA cycle)	LPD2	kegg2pro	
Citrate cycle (TCA cycle)	At2g20420	kegg2pro	
Citrate cycle (TCA cycle)	E1 ALPHA	kegg2pro	
Citrate cycle (TCA cycle)	MDH1	kegg2pro	
Citrate cycle (TCA cycle)	At3g13930	kegg2pro	
Citrate cycle (TCA cycle)	CSY1	kegg2pro	
Carbon metabolism	LPD2	kegg2pro	
Carbon metabolism	GSH3	kegg2pro	
Carbon metabolism	At2g20420	kegg2pro	
Carbon metabolism	E1 ALPHA	kegg2pro	
Carbon metabolism	At5g41670	kegg2pro	
Carbon metabolism	MDH1	kegg2pro	
Carbon metabolism	PGI1	kegg2pro	
Carbon metabolism	ALDH6B2	kegg2pro	
Carbon metabolism	At3g13930	kegg2pro	
Carbon metabolism	CSY1	kegg2pro	
Pyruvate metabolism	LPD2	kegg2pro	
Pyruvate metabolism	ALDH2B4	kegg2pro	
Pyruvate metabolism	E1 ALPHA	kegg2pro	
Pyruvate metabolism	MDH1	kegg2pro	
Pyruvate metabolism	GLX1	kegg2pro	
Pyruvate metabolism	At3g13930	kegg2pro	
Fructose and mannose metabolism	PMM	kegg2pro	
Fructose and mannose metabolism	At3g59480	kegg2pro	
Fructose and mannose metabolism	XYLA	kegg2pro	
Propanoate metabolism	LPD2	kegg2pro	
Propanoate metabolism	At2g20420	kegg2pro	
Propanoate metabolism	ALDH6B2	kegg2pro	
Glycolysis / Gluconeogenesis	LPD2	kegg2pro	
Glycolysis / Gluconeogenesis	ALDH2B4	kegg2pro	
Glycolysis / Gluconeogenesis	E1 ALPHA	kegg2pro	
Glycolysis / Gluconeogenesis	PGI1	kegg2pro	
Glycolysis / Gluconeogenesis	At3g13930	kegg2pro	
Amino sugar and nucleotide sugar metabolism	PMM	kegg2pro	
Amino sugar and nucleotide sugar metabolism	At3g59480	kegg2pro	
Amino sugar and nucleotide sugar metabolism	PGI1	kegg2pro	
Amino sugar and nucleotide sugar metabolism	At5g28840	kegg2pro	
LPD2	E1 ALPHA	PPI	998
LPD2	At2g20420	PPI	977
LPD2	MDH1	PPI	473

LPD2	PGI1	PPI	426
LPD2	CSY1	PPI	795
ALDH2B4	MDH1	PPI	522
GSH3	E1 ALPHA	PPI	682
GSH3	At5g41670	PPI	562
GSH3	CSY1	PPI	932
GSH3	MDH1	PPI	933
E1 ALPHA	CSY1	PPI	795
E1 ALPHA	MDH1	PPI	512
E1 ALPHA	PGI1	PPI	932
At5g41670	MDH1	PPI	662
At5g41670	PGI1	PPI	672
At5g41670	CSY1	PPI	567
MDH1	CSY1	PPI	994
MDH1	PGI1	PPI	703
PGI1	CSY1	PPI	795
